# Rapid Electrochemical Assessment of Excited-State Quenching Dynamics

**DOI:** 10.1021/acscatal.5c02778

**Published:** 2025-09-23

**Authors:** Tobia Casadei, Alberto Piccoli, Davide Zeppilli, Laura Orian, Abdirisak A. Isse, Marco Fantin

**Affiliations:** Department of Chemical Sciences, University of Padova, Via Marzolo 1, Padova 35131, Italy

**Keywords:** e-PRC, cyclic voltammetry, electrode irradiation, photoinduced electron transfer, perylene diimide, single electron transfer

## Abstract

Recent advancements in electro-photoredox catalysis (e-PRC) and consecutive photoinduced electron transfer (conPET) have pushed the energy limits of conventional photocatalysis. Both methods produce open-shell intermediate catalysts that, upon light absorption, become highly reducing or oxidizing, enabling challenging reactions. Despite their widespread use, the mechanisms of e-PRC and conPET reactions remain debated, in part due to a lack of quantitative data in most studies—particularly single-electron transfer rate constants (*k*_SET_) between excited-state catalysts and substrates. We present a straightforward electrochemical method for determining *k*_SET_ using cyclic voltammetry (CV) under light irradiation, paired with electrochemical simulation. Using inexpensive LEDs and standard potentiostats, we investigated the reactivity of excited-state anions of a perylene diimide dye (PDI), the seminal catalyst of conPET reactions. CV was used to study the photochemical reactivity of both reduced species of PDI, *PDI^•–^ and *PDI^2–^, in the reductive cleavage of carbon–halogen bonds in alkyl and aryl halides. The extreme reactivity of these excited-state anions is confirmed, with quenching rate constants of 10^7^ and 10^10^ M^–1^ s^–1^ for *PDI^•–^ and *PDI^2–^, respectively, consistent with theoretical and experimental data. The voltammetric approach presented here provides a rapid and reliable tool for studying the excited-state reactivity of labile intermediates utilized in e-PRC and conPET systems, including both radical anions and dianions.

## Graphical abstract

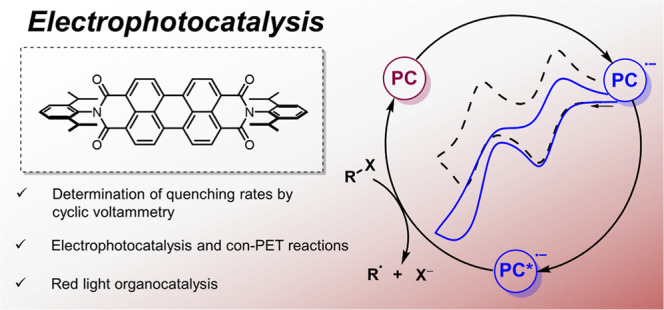

## Introduction

Visible-light photocatalysis is revolutionizing organic synthesis and polymer science by providing access to highly selective reaction pathways. However, photocatalysis is limited by the short lifetime of certain photocatalysts and by the energy attainable after photon absorption. The energy of a blue photon is 2.8 eV, but there are often losses, for example, due to internal conversion or intersystem crossing.^1^ (We estimated 0.5 eV for the photocatalytic system investigated here; see Supporting Information).

In several recent reports, the energy limitations of traditional single-photon catalysis has been overcome by integrating multiple photochemical events within a single catalytic cycle, or by combining photochemical and redox events.^2,3^ This was accomplished via electro-photoredox catalysis (e-PRC, Figure 1a), which merged electrosynthesis with photoredox catalysis to generate potent redox catalysts, or via consecutive photoinduced electron transfer (conPET, Figure 1b), which utilized two sequential photoredox events.

**Figure 1:**
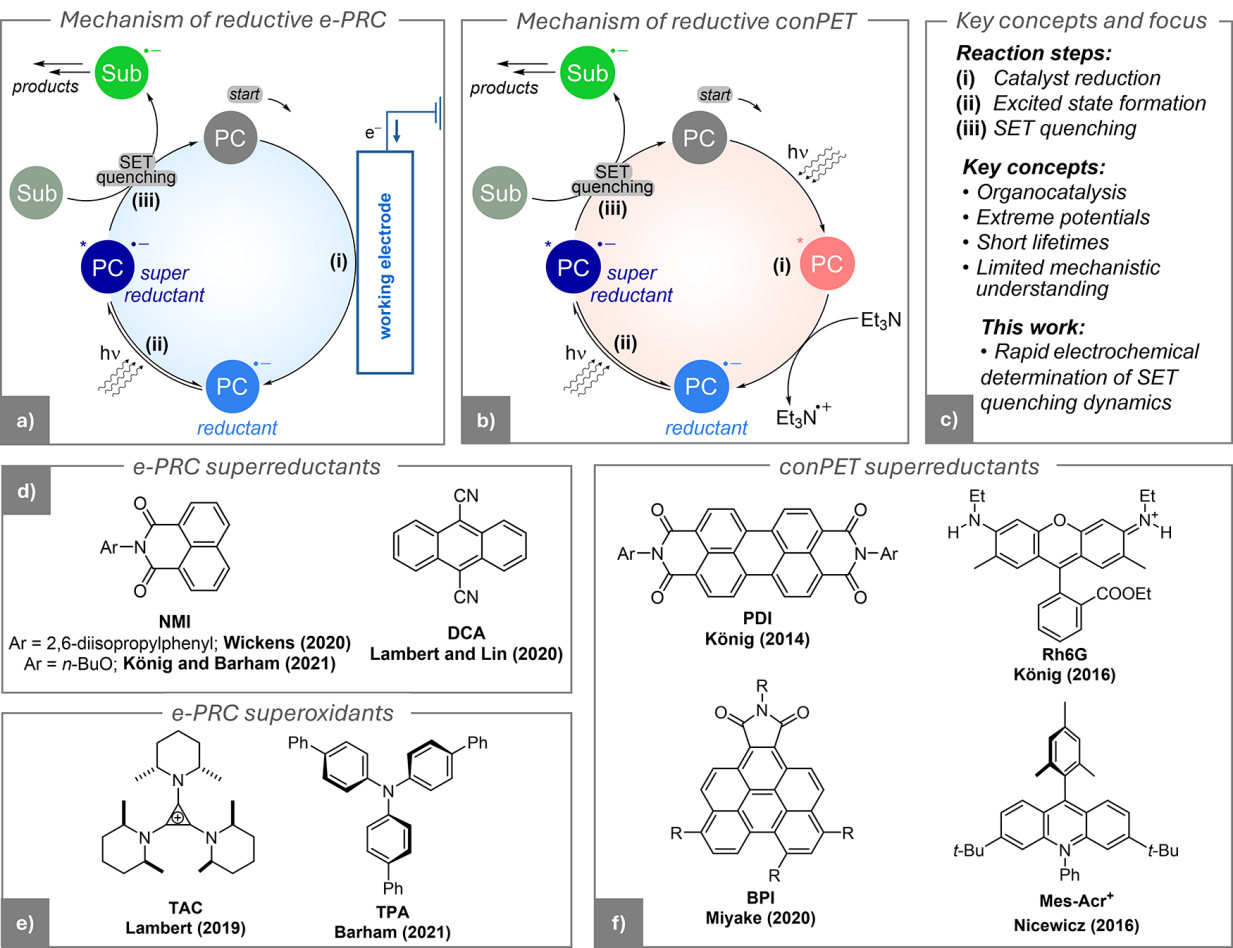
(a) Simplified mechanism of reductive e-PRC. (b) Simplified mechanism of reductive conPET. (c) Key manuscript concepts and focus. (d–f) Structures of organocatalysts used in (d) reductive e-PRC, (e) oxidative e-PRC, and (f) conPET. Ar = 2,6-diisopropylphenyl.

The discovery of e-PRC dates back to the late 1970s,^4–6^ but it has recently experienced a resurgence due to renewed interest in radical chemistry, fueled by the development of both superreductant and superoxidant organocatalysts (see examples in Figure 1d,e).^7^ Instead, conPET was first reported in 2014 with the utilization of the catalyst PDI,^8^ the first of a series of highly reductive photoreductants (examples in Figure 1f).

Figures 1a,b illustrate proposed mechanisms of e-PRC and conPET, focusing on a net substrate reduction reaction (the concepts apply similarly to oxidations).^9^ These simplified mechanisms neglect mechanistic complications due to precomplexation or other phenomena (see discussion below). Both mechanisms in Figure 1a,b are examples of homogeneous photoredox catalysis, and they share three key steps: (i) catalyst reduction, (ii) excited-state formation, and (iii) single-electron transfer (SET) to the substrate. The primary distinction between e-PRC and conPET lies in the catalyst reduction step (i).

In e-PRC, the photocatalyst (PC) is electrochemically reduced to form the electron-primed catalyst PC^•–^, often a radical ion (Figure 1a). In contrast, reductive conPET achieves catalyst reduction photochemically (Figure 1b): the neutral PC absorbs a photon and is reduced to PC^•–^ in the presence of an electron donor, typically triethylamine (Et_3_N). Back-electron transfer between PC^•–^ and the oxidized amine is prevented by deprotonation of amine radical cations.^10^ Most reductive conPET mechanisms can in principle transition to e-PRC by replacing the photochemical reduction step with an electrochemical one. Beyond this initial catalyst reduction step, both mechanisms are proposed to follow the same reaction pathway.

Second, in both mechanisms, the intermediate catalyst PC^•–^ absorbs a photon, forming the excited state *PC^•–^. This is typically an extremely strong photoreductant, with a redox potential between −2 V and −4 V vs SCE, depending on the redox potential of the ground state photocatalyst and wavelength of excitation.^2^

Third, *PC^•–^ reacts with the substrate via a single-electron transfer (SET). The SET quenching is the key step of the process, dictating the rate of substrate reduction via its rate coefficient (*k*_SET_). The reduced substrate is typically trapped with an appropriate radical scavenger to form synthetically relevant products. In this final step, the initial resting state of the catalyst is regenerated, closing the photoredox cycle.

Recently, e-PRC and conPET have been harnessed in various synthetic transformations, including C–H alkylation of heteroarenes,^11^ C–H/N–H coupling,^12^ Birch reductions,^13^ reductive halide coupling,^14,15^ and reduction of phosphinated alcohols^16^ among many others.^17^ Particularly relevant is the ability of certain *PC^•–^ excited states to react with unactivated alkyl and aryl halides as substrates, which is a straightforward methodology to generate radicals via dissociative electron transfer (DET).^15,18^ Some example structures of the utilized organocatalysts are reported in Figure 1d-f.

Despite the many demonstrated applications, the mechanisms of e-PRC and conPET^10,19^ are still debated, partly due to the scarcity of quantitative data on the process (e.g., few *k*_SET_ values available^16^). In the initial reports on e-PRC, the SET step was proposed to occur through a bimolecular reaction between *PC^•–^ (or *PC^•+^) and the substrate.^12,15^ However, this view has been debated due to the extremely short lifetimes (often <1 ns^10,20,21^ ) of most excited-state open-shell intermediates *PC^•–^, which could prevent bimolecular reactivity.^16^ Consequently, precomplexation between ground state catalysts and substrates has been suggested and detected.^16,22,23^ Alternatively, the active photocatalyst has been proposed to arise from decomposition products of the starting catalyst, such as those formed after anion protonation or photodegradation,^24,25^ an issue that impacts both e-PRC and conPET reactions albeit to a different extent.^19^ The ejection of solvated electrons has also been suggested and detected.^10,26,27^

Quantitative studies of e-PRC and conPET are challenging due to the instability of most radical anion intermediates, which are highly reactive even in the dark and prone to protonation, oxidation, or reversion to their neutral state. While cryptands have been used to stabilize and isolate radical anions,^28^ their sensitivity to oxygen complicates studies outside gloveboxes or electrochemical cells.

Due to the short lifetimes of the catalysts’ excited states, kinetic studies rely on pulsed-laser optical techniques such as femtosecond pump–probe and transient absorption spectroscopy (TAS) or time-correlated single photon counting (TCSPC), the latter available only for emissive species. Although these methods are highly sensitive and provide direct information on the lifetime of the absorbing/emissive species, they require specialized equipment and may not be suited to investigate unstable radical ion photocatalysts. Indeed, quantitative studies on e-PRC excited state dynamics are scarce.^21,22,29^

We present a robust electrochemical method to determine *k*_SET_ using cyclic voltammetry (CV) under light irradiation, combined with electrochemical simulation. We investigated via CV the reactivity of the seminal conPET catalyst PDI,^8^ utilized also in e-PRC strategies for the photocatalyzed cleavage of carbon–halogen bonds.^19^ PDI is a versatile electron-primed photocatalyst with two stable reduced states, PDI^•–^ and PDI^2–^. Both species have been successfully used as PC for the photoreductive cleavage of aryl halides (RX), with *PDI^2–^ demonstrating higher reactivity than *PDI^•–^.^29,30^ The electrochemical technique presented here was applied to study the reactivity of both *PDI^•–^ and *PDI^2–^, demonstrating high versatility in determining *k*_SET_ values for short-lived anionic excited states for both radical anions and dianions. No evidence of precomplexation or solvated electron ejection was proposed or observed in these catalytic systems, which simplified our mechanistic interpretation to a bimolecular electron transfer between the excited PDI species and suitable RX substrates, as proposed in the mechanism in Figure 1a.

We demonstrate a clear, reproducible CV current enhancement under light exposure for PDI in the presence of alkyl and aryl halides, contrasting recent studies that reported no noticeable changes in CV upon light exposure.^18^ This current enhancement could be utilized in two ways: (i) to rapidly and qualitatively assess the photoreactivity of reduced PDI species; (ii) through detailed CV analysis coupled with digital simulation, to obtain quantitative information on *k*_SET_ values.

The CV experiments utilized standard potentiostats and low-cost LED lights. Reactive PC^•–^ and PC^2–^ intermediates were transiently generated under anaerobic conditions near the electrode surface, eliminating the need for gloveboxes or other stringent air-free setups. This avoided catalyst decomposition issues associated with direct rapid kinetic measurements (e.g., time-resolved techniques).

CV with electrode illumination provided valuable insights into the full photocatalytic cycle of PDI through simple setups and rapid analysis. Serving as a substitute for—or ideally in combination with—fast optical techniques, CV enabled a rapid and comprehensive understanding of e-PRC and conPET processes.

## Results and Discussion

We first provide a qualitative description of the CV behavior of PDI as an electrophotocatalyst. Next, we outline the model used to determine excited-state quenching rate coefficients (*k*_SET_) for both *PDI^•–^ and *PDI^2–^, drawing inspiration from the seminal work of Lund^6^ and Rusling,^31^ who demonstrated an enhanced current in CV under irradiation, and recent detailed theoretical insights by Costentin.^32^ Finally, we compare *k*_SET_ obtained via CV with theoretical and independently measured values.

## Qualitative Description of the CV of PDI under Electrophotocatalytic Conditions

In the dark, PDI is consecutively reduced to its open-shell radical anion and closed-shell dianion, giving rise to two successive quasi-reversible waves on a glassy carbon (GC) disk electrode (dashed blue line in Figure 2a).

1 $$PDI+e⇄PDI^{•-}$$

2 $$PDI^{•-}+e⇄PDI^{2-}$$

**Figure 2:**
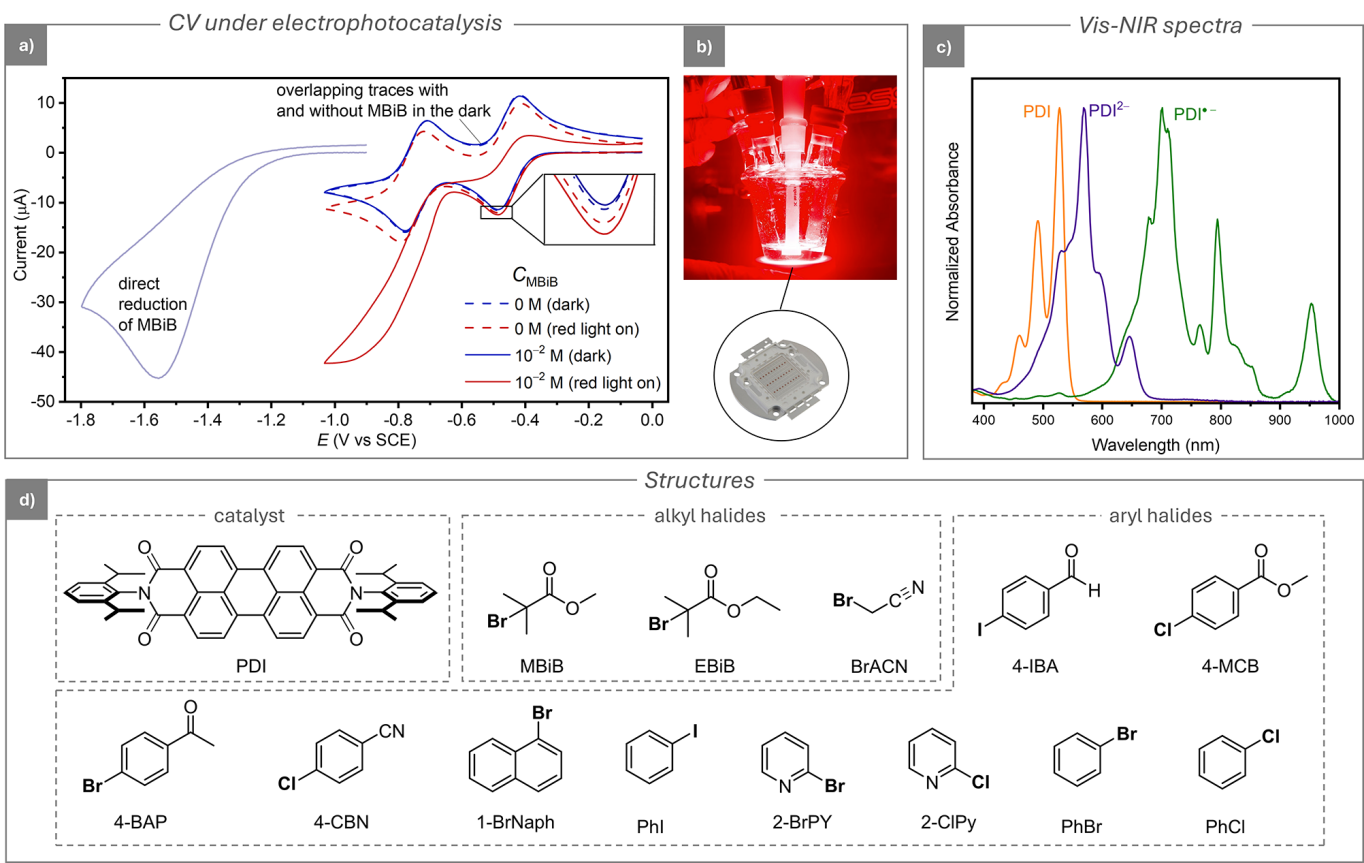
(a) CVs of 10^–3^ M PDI in DMF + 0.1 M *n*-Bu_4_NBF_4_ on a GC electrode (*A* = 7.07 mm^2^) in the absence (dashed lines) and in the presence (solid lines) of 10^–2^ M MBiB. CVs were recorded in the dark (blue lines) or under 630 nm red light irradiation (red lines). *T* = 25 °C, *v* = 0.1 V s^–1^. Light blue: CV of 2 mM PDI at 0.1 V s^–1^. (b) Digital picture of the LED and of the CV setup under irradiation. (c) Vis-NIR spectra of 2.5 × 10^–4^ M PDI and its reduced species in DMF +0.1 M *n*-Bu_4_NBF_4_. The reduced species were obtained by electrolysis in a spectroelectrochemistry cell using a Pt electrode: PDI^•–^ was generated at –0.55 V vs SCE, and PDI^2–^ was generated at –1.2 V vs SCE. (d) Molecular structures of catalyst (PDI) and the organic halide substrates (RX) investigated in this work.

The standard reduction potentials of the two redox couples, calculated from their half-wave potentials, are $E_{PDI/{PDI}^{•-}}^{o}$ = −0.410 V vs SCE and $E_{{PDI}^{•-}/{PDI}^{2-}}^{o}$ = −0.703 V vs SCE. These modest values indicate that both PDI^•–^ and PDI^2–^ are relatively weak reducing agents in the absence of light irradiation. Consistent with their low reactivity as electrocatalysts in the dark, adding the alkyl halide substrate methyl α-bromoisobutyrate (MBiB, *E*_MBiB_^o^ = −0.52)^33^ to the PDI solution caused no change in the CV response (solid blue line in Figure 2a). The overlap of the curves with and without the substrate confirms that no reaction occurred in the dark during the CV time scale. The direct reduction of MBiB appears as an irreversible wave at a potential significantly more negative than that of the catalyst reduction (light blue line in Figure 2a). The wave is also much more negative than the value *E*_MBiB_^o^ = −0.52, a shift that reflects substantial overpotential associated with its dissociative electron transfer.^34^

The Vis-NIR spectra of PDI, PDI^•–^, and PDI^2–^ are shown in Figure 2c. The spectra of both reduced species are significantly red-shifted compared to that of neutral PDI; therefore, PDI^•–^ and PDI^2–^ can be selectively irradiated with a 630 nm LED without affecting the neutral PDI.

Next, we investigated the effect of light on the CV response, without RX, under electrode irradiation with a commercial LED array (30 W, 630 ± 5 nm, at ca. 1 cm from the electrode surface, see setup details in the Supporting Information). A digital picture of the setup is presented in Figure 2b. Irradiation of the electrode surface during the CV scan led to the formation of *PDI^•–^ and *PDI^2–^. Light irradiation caused an increase in the cathodic currents and a decrease in the anodic ones (dashed red line in Figure 2a), suggesting enhanced mass transport due to local heating near the electrode surface, triggering local convection, and, therefore, a change of mass transport regime from pure diffusion to mixed diffusion-convection.

We then examined the complete electrophotocatalytic cycle, under irradiation and in the presence of the RX substrate. The CV response showed a dramatic change, with a catalytic current appearing (solid red line in Figure 2a). The cathodic current of the first wave (PDI/PDI^•–^) slightly increased, while the current of the second wave (PDI^•–^/PDI^2–^) increased significantly, with the peak becoming irreversible. These changes clearly indicated electrophotocatalysis occurring under irradiation near the electrode surface, observed as an enhanced cathodic peak accompanied by a decrease or disappearance of its anodic partner.

The increase in cathodic current of the two CV waves aligns with the reported reactivity of the PDI^•–^ and PDI^2–^ excited states,^29,30^ with the more reducing and longer-lived *PDI^2–^ causing a much larger change. Due to the small response in CV, the weaker reactivity between *PDI^•–^ and MBiB was also confirmed by constant potential electrolysis under irradiation, which showed the near quantitative reductive cleavage of MBiB after 24 h (Figure S30–S31). Furthermore, MBiB was recently used to generate a small radical stream that sustained a polymerization via conPET mediated by *PDI^•–^.^35^ Overall, these findings confirmed that CV under electrode illumination is a viable and rapid method to study the photoredox reactivity of *PDI^•–^ and *PDI^2–^ toward the reduction of the alkyl halide MBiB.

Additionally, the CV was sensitive to the light wavelength, with 730 nm light showing much lower catalytic current for the PDI^•–^/PDI^2–^ couple, due to the low absorption of PDI^2–^ at this wavelength (Figure S28). The first PDI/PDI^•–^ wave also responded to the light wavelength proportionally to the absorption coefficient of PDI^•–^ (Figure S29).

Encouraged by these results, we next developed a quantitative approach to use CV under e-PRC conditions for determining the quenching rate coefficient *k*_SET_ of *PDI^•–^ with various alkyl halides. The photochemical reactivity of the more reactive *PDI^2–^ with recalcitrant aryl halides will be addressed later.

## CV as a Quantitative Tool to Determine Quenching Rate Coefficients for *PDI^•–^

Our approach relies on digital simulation of the CV response under e-PRC conditions. CV simulation, an established tool for obtaining robust kinetic data,^36–41^ has been applied to the detailed mechanism in Figure 3a. This cycle involves DET to an organic halide (RX).^34,42^ Compared to traditional and established electrocatalysis in the dark,^43^ the key additional steps are the formation and decay of the excited-state *PDI^•–^.

**Figure 3:**
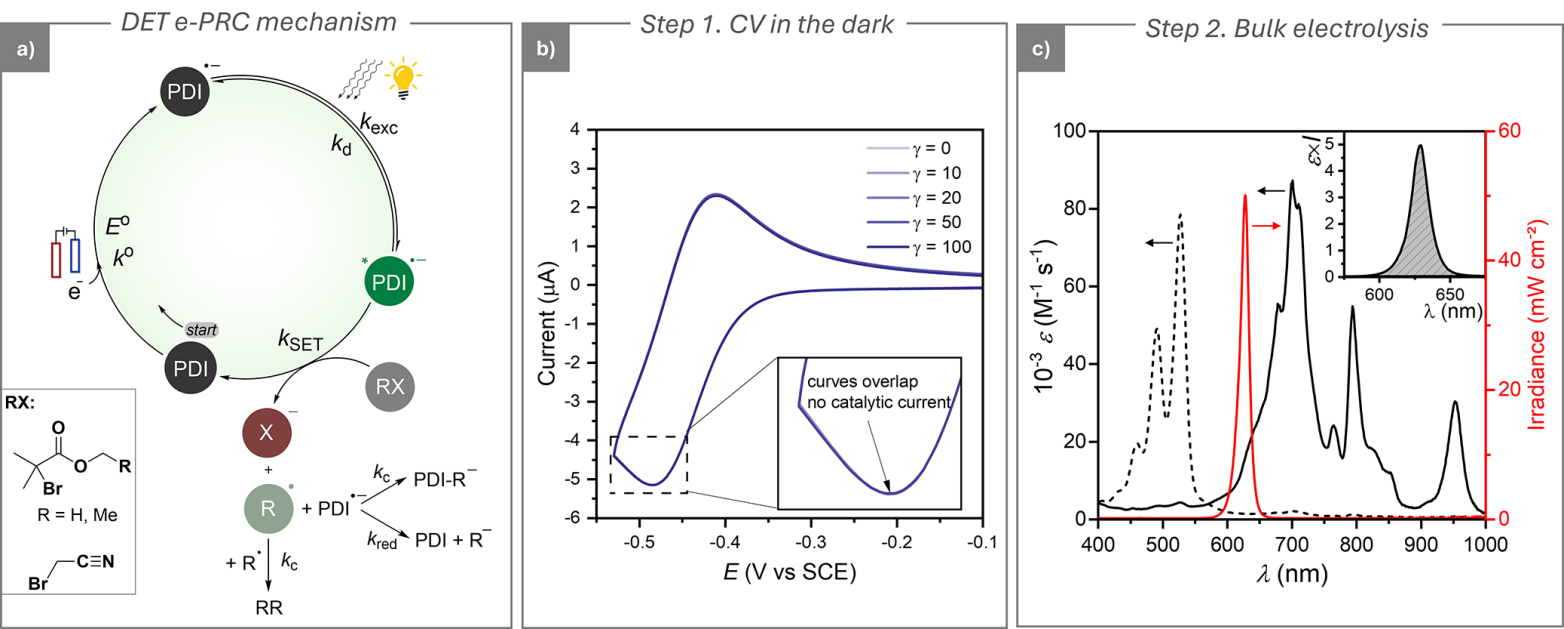
(a) Proposed electrophotocatalytic mechanism for the DET of alkyl halides. (b) CVs of 10^–3^ M PDI in DMF + 0.1 M *n*-Bu_4_NBF_4_ on a GC electrode (*A* = 7.07 mm^2^) in the absence and in the presence of MBiB at different concentrations, labeled on the curves (γ = *C*_MBiB_/*C*_PDI_). *T* = 25 °C, *v* = 0.02 V s^–1^. (c) Vis-NIR absorption spectra of PDI (dashed black line) and PDI^•–^ (solid black line) in DMF + 0.1 M *n*-Bu_4_NBF_4_. PDI^•–^ was generated in a degassed spectroelectrochemical cell (path length *l* = 0.5 mm) on a Pt mesh electrode at −0.55 V vs SCE. The solid red line represents the absolute irradiance of the 630 nm LED, measured using an Avantes AvaSpec spectrophotometer. Inset: plot of ε*I* vs λ used for calculating the *k*_exc_ value via eq 6.

The first step of the process (Figure 3a) is PDI^•–^ generation at the electrode, followed by light absorption with the formation of the excited state *PDI^•–^ with a rate coefficient *k*_exc_. *PDI^•–^ can either decay back to its ground state with a rate coefficient *k*_d_ or react with RX via SET with a rate coefficient *k*_SET_. Following SET, neutral PDI is regenerated, closing the catalytic cycle. For alkyl halides, SET triggers concerted C–X bond cleavage.^44^ The concerted cleavage of alkyl halides requires substantial overpotential to proceed at appreciable rates, but provides rapid evolution to products (R^•^ and X^–^) that reduces the impact of back electron transfer reactions. The resulting radical R^•^, in the absence of a radical trap, further reacts with electrogenerated PDI^•–^ via two competing reactions, coupling (eq 3) and reduction (eq 4), which have been widely documented in electrocatalytic cycles^34,45,46^

3 $$R^{•}+PDI^{•-}\overset{k_{c}}{\to }R‐PDI^{-}$$

4 $$R^{•}+PDI^{•-}\overset{k_{red}}{\to }R^{-}+PDI$$

The R^–^ anion is a strong base that is quickly protonated by adventitious water or by the solvent. Biradical coupling of organic radicals typically proceeds with high rate coefficients, *k*_c_ = 10^9^ M^–1^ s^–1^.^47–50^ Conversely, the value of *k*_red_ strongly depends on the reducing power of PDI^•–^. Therefore, competition between *k*_c_ and *k*_red_ is determined by the driving force of the reduction reaction, which is proportional to the difference between the values of $E_{PDI/{PDI}^{•-}}^{o}$ and $E_{R^{•}/R^{-}}^{o}$ . Note that under typical homogeneous redox catalysis conditions these radical reactions occur near the electrode surface, where concentrations of reactive species (PDI^•–^ and R^•^) are high, and thus conditions may differ from those of preparative electrophotochemical reactions occurring under more dilute excited-state concentrations. Reaction between *PDI^•–^ and R^•^ was neglected because both species are present at extremely low concentrations (see SI, Section S7, for details; several other possible reactions were considered but found to have no effect on the CV response).

Determination of *k*_SET_ via a CV simulation method required the independent measurement or estimation of various thermodynamic and kinetic parameters of the catalytic cycle (labeled in Figure 3a). To this end, a step-by-step procedure was followed:^32^ (i) CV in the dark, (ii) spectroelectrochemistry, (iii) CV under irradiation without substrate, (iv) CV under irradiation with substrate, and (v) digital simulation of CV with fitting of experimental voltammograms.

### Step 1. CV in the Dark

This step evaluated key electrochemical parameters of the PDI catalyst in DMF + 0.1 M *n*-Bu_4_NBF_4_ at 25 °C (Table 1). The cathodic scan was limited to −0.53 V vs SCE to isolate the PDI/PDI^•–^ redox couple (Figure 3b). The standard electron transfer rate constant, determined via the Nicholson method, was *k* ^0^ = 0.019 cm s^–1^ (Figure S3), notably lower than typical values for aromatic molecules—suggesting significant inner reorganization. The diffusion coefficient was *D* = 3.3 × 10^–6^ cm^2^ s^–1^ (Randles–Ševčík equation, Figure S4). Addition of excess MBiB had no effect on the CV in the dark (Figure 3b), confirming that reactivity between PDI^•–^ and RX requires light. The absence of change even with 100-fold excess of RX also ruled out potential preassociation between PDI^•–^ and RX, as formation even of a weak complex would result in an anodic shift of the voltammogram.^51^ Supporting the CV measurements, no significant changes in the UV-Vis-NIR spectrum of PDI^•–^ were detected in the presence of RX (Figure S26a).

**Table 1:** Electrochemical Properties of PDI in the Dark and Under 630 nm Light Irradiation

	PDI/PDI^•–^	PDI^•–^/PDI^2–^
$E_{PC/{PC}^{-}}^{o}$ /V vs SCE	–0.410	–0.703
*k* ^0^ (PC/PC^–^)^a^/cm s^–1^	0.019	0.020
*E*_0–0_ ^b^/eV	1.46^c^	1.90
$E_{PC/{*PC}^{-}}^{o}$ /V vs SCE	–1.87^c^	–2.60^d^
*D* ^e^/cm^2^ s^–1^	3.3 × 10^–6^	
*D* (under light)^f^/cm^2^ s^–1^	(4.8 ± 0.4) × 10^–6^	
δ (under light)^f^/μm	73 ± 2	73 ± 3

a Heterogenous rate constant of electron transfer obtained using the Nicholson method (Supporting Information, Section S3.1).

b Excitation energy of the photocatalyst, calculated as *E*_0–0_ = *hc*/λ_int_, with λ_int_ the intersection of the normalized absorption and emission spectra (Figure 7a).

c From ref 29.

d Calculated as $E_{PDI^{•-}/*PDI^{2-}}^{o}=E_{PDI^{•-}/PDI^{2-}}^{o}-E_{0‐0}$ .

e Diffusion coefficient of PDI obtained using the Randles–Ševčik equation (Supporting Information, Section S3.2).

f *D* of PDI determined using the CV simulation software DigiElch 8. δ represents the maximum diffusion layer thickness under 30 W 60 nm irradiation.

### Step 2. Spectroelectrochemistry

PDI^•–^ was generated in a degassed spectroelectrochemical cell to record its absorption spectrum (Figure 3c). From this, a 630 nm LED was chosen for selective irradiation of PDI^•–^ because its absorption spectrum is red-shifted compared to that of PDI, which shows negligible absorbance at this wavelength, while PDI^•–^ has ε ≈ 10^4^ M^–1^ cm^–1^. Tests indicated that a value of ε ≤ 2 × 10^4^ M^–1^ cm^–1^ at a 10^–3^ M catalyst concentration was optimal for quantitative *k*_SET_ measurements (see further discussion on the setup in the Supporting Information, Section S3.3). The lifetime of *PDI^•–^, reported as τ_0_ = 160 ps in the literature,^29^ yielded *k*_d_ = 1/τ_0_ = 6.25 × 10^9^ s^–1^. Relevant photophysical properties of the catalytic system are summarized in Table 2.

**Table 2:** Photophysical Properties of the PDI Catalyst Family

	PDI	PDI^•–^	PDI^2–^
ε (630 nm)/M^–1^ cm^–1^	≈0	16,620	13,140
τ_0_/ns	≈4^a^	0.160^b^	5.88^c^
*k*_d_ ^d^/s^–1^		6.25 × 10^9^	1.70 × 10^8^
*k*_exc_ (30 W, 630 nm)^e^/s^–1^		74	86

a Excited-state lifetimes from refs 52 and 53 .

b From ref 29.

c Determined from TCSPC measurements (Figure 7b).

d Decay rate constant calculated as 1/τ_0_.

e Rate coefficient for formation of the excited state, calculated from eq 6. Note: *k*_exc_ depends on irradiation source. An estimate value of *k*_exc_ can be obtained by direct multiplication of ε­(630 nm) with the total lamp irradiance *I*.

### Step 3. CV under Irradiation without Substrate

This step assesses the effect of light-induced local heating on the CV (with the cell bulk temperature thermostated at 25 °C). Under electrode irradiation at 630 nm, the CV of PDI deviated from its traditional reversible shape, showing increased cathodic peak and decreased anodic one (Figure 4a). This behavior indicated enhanced mass transport at the electrode due to local heating, primarily resulting from light absorption by both PDI^•–^ and the electrode material, followed by nonradiative decay. The localized heating enhanced the diffusion coefficients and induced convection, which influenced the mass transfer profiles. To quantify the altered profiles, we performed digital simulations of the CVs with the DigiElch 8 software.

**Figure 4:**
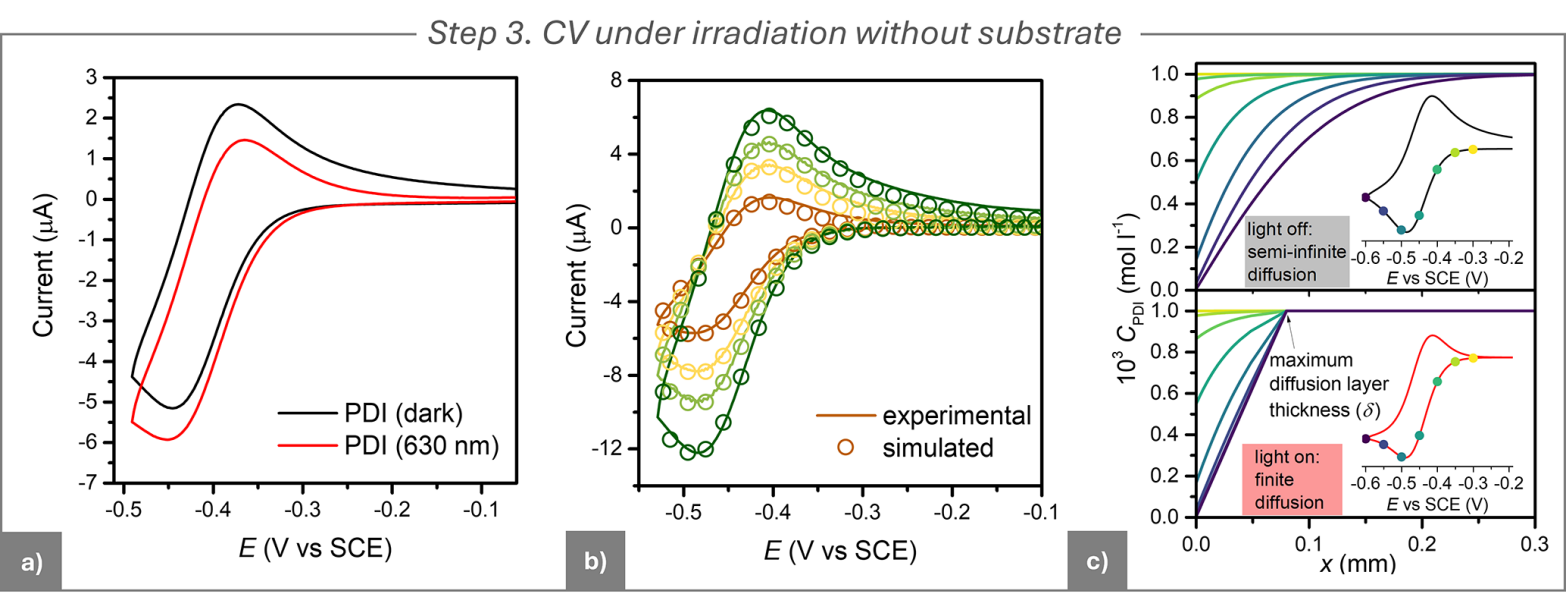
(a) CVs of 10^–3^ M PDI in DMF + 0.1 M *n*-Bu_4_NBF_4_, recorded using a GC electrode (*A* = 7.07 mm^2^) at *T* = 25 °C. Scans were performed in the dark and under irradiation with a 630 nm light. (b) Experimental (solid line) and simulated (circles) CVs of 10^–3^ M PDI under irradiation at different scan rates ranging from 0.02 to 0.1 V s^–1^. (c) Simulated PDI concentration profiles near the electrode surface during the CV of 10^–3^ M PDI. Simulations assume: (top) semi-infinite diffusion conditions (bottom) finite diffusion conditions with a diffusion layer thickness δ = 73 μm. The concentration profiles are shown for potentials indicated by dots on the corresponding simulated CV curves.

In the CV simulation model, enhanced mass transport was captured by transitioning from a semi-infinite diffusion profile, typical for CV in the dark, to finite diffusion,^32^ which accounts for light induced convection. Finite diffusion, also used to describe mass transport under stirring, is characterized by a maximum diffusion layer thickness, δ.^54^ By fitting experimental CV traces under light irradiation at varying scan rates, we determined δ = 73 ± 2 μm. Figure 4b shows excellent agreement between the fitted and experimental CV data, confirming that planar finite diffusion with a constant δ value could accurately model the mass transport across all investigated scan rates. Therefore, the enhanced current in the absence of RX is not a true photocurrent, as no new electrode reactions are triggered; only mass transport increases, allowing more PDI molecules to reach and then leave the electrode surface.

Figure 4c compares “dark” and “light” CVs by plotting PDI concentration profiles versus distance from the electrode (*x*). Under illumination, enhanced mass transport restored the bulk PDI concentration at *x* = δ (73 μm), the maximum diffusion layer thickness in the finite diffusion model.

No appreciable changes in the values of $E_{PDI/{PDI}^{•-}}^{o}$ and *k°* were detected under irradiation. CV simulation (Figure 4b) yielded *D =* (4.8 ± 0.4) × 10^–6^ cm^2^ s^–1^, about 50% higher than in the dark, consistent with diminished viscosity from local heating.

The temperature at the diffusion layer under irradiation was estimated by building a calibration curve for the diffusion coefficient of PDI versus temperature (in the dark, Figure S5a). Under irradiation, a surface temperature of 49 ± 4 °C was determined, indicating a ∼24 °C local *T* increase due to light exposure.

### Step 4. CV under Irradiation with Substrate

After studying the effect of light on CV, we examined the full e-PRC cycle with MBiB as the substrate. In the dark, PDI^•–^ showed poor reducing power ( $E_{PDI/{PDI}^{•-}}^{o}$ = −0.410 V vs SCE). Under irradiation, its reducing ability improved significantly, reaching EPDI/PDI*•−o = −1.87 V vs SCE.^4^

Upon substrate addition under light, the cathodic peak increased while the anodic peak decreased (Figure 5a), indicating the occurrence of electrophotocatalysis consistent with the mechanism in Figure 3a. The catalytic current resulted from the reduction of MBiB by *PDI^•–^, regenerating neutral PDI, which was then reduced again at the electrode to start further catalytic cycles.

**Figure 5:**
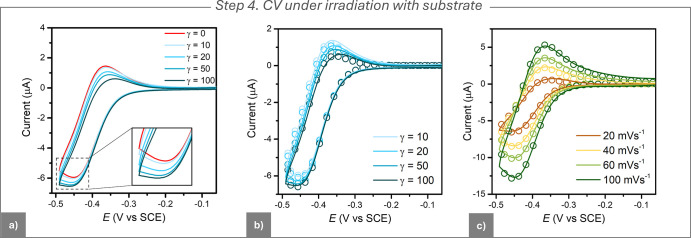
CVs of 10^–3^ M PDI under irradiation with 630 nm light. (a) Experimental CVs recorded with increasing concentrations of MBiB (γ = *C*_RX_/*C*_PDI_), at a scan rate of 0.02 V s^–1^. (b) Comparison between experimental (solid lines) and simulated CVs (circles) at various MBiB concentrations at *v* = 0.02 V s^–1^. (c) Comparison between experimental (solid lines) and simulated (circles) CVs recorded at a fixed concentration ratio (γ = 100) and various scan rates. General conditions for all panels: GC electrode (*A* = 7.07 mm^2^); DMF + 0.1 M *n*-Bu_4_NBF_4_; *T* = 25 °C.

The electrophotocatalysis was relatively slow, as indicated by the small changes in the CV pattern at high substrate concentrations (high γ = *C*_RX_/*C*_PDI_). This inefficient catalysis is consistent with the mild excited-state potential and the short lifetime of *PDI^•–^.^29^ The current increase was not due to local heating only, as no catalytic currents were observed in the dark at 50 °C (Figure S5b). Therefore, it was an electrophotocatalytic response.

### Step 5. CV Simulation

Simulation of CVs to determine *k*_SET_ required also determination of the rate coefficient for *PDI^•–^ excited state formation, *k*_exc_. *PDI^•–^ is formed via a first-order reaction driven by light absorption.

5 PDI•−→kexcPDI•−*

The value of *k*_exc_ was calculated from the following relationship^32,55^

6 $$k_{exc}=\int_{550\;nm}^{700\;nm}\varepsilon \left(\lambda \right)I\left(\lambda \right)d\lambda$$

where ε­(λ) is the molar absorption coefficient of *PDI^•–^ and *I*(λ) is the lamp irradiance at the electrode surface. Figure 3c shows *I*(λ), with the inset figure illustrating the overlap integral in eq 6. For PDI^•–^ irradiated by a 30 W 630 nm lamp, *k*_exc_ = 74 s^–1^ was determined. Detailed calculations (see Supporting Information, Section S3.3) revealed that the value of *k*_exc_ could be considered approximately constant during the CV scan.

CVs at varying γ and scan rates were digitally fitted to the mechanism in Figure 3a, with *k*_SET_ as the sole variable (see Table 3 and the Supporting Information, Section S7 for a full list of the simulated parameters). The fitting showed excellent agreement between experimental and simulated CVs, yielding log *k*_SET_ = 7.9 (see Figure 5b for examples of CV fitting at different γ values and Figure 5c for various scan rates).

**Table 3:** Kinetic Data for the Electrophotocatalytic SET between PDI Reduced States and RX Substrates

entry	catalyst	RX	*E*_RX_^0^ (V vs SCE)	10^5^ *D*_RX_ ^a^ (cm^2^ s^–1^)	log *k*_fr_ ^b^	log *k*_SET_ ^c^	log *k*_red_ ^d^	log *k*_q_ ^e^	γ^f^
1	*PDI^•–^	MBiB	–0.52^g^	1.08		7.9	n.d.	n.d.	10, 20, 50, 100
2	*PDI^•–^	EBiB	–0.46^g^	1.04		7.8	n.d.	n.d.	10, 20, 50, 100
3	*PDI^•–^	BrACN	–0.49^g^	1.24		8.0	n.d.	n.d.	10, 20, 50, 100
4	*PDI^•–^	4-IBA	–1.68^h^	0.93	2.3^i^	>7.3	n.d.	9.5^h^	20, 50, 100, 150, 250
5	*PDI^•–^	4-MCB	–2.02^j^	1.10	7.1	≤7	n.d.	n.d.	0.25–100
6	*PDI^2–^	4-MCB	–2.02^j^	1.10	7.1	9.7	8.9	9.8	0.25, 0.5, 0.75, 1, 3
7	*PDI^2–^	4-BAP	–1.84^j^	1.02	7.5	10.3	8.8	n.d.	0.5, 0.7, 1
8	*PDI^2–^	4-CBN	–2.03^j^	1.19	8.7	9.9	8.9	n.d.	0.25, 0.5, 0.75, 1, 2
9	*PDI^2–^	1-BrNaph	–2.17^j^	1.00	9.0	9.8	8.4	9.7	0.25, 0.5, 0.75, 1, 1.5
10	*PDI^2–^	PhI	–2.24^k^	1.01	>10.3	8.7	8.4	9.2	3.5, 6.5, 10 15, 30
11	*PDI^2–^	2-BrPy	–2.26^j^	1.14	9.5	9.3	8.2	n.d.	0.5, 1, 1.5, 2
12	*PDI^2–^	2-ClPy	–2.37^j^	1.26	9.6	8.4	8.3	n.d.	1, 1.5, 2.5, 4, 6, 10
13	*PDI^2–^	PhBr	–2.43^j^	1.14	>10.3	6.9	8.3	7.1	20, 50, 100, 150
14	*PDI^2–^	PhCl	–2.76^h^	1.26	10.3	≤7	n.d.	≤7	20, 50

a Diffusion coefficients of RX calculated with the empirical method reported by Valencia and González.^56^

b Radical anion fragmentation rate constants from the literature.^57,58^

c Bimolecular SET rate constant obtained from CV via digital simulation (DigiElch8) of the mechanism in Figure 3a (for *PDI^•–^, with *k* ^0^ = 0.019 cm s^–1^, *k*_exc_ = 74 s^–1^, *k*_d_ = 6.3 × 10^9^ s^–1^) or Figure 6a (for *PDI^2–^, with *k* ^0^ = 0.020 cm s^–1^, *k*_exc_ = 87 s^–1^, *k*_d_ = 1.7 × 10^8^ s^–1^, *k*_c_ = 10^9^ M^–1^ s^–1^). Sensitivity analysis suggested that the error on *k*_SET_ is within 0.3 log units. Full details in Supporting Information, Section S7. No reactivity between PhCl and *PDI^2–,^ was observed (Supporting Information, Section S8.11).

d Rate constant of the reduction of R^•^ by PDI^2–^ (refer to mechanisms in Figure 6a). Determined via CV simulation. n.d. = not determined.

e Quenching rate constants determined from TCSPC or TAS experiments.

f Excess factors γ = *C*_RX_/*C*_PDI_; all the experiments were performed with *C*_PDI_ = 10^–3^ M.

g From ref 59.

h From ref 29.

i Value for 4-chlorobenzaldehyde.^57^

j From ref 57.

k From ref 60.

Besides solution-phase electron transfer (*k*_SET_) and photophysical processes, catalytic efficiency depends on the fate of the electrode-generated PDI^•–^. It can couple with the alkyl radical R^•^ (eq 3) or reduce it (eq 4). Due to low activity and currents in *PDI^•–^-mediated reductions, the fitting procedure was insensitive to the values of *k*_red_ or *k*_c_, preventing their accurate determination.

Using the same CV simulation method, *k*_SET_ was measured for *PDI^•–^ reacting with two other activated alkyl halides (entries 1–3 in Table 3). Ethyl α-bromoisobutyrate (EBiB) reacted slightly slower than MBiB, likely due to their structural similarity. The log *k*_SET_ values for MBiB and EBiB aligned with expectations based on their electronic properties: the ethyl group in EBiB is slightly more electron-donating, making it marginally harder to reduce than MBiB, which is consistent with its lower *k*_SET_ value. Bromoacetonitrile (BrAN) exhibited the highest reactivity, with a log *k*_SET_ value surpassing those of both MBiB and EBiB. This is consistent with the known higher efficiency of BrAN as an initiator in atom transfer radical polymerization.^37^

It is important to note that with all these substrates catalytic current appears despite the low lifetime of *PDI^•–^. During the time scale of the CV experiment multiple excitation events occur, but only a few lead to productive bimolecular quenching of RX; most undergo thermal decay with *k*_d_ = 6.25 × 10^9^ s^–1^. The quantum yield for SET from the excited state can be estimated from

7 $$\varphi =\frac{v_{SET}}{v_{SET}+v_{d}}$$

where *v*_SET_ and *v*_d_ are the rate of SET and nonradiative decay. After considering that *v*_d_ ≫ *v*_SET_ and substituting the rate laws, we obtain

8 $$\varphi ≅\frac{k_{SET}\left[RX\right]}{k_{d}}=k_{SET}\left[RX\right]\tau_{0}$$

Assuming [RX] is in large excess and remains constant, for [RX] = 0.1 M we find *φ* ≅ 10^–3^. Thus, only 1 in 1000 excited states productively engages with RX, which is in line with the low observed catalytic currents.

Besides alkyl halides, the CV of PDI/PDI^•–^ was examined with two aryl halides previously shown by König et al. to undergo reductive cleavage with *PDI^•–^ via conPET under blue light (entries 3, 4 in Table 3): 4-iodobenzaldehyde (4-IBA) and methyl 4-chlorobenzoate (4-MCB).^8^ CV measurements revealed a small but clear light-dependent catalytic current for 4-IBA (Figure S12c). In this case, fitting yielded only a lower bound value of log *k*_SET_ > 7.3 due to slow bond cleavage of the C–I bond, which promotes back electron transfer reactions (see further discussion below). Consistently, Schanze et al. measured log *k*_SET_ = 9.5 via TAS.^29^

No CV reactivity was observed with 4-MCB, indicating that SET was much slower than the thermal decay of *PDI^•–^. Although *PDI^•–^ can reduce bulk 4-MCB on a time scale of hours,^8^ this is not captured by the CV method, which probes only a few seconds. Thus, log *k*_SET_ ≤ 7 M^–1^ s^–1^ was assigned to the *PDI^•–^/4-MCB system, underscoring the limitation of CV for such cases of low *k*_SET_ values and short catalyst lifetimes.

## CV as a Quantitative Tool to Determine Quenching Rate Coefficients for *PDI^2–^

Extending the potential sweep to −0.85 V vs SCE enabled the generation of both PDI^•–^ and PDI^2–^ for photocatalytic studies. A reactivity test with the less reactive aryl halide, 4-MCB, showed that, unlike *PDI^•–^, the excited dianion *PDI^2–^ could engage this aryl chloride.

The determination of *k*_SET_ for *PDI^2–^ and 4-MCB followed the same procedure used for *PDI^•–^, with minor adjustments. The relevant mechanism (Figure 6a) involves consecutive reductions of PDI to PDI^•–^ and PDI^2–^. Due to the inactivity of *PDI^•–^, only the photochemistry of *PDI^2–^ was considered for 4-MCB photoreduction. *PDI^2–^ induced reductive cleavage of 4-MCB in a stepwise process, generating RX^•–^, which dissociated into R^•^ and X^–^ with a fragmentation rate constant *k*_fr_ = 1.3 × 10^7^ s^–1^,^57^ making the reaction irreversible (see Supporting Information, Section S7.4. Fragmentation slower than 10^7^ s^–1^ may result in significant back electron transfer between RX^•–^ and PDI^•–^). The resulting radical R^•^ can be reduced by PDI^2–^ to form an R^–^ (with rate constant *k*_red_), which is subsequently protonated by the solvent or adventitious water. Alternatively, R^•^ can undergo biradical coupling with PDI^•–^ or with itself (with rate constant *k*_c_ = 10^9^ M^–1^ s^–1^). Reduction of the aromatic radical by PDI^•–^ was neglected, as its reduction potential ( $E_{PDI/{PDI}^{•-}}^{o}$ = −0.410 V vs SCE) is insufficient; aromatic radicals are reduced at much more negative potentials, e.g., ∼−0.9 V vs SCE for the phenyl radical.^61,62^

**Figure 6:**
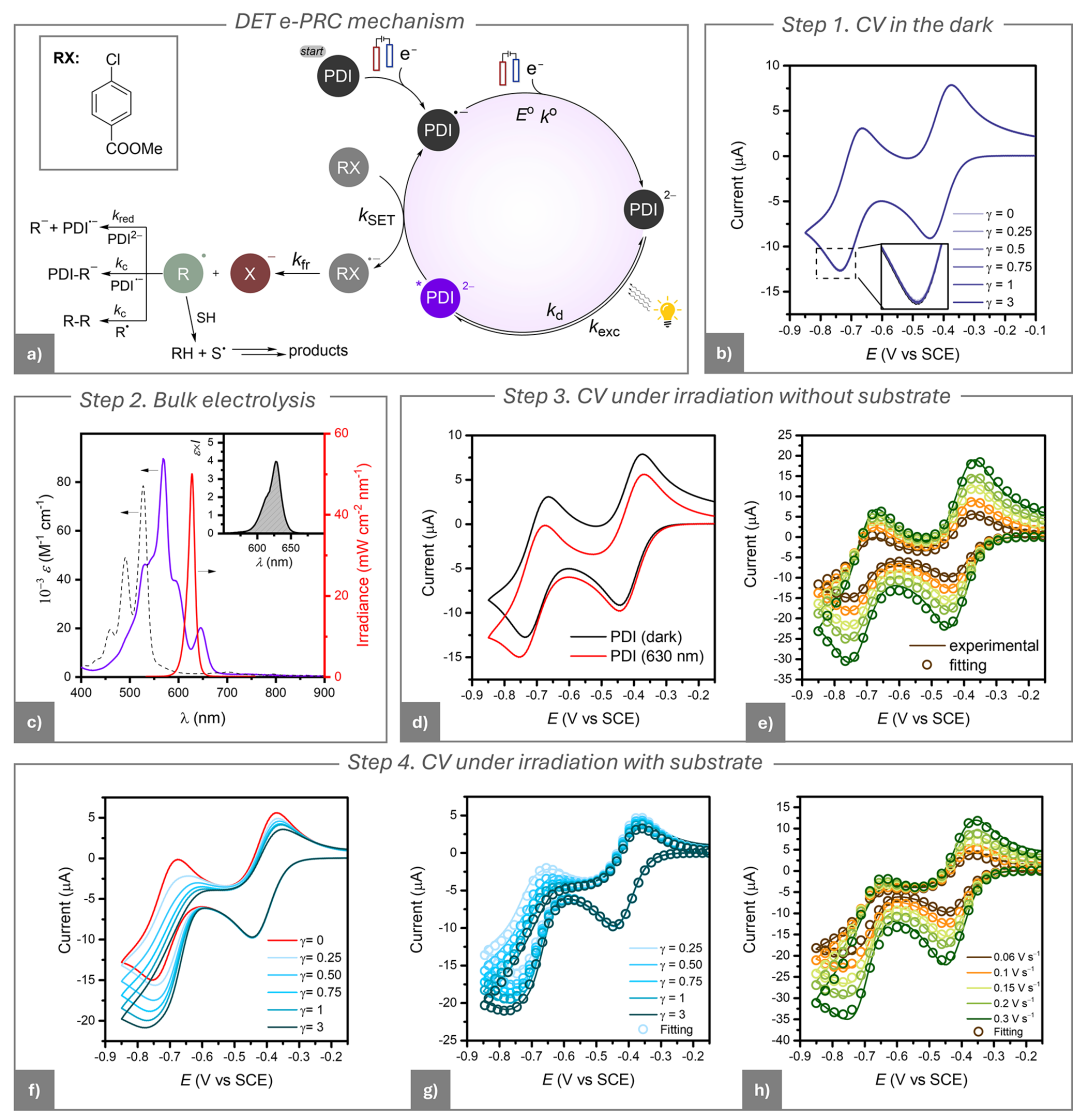
(a) Proposed electrophotocatalytic mechanism for DET of aryl halides with *PDI^2–^. Note that the *k*_c_ value encompasses all radical coupling pathways, between PDI^•–^, R^•^, or S^•^. (b) CVs of 10^–3^ M PDI recorded in the dark in the absence and in the presence of 4-MCB at various concentrations, labeled on the curves (γ = *C*_4‑MCB_/*C*_PDI_); Scan rate *v* = 0.06 V s^–1^. (c) Vis-NIR absorption spectra of PDI (dashed black line) and electrochemically generated PDI^2–^ (solid purple line). PDI^2–^ was generated in a degassed spectroelectrochemical cell (*l* = 0.5 mm) using a Pt-mesh electrode held at −1.2 V vs SCE. The solid red line represents the absolute irradiance of the 630 nm LED. The inset shows the ε*I* vs λ curve utilized for the calculation of *k*_exc_ of PDI^2–^ via eq 6. (d) CVs of 10^–3^ M PDI in the dark and under 630 nm light irradiation at *v* = 0.06 V s^–1^. (e) Comparison between experimental (solid lines) and simulated (circles) CVs of 10^–3^ M PDI, recorded under irradiation with 630 nm light at various scan rates from 0.02 to 0.1 V s^–1^. (f) CVs of 10^–3^ M PDI under irradiation recorded in the presence of varying amounts of 4-MCB, labeled on the curves (γ = *C*_4‑MCB_/*C*_PDI_). Scan rate *v* = 0.06 V s^–1^. (g,h) Comparison of experimental (solid lines) and simulated (circles) CVs of 10^–3^ M PDI + 4-MCB under irradiation: (g) at a fixed scan rate *v* = 0.06 V s^–1^ and various concentration ratios γ; (h) at a fixed γ = 1 and various scan rates. General conditions: unless otherwise specified, CVs were recorded on a GC electrode (*A* = 7.07 mm^2^). All solutions were prepared in DMF + 0.1 M *n*-Bu_4_NBF_4_ and thermostated at 25 °C.

For aryl radicals, hydrogen abstraction from the solvent (HS), as illustrated at the bottom of Figure 6a, is a potential reaction pathway. However, regardless of whether R^•^, a solvent-derived radical S^•^, or a mixture of the two is formed in solution, their fate is governed by PDI^•–^ and PDI^2–^ through fast coupling or reduction processes.

The CV of PDI in the dark remained unchanged with or without 4-MCB, indicating no electrochemical reactivity for either PDI^•–^ or PDI^2–^ (Figure 6b). Bulk electrolysis of PDI at −1.2 V vs SCE in a spectroelectrochemical cell generated PDI^2–^ quantitatively and its spectrum is shown in Figure 6c. No significant evidence of preassociation between PDI^2–^ and 4-MCB was detected by CV and by UV-Vis-NIR of PDI^2–^ in the presence of 4-MCB (Figure S26b).

Irradiating PDI^2–^ with a 30 W 630 nm lamp generated *PDI^2–^ with *k*_exc_ = 86 s^–1^, calculated from eq 6, which is quite similar to the value found for PDI^•–^. The similarity between the *k*_exc_ values for PDI^•–^ and PDI^2–^ likely arises from their comparable absorbance at 630 nm. The lifetime of *PDI^2–^ was measured as 5.88 ns (see below), thus *k*_d_ = 1/τ_0_ = 1.70 × 10^8^ s^–1^.

Under irradiation and in the absence of 4-MCB, the CV of PDI showed increased cathodic peak current while the anodic peak current decreased, indicating enhanced mass transport (Figure 6d). Fitting the CV curves under irradiation with a finite diffusion model showed excellent correlation between experimental and simulated traces (Figure 6e), and δ = 73 ± 3 μm was obtained. A single value of δ was sufficient to fit the entire voltammetric curves across different scan rates. Both the δ value and the diffusion coefficient of PDI (*D* = (4.8 ± 0.4) × 10^–6^ cm^2^ s^–1^) closely matched those from the previous CV series (Figure 4b), reflecting similar absorption properties of PDI^•–^ and PDI^2–^ at 630 nm.

CV under irradiation in the presence of 4-MCB showed an intense catalytic current at the second cathodic peak, while the first peak remained unaffected, indicating negligible reactivity for *PDI^•–^ but high reactivity for *PDI^2–^ (Figure 6f). The current increase, which was proportional to the substrate concentration, was evident even at low values of substrate excess factor (γ = 0.25), highlighting the exceptional reactivity of *PDI^2–^.

CVs at varying γ and scan rates were fit to the mechanism in Figure 6a, with only *k*_SET_ and *k*_red_ as unknown variables (see Table S6 for a list of simulation parameters). The fitting showed excellent agreement between experimental and simulated traces (see examples in Figure 6g,h), yielding a value of log *k*_SET_ = 9.7, near the diffusion limit for a second order reaction (log *k*_diff_ = 10.2). This confirmed the high reactivity of *PDI^2–^. Additionally, a value log *k*_red_ = 8.9 was obtained, suggesting that PDI^2–^ is an efficient reductant for methyl benzoate radicals. This aligns with the negative reduction potential $E_{{PDI}^{•-}/{PDI}^{2-}}^{o}$ = −0.703 V vs SCE, making PDI^2–^ a strong reductant for phenyl radicals,^62^ especially in the presence of the ester electron-withdrawing group in 4-MCB.

With log *k*_c_ = 9.0 and log *k*_red_ = 8.9, our results indicate that the PDI^•–^ radical is relatively unstable during CV, undergoing competitive coupling with R^•^ or solvent-derived S^•^. This is reflected in Figure 6f,g, which show poorly reversible waves despite the low catalytic current, suggesting rapid PDI loss upon radical formation. Consistently, bulk electrolysis generating PDI^2–^/PDI^•^^–^ under 630 nm irradiation with 4-iodobenzaldehyde or 1-bromonaphthalene led to fast catalyst decomposition, confirmed by CV and UV-Vis-NIR (Figures S32 and S33). While structural evidence for PDI^•^^–^ coupling with R^•^ is lacking, the results align with reports of radical–radical combination involving aromatic radical anions.^63–66^ Notably, CV simulations excluding such coupling reactions failed to reproduce the data sets (Figures S23–S25).

The same CV method was applied to determine *k*_SET_ for the reaction between *PDI^2–^ and several aromatic and heteroaromatic halides and the results are reported in Table 3. In addition to 4-MCB, the substrates investigated were 4′-bromoacetophenone (4-BAP), 4-chlorobenzonitrile (4-CBN), 1-bromonaphthalene (1-BrNaph), iodobenzene (PhI), 2-bromopyridine (2-BrPy), 2-chloropyridine (2-ClPy), bromobenzene (PhBr) and chlorobenzene (PhCl). All structures are reported in Figure 2d.

## Assessment of the Obtained *k*_SET_ Values

### Quenching Rate Constants for *PDI^2–^

Experimental *k*_SET_ values from the voltammetric e-PRC method were validated through independent measurements. The more reactive and emissive *PDI^2–^ (Figure 7a) allowed direct determination of its quenching rate constant, *k*_q_, via time-controlled single photon counting (TCSPC) spectroscopy. Based on the mechanism in Figure 6a, the value of spectroscopic *k*_q_ should match that of voltammetric *k*_SET_.

**Figure 7:**
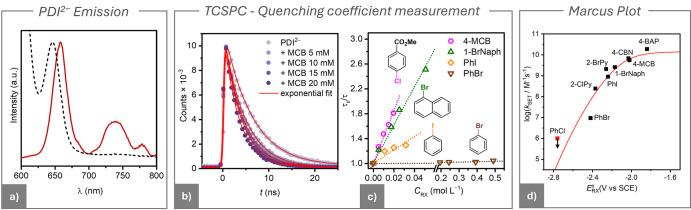
(a) Absorption (dotted line) and emission (solid line) spectra of 10^–4^ M PDI^2–^ in DMF + 0.1 M *n*-Bu_4_NBF_4_ recorded at room temperature in a 1 cm quartz cuvette. Excitation wavelength = 570 nm. PDI^2–^ was electrochemically generated by reducing a solution of PDI at −0.83 V vs SCE on a reticulated vitreous carbon electrode. (b) Stern–Volmer luminescence quenching experiments for PDI^2–^ with 4-MCB as quencher in 0.1 M *n*-Bu_4_NBF_4_ at room temperature. Luminescence decays were recorded at 657 nm using TCSPC after excitation at 633.6 nm, with various concentrations of 4-MCB. (c) Stern–Volmer plot derived from the luminescence lifetime data obtained. The dashed lines represent the linear regression fit. (d) Marcus plot correlating log *k*_SET_ values with the standard redox potential of various RX. The log *k*_SET_ data are averages derived from CV simulation data and TCSPC experiments (when available). The PhCl *k*_SET_ was excluded as only an upper limit for the value was provided.

PDI^2–^ was produced via bulk electrolysis of PDI at −0.83 V vs SCE in a degassed electrochemical cell and carefully transferred to a degassed cuvette. The lifetime of *PDI^2–^ in DMF + 0.1 M *n*-Bu_4_NBF_4_ was determined as τ_0_ = 5.88 ns (Figure 7b), which is consistent with the value reported in the literature for a similar perylene diimide in DMF.^20^ Addition of 4-MCB produced a sharp decrease in *PDI^2–^ lifetime, τ, in agreement with the occurrence of fast bimolecular SET (Figure 7b). Stern–Volmer plot of the obtained τ values, reported in Figure 7c, show a straight line (*R* ^2^ = 0.999) from which a Stern–Volmer constant *K*_SV_ = 42.5 M^–1^ and a quenching rate constant log *k*_q_ = 9.9 M^–1^ s^–1^ were obtained according to the following relationship

9 $$\tau_{0}/\tau =1+K_{SV}C_{RX}=1+k_{q}\tau_{0}C_{RX}$$

This log *k*_q_ value agrees with the value obtained via the voltammetric e-PRC method, log *k*_SET_ = 9.8 M^–1^ s^–1^, confirming the extreme reactivity of the *PDI^2–^ photocatalyst. The comparison with TCSPC quenching experiment was repeated for other aryl halides, 1-BrNaph (log *k*_q_ = 9.7 M^–1^ s^–1^, log *k*_SET_ = 9.4 M^–1^ s^–1^), PhI (log *k*_q_ = 9.2 M^–1^ s^–1^, log *k*_SET_ = 8.7 M^–1^ s^–1^) and PhBr (log *k*_q_ = 7.1 M^–1^ s^–1^, log *k*_SET_ = 6.9 M^–1^ s^–1^). Figure 7c shows the Stern–Volmer plots for *PDI^2–^ quenching by these substrates, while all results are summarized in Table 3.

### The Electron Transfer Mechanism of *PDI^2–^

The satisfactory agreement between *k*_q_ from quenching experiments and *k*_SET_ from voltammetry supports the mechanisms in Figures 3a and 6a, involving bimolecular electron transfer between the excited state catalyst and the substrate. The relationship between the rate constant for bimolecular electron transfer, *k*_SET_, and the driving force for the reaction, Δ*G*_ET_^o^, is often analyzed using various equations based on Marcus theory^67^ such as the Rehm–Weller equation^68^ and Marcus–Levine–Agmon equations.^69^ These equations provide relationships between the activation free energy, Δ*G* ^‡^, of the electron transfer reaction and its free energy change, Δ*G*_ET_^o^, which in the present system is given by

10 ΔGETo=F(EPDI•−/PDI*2−o−ERX/RX•−o)−NAe24πε0εσ

where *F* is Faraday’s constant, *N*_A_ is the Avogadro constant, *e* is the elementary charge, ε_0_ is the permittivity of the vacuum, and ε the relative permittivity of the solvent. The last term is the Columbic repulsion experienced by the products ion pair [PDI^•–^---RX^•–^] at a separation distance σ, which is small, 4.1 kJ mol^–1^ or 0.04 eV (Supporting Information, Section S5.2). The standard reduction potential of the excited-state catalyst was calculated as

11 EPDI•−/PDI*2−o=EPDI•−/PDI2−o−E0‐0

where *E*_0–0_ is the energy required to promote a molecule from its ground state to the zeroth vibrational level of the excited state. *E*_0–0_ was determined from the intersection of the normalized absorption and emission spectra^70,71^ (λ_int_ = 625 nm, Figure 7a), yielding *E*_0–0_ = *hc*/λ_int_ = 1.90 eV, where *h* is Planck’s constant and *c* is the speed of light. This led to an excited state potential EPDI•−/PDI*2−o = – 2.60 V vs SCE, which is in good agreement with the *E* ^o^ value reported for the excited dianion of a similar perylene diimide.^30^

In agreement with eq 10, since EPDI•−/PDI*2−o is constant, Δ*G*_ET_^o^ is directly proportional to $E_{RX/{RX}^{•-}}^{o}$ . Accordingly, log *k*_SET_ can be correlated with $E_{RX/{RX}^{•-}}^{o}$ in a Marcus plot as shown in Figure 7d for the series of investigated aryl halides. For acceptors with $E_{RX/{RX}^{•-}}^{o}$ > −2.1 V vs SCE, quenching approached the diffusion limit for a bimolecular reaction (∼2 × 10^10^ M^–1^ s^–1^). Instead, the expected decrease of *k*_SET_ as a function of decreasing $E_{RX/{RX}^{•-}}^{o}$ was found for acceptors with redox potentials more negative than −2.1 V vs SCE.

The experimental log *k*_SET_ versus $E_{RX/{RX}^{•-}}^{o}$ were fitted to the Marcus–Levine–Agmon equation (Supporting Information, eq S5.16), leaving the reorganization energy for electron transfer (Δ*G*_0_^‡^ = λ/4) as the only adjustable variable. The solid line in Figure 7d represents the best fit yielding λ = 117 ± 25 kJ mol^–1^. This result is in good agreement with the λ value reported by Daasjberg et al.^57^ for a large series of aromatic halides, λ = 108–156 kJ mol^–1^, as well as previous work of Wayner and co-workers.^72^

This high λ value, significantly larger than typical solvent reorganization values (λ_i_ = 60–70 kJ mol^–1^), indicates substantial internal reorganization upon electron transfer between *PDI^2–^ and aryl halides.^54^ This aligns with the significant internal reorganization energy of organic halides (λ_i_ = 22–68 kJ mol^–1^),^42^ due to C–X bond stretching upon electron transfer. Additionally, we have estimated a relatively high reorganization energy for the catalyst *PDI^2–^, which was calculated via computational methods as λ_i_ = 25 kJ mol^–1^ for the PDI^•–^/*PDI^2–^ couple (see Supporting Information, Section S6). This large internal reorganization energy value is consistent with the observed low heterogeneous electron transfer rate constants (*k* ^0^). To further confirm the large internal reorganization energy for this catalyst system we estimated a value of λ_i_ = 28 kJ mol^–1^ for the PDI/PDI^•–^ couple on the basis of the experimentally measured *k*° (see Supporting Information, Section S5.3). We were not able to carry out a similar estimate for the PDI^•–^/PDI^2–^ couple. We may, however, assume similar λ_i_ values for the two redox couples by virtue of their almost identical *k*° values.

In summary, good agreement between the Marcus–Levine–Agmon equation and the experimental *k*_SET_ values suggested that the reaction between *PDI^2–^ and RX proceeds via outer sphere electron transfer (OSET) with the contribution of internal reorganization of both reacting partners upon ET.

### Quenching Rate Constants for *PDI^•–^

Returning to the radical anion *PDI^•–^, comparing voltammetric *k*_SET_ values with quenching *k*_q_ values was more challenging because this open shell catalyst is not emissive. Using picosecond time-resolved transient absorption spectroscopy (TAS), Schanze et al. reported *k*_q_ values between 10^10^ and 10^8^ M^–1^ s^–1^ for the reaction of *PDI^•–^ with halobenzaldehydes and nitrobenzenes.^29^ However, these substrates exhibit slow or no bond cleavage after SET, unlike the aryl and alkyl halides in Table 3, which undergo fast or concerted bond cleavage upon SET (*k*_fr_ > 10^7^ M^–1^ s^–1^).^57,73,74^ For instance, *k*_fr_ = 630 M^–1^ s^–1^ for 4-chlorobezaldehyde. This strongly affects the CV method.

Applying the voltammetric e-PRC approach to *PDI^•–^ and 4-nitroanisole, investigated by Schanze et al. (log *k*_q_ = 9.8), gave no catalytic current (Figure 8a). Similarly, 4-bromobenzaldehyde (log *k*_q_ = 9.8) showed no current enhancement (Figure S13). In contrast, 4-Iodobenzaldehyde (log *k*_q_ = 9.5) yielded a modest amount of catalytic current, from which log *k*_SET_ > 7.3 could be estimated (Figure S12).

**Figure 8:**
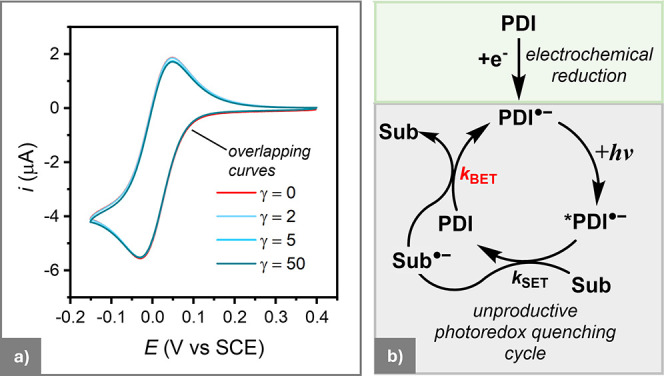
(a) CVs of 10^–3^ M PDI under 630 nm light irradiation in the presence and in the absence of 4-nitroanisole (Sub) at different concentrations labeled on the curves (γ = *C*_Sub_/*C*_PDI_), recorded in DMF + 0.1 M *n*-Bu_4_NBF_4_ on a GC electrode (*A* = 7.07 mm^2^) at *T* = 25 °C and *v* = 0.02 V s^–1^. (b). Unproductive photoredox cycle dominated by back electron transfer, in case of Sub^•–^ characterized by no or slow bond cleavage.

The lack of, or only minimal, catalytic current was attributed to the unproductive photoredox quenching cycle in Figure 8b. After electrochemical reduction and photon absorption, *PDI^•–^ is formed, which can swiftly react with the substrate (Sub), forming Sub^•–^. However, since no or slow bond breaking occurs, fast back electron transfer (BET) between Sub^•–^ and PDI takes place at a diffusion-controlled rate (*k*_BET_ ∼ 10^10^ M^–1^ s^–1^) because $E_{Sub/{Sub}^{•-}}^{o}$ ≪ $E_{PDI/{PDI}^{•-}}^{o}$ . This regenerates PDI^•–^ and effectively short-circuits the catalytic cycle, preventing any meaningful catalytic current increase.

The absence of electrophotocatalytic current despite fast substrate quenching highlights a key difference between time-resolved methods such as TAS and electrocatalytic methods such as voltammetry under e-PRC conditions. TAS focuses on the quenching step, providing lifetime and *k*_q_ values regardless of following reactions such as BET. In contrast, the voltammetric e-PRC method monitors the full catalytic cycle, showing no (or tiny) current if the overall reaction is unproductive. Thus, the two techniques offer complementary insights on photoredox reactivity, which could be useful in future studies on back electron transfer, cage escape, or electron ejection.

Due to the lack of literature data for direct comparison between voltammetric and spectrometric *k*_SET_ values of alkyl halides, the reaction between *PDI^•–^ and RX was modeled with Marcus OSET theory, with theoretical *k*_SET,th_ calculated as

12 $$k_{SET,th}=Z\exp\left(-\frac{\Delta G^{‡}}{RT}\right)$$

where *Z* is the pre-exponential factor. Δ*G* ^‡^ was calculated using the Marcus–Hush theory and subsequent modifications by Savéant (details in Supporting Information, eqs S5.3–S5.11).^44,45,75,76^ All calculated *k*_SET,th_ values from eq 12 aligned, within the simplified model accuracy, with experimental *k*_SET_ data from CV (Table 4). These results are consistent with Marcus analysis by Schanze et al.,^29^ which indicated a bimolecular OSET quenching pathway for the same catalyst, further validating voltammetric e-PRC as a reliable method for determining *k*_SET_.

**Table 4:** Comparison between *k*_SET,th_ and *k*_SET_ Data for the Reduction of Alkyl Halides by *PDI^•–^

entry	RX	*E*_RX_^o^ (V vs SCE)	Δ*G* ^‡^ (kJ mol^–1^)	log *k*_SET,th_ ^a^	log *k*_SET_
1	MBiB	–0.52	21.7	8.0	8.0
2	EBiB	–0.46	20.0	8.3	7.8
3	BrACN	–0.49	24.0	7.7	8.1

a Calculated from eq 12.

## Conclusions

This study used cyclic voltammetry under electrode irradiation to quantify the reactivity of short-lived excited-state catalysts employed in e-PRC and conPET systems. Light-induced catalytic currents were observed when reducing PDI in the presence of alkyl and aryl halides, enabling rapid screening of photoredox activity. For instance, the difference in photoreactivity between electrogenerated PDI^•*–*^ and PDI^2–^ was immediately apparent, with *PDI^2–^ being significantly more reactive and producing much larger catalytic currents. We anticipate this rapid photoelectrochemical screening method to be broadly useful as a kinetic probe for various e-PRC and conPET systems.

Additionally, by combining experimental CV data with digital simulations, we outlined a robust quantitative framework to determine the single-electron transfer rate coefficient (*k*_SET_), a key parameter for excited-state reactivity. Direct measurements of quenching kinetics were obtained by analyzing the recorded catalytic current and the reversibility of the PC/PC^•–^ wave. By adjusting the substrate concentration and the sweep rate, this voltammetric method enabled monitoring both ultrafast (*k*_SET_ > 10^10^ M^–1^ s^–1^) and relatively slow (*k*_SET_ ∼ 10^7^ M^–1^ s^–1^) quenching reactions.

Using this approach, we quantified the reactivity of *PDI^•–^ with several activated alkyl halides, finding moderate *k*_SET_ = 10^7^–10^8^ M^–1^ s^–1^. Conversely, the two-electron reduced *PDI^2–^ exhibited exceptional reducing power, capable of cleaving at diffusion-controlled rates (*k*_SET_ ∼ 10^10^ M^–1^ s^–1^) C–X bonds in recalcitrant aryl halides with reduction potentials of ca. −2.0 V vs SCE. *PDI^2–^ also demonstrated detectable reactivity with substrates having reduction potentials as low as −2.4 V vs SCE, such as bromobenzene (*k*_SET_ ∼ 10^7^ M^–1^ s^–1^).

Mechanistic analysis suggested that these excited-state catalysts reduced RX via outer-sphere bimolecular electron transfer (OSET), enabled by the relatively long lifetimes of the excited-state catalysts. In the case of *PDI^2–^, minor energy penalty arose from its relatively high internal reorganization energy (λ_i_ ∼ 0.3 eV) and from the Coulombic repulsion (0.04 eV) between the products (PDI^•–^ and RX^•–^). However, these minor factors were compensated for by the highly negative reduction potential of *PDI^2–^ ( EPDI•−/PDI*2‐o = – 2.6 V vs SCE) and its relatively long lifetime (τ_0_ ∼ 6 ns), enabling exceptional photoreactivity.

Compared to time-resolved techniques like TCSPC or TAS, the voltammetric e-PRC method could provide rich complementary information on photoredox cycles. Indeed, it was sensitive not only to the rate of the quenching step (PC* + substrate), but also to the efficiency of the overall electrophotocatalytic cycle, showing no current enhancement if there was no net chemical reactivity due to back electron trasnfers. Thus, the CV method effectively complemented time-resolved spectroscopic techniques.

Looking forward, we anticipate that CV under irradiation will provide important mechanistic insights and accelerate the discovery of new photocatalytic systems for challenging reduction and oxidation reactions. This electrochemical methodology could open new pathways for investigating aspects of photochemistry including the efficiency of cage-escape, electron donor–acceptor (EDA) complexes, the generation of solvated electrons, and preassociation effects in subnanoseconds photocatalysis.

## Methods

### Instrumentation

UV-Vis-NIR spectra were acquired on an Agilent Cary 60 spectrophotometer. Steady-state and time-resolved fluorescence measurements were performed on an Edinburgh Instruments FLS 1000 spectrometer using a 450 W xenon lamp (steady-state) or a 633.6 nm pulsed diode laser with TCSPC (time-resolved, 10^5^–10^7^ cps). Decays were fitted using Fluoracle software (IRF convolution, single-exponential). Electrochemical experiments used Autolab PGSTAT30 or PGSTAT204 potentiostats. Lamp irradiance was measured with an Avantes AvaSpec spectrophotometer.

### Electrochemical Measurements

CV was conducted in a 5-neck jacketed cell using a GC disk working electrode (WE), Pt wire counter electrode (CE), and an Ag|AgI|I^–^ reference electrode (RE, 0.1 M *n*-Bu_4_NI in DMF), calibrated postexperiment against Fc^+^|Fc and referenced to SCE. The GC WE was routinely polished (0.25 μm diamond paste) and sonicated/rinsed (ethanol/acetone). Before measurements, 0.1 M electrolyte solution (10 mL) was purged with Ar (30 min), background CVs recorded, reactants added under Ar, purged again (15–20 min), and experimental CVs recorded with resistance compensation.

### Spectroelectrochemical (SEC) Measurements

SEC used a BAS Inc. kit with a quartz cell (0.5 mm path length), Pt gauze WE (80 mesh, activated by cycling in 0.5 M H_2_SO_4_), Pt CE, and Ag|Ag^+^ RE. An Autolab PGSTAT204 potentiostat and Cary 60 spectrophotometer were used, acquiring spectra under Ar flow after baseline correction (solvent + electrolyte).

### Photochemical Setup and Photocyclovoltammetry

Irradiation used a custom 630 nm, 30 W LED powered by a Peak Tech P 6225 A current generator (1400 mA, 9.4 V), placed below the electrochemical cell (see setup details and pictures in the Supporting Information).

### Computational Methods

Density functional theory (DFT) and time dependent (TD)-DFT calculations were performed with Gaussian16 Rev C.01. Geometries were optimized at the B3LYP/6–31G­(d,p) level, confirmed as minima by frequency calculations. Excited states were calculated at the TD-B3LYP/6–31G­(d,p) level, including DMF solvation via the SMD model (SMD-TD-B3LYP/6–31G­(d,p)). NTOs were visualized, reorganization energies calculated via Nelsen’s four-point method, and spin contamination checked.

## Supplementary Material



## Notes

Detailed material descriptions and procedures, electrochemical investigation of PDI, solution temperature in the diffusion layer, computational results, full mechanism for CV simulation, experimental CVs and simulation data for RX substrates (PDF)
