# Supplementary material for: Rapid Electrochemical Assessment of Excited-State Quenching Dynamics
Source: ACS Catal. 2025 Sep 23;15(19):16938–52. doi: 10.1021/acscatal.5c02778 (PMC12502628; doi:10.1021/acscatal.5c02778)
Supplement: Supplementary file 1 [file cs5c02778_si_001.pdf]

Supplementary material for

Rapid Electrochemical Assessment of Excited-State Quenching Dynamics

Tobia Casadei, Alberto Piccoli, Davide Zeppilli, Laura Orian, Abdirisak A. Isse, Marco Fantin*

Department of Chemical Sciences, University of Padova, Via Marzolo 1, Padova 35131, Italy

Corresponding author email: marco.fantin@unipd.it

Contents

1 Materials and methods ... 2

2 Procedures ... 4

3 Detailed electrochemical investigation of PDI ... 6

3.1 Standard heterogeneous electron transfer rate constant (k^0) ... 6

3.2 Diffusion coefficients ... 7

3.3 Evaluation of correction factors for the values of k_{exc} ... 8

4 Solution temperature in the diffusion layer and reactivity in dark at high temperatures ... 10

5 Assessment of the experimentally obtained k_{SET} values ... 11

5.1 Kinetic analysis of concerted dissociative electron transfer: $*PDI^{•-}$ reactivity with alkyl halides 11

5.2 Marcus plot for stepwise dissociative electron transfer: $*PDI^{2-}$ reactivity with aryl halides ... 13

5.3 Estimation of the reorganization energy of the $PDI/PDI^{•-}$ couple ... 15

6 Computational results for the reorganization energy of $*PDI^{2-}$ to $PDI^{•-}$ ... 18

7 Full mechanism for CV simulations ... 20

7.1 Reactivity of $*PDI^{•-}$ ... 20

7.2 Additional considerations on the electrophotocatalysis with PDI ... 21

7.3 Reactivity of the excited state of the dianion catalyst, $*PDI^{2-}$ ... 21

7.4 Evaluating the impact of the rate of C−X bond cleavage ... 23

7.5 Sensitivity analysis of k_{SET} and k_{red} values obtained by digital simulation ... 24

8 Experimental and simulated CVs for RX substrates ... 25

8.1 Ehtyl α-bromoisobutyrate (EBiB) ... 25

8.2 Bromoacetonitrile (BrACN) ... 26

8.3 4’-Iodobenzaldehyde (4-IBA) ... 27

8.4 4′-Bromoacetophenone (4-BAP) ... 29

8.5 4-Chlorobenzonitrile (4-CBN) ... 30

8.6 1-Bromonaphthalene(1-BrNaph) ... 31

8.7 Iodobenzene (PhI) ... 32

8.8 2-Bromopyridine (2-BrPy) 33
8.9 2-Chloropyridine (2-ClPy) 34
8.10 Bromobenzene (PhBr) 35
8.11 Chlorobenzene (PhCl) 36
9 Experimental TCSPC data with relative fitting 37
10 Fitting evaluation excluding the $PDI^{•–}$ + $R^•$ coupling reaction 38
11 Spectroelectrochemistry in the presence of RX 40
12 CVs under different wavelengths 41
13 Constant potential electrolysis 43
13.1 CPE of MBiB 43
13.2 CPE of 4-IBA 45
13.3 CPE of 1-BrNaph 46
14 Estimation of energy loss for photocatalysis with $*PDI^{•–}$ 47

1 Materials and methods

Materials

The solvent was HPLC grade *N,N*-dimethylformamide (DMF, 99.9% Carlo Erba). The supporting electrolyte, tetrabutylammonium tetrafluoroborate (n-Bu_4NBF_4, Alfa Aesar, 98%), was recrystallized twice from hot ethyl acetate and dried in a vacuum oven at 80 °C, over two days, and then stored over P_2O_5. *N,N'*-Bis(2,6-diisopropylphenyl)-3,4,9,10-perylenetetracarboxylic diimide (PDI, TCI America, > 98.0%), methyl 2-bromopropionate (MBP, Sigma-Aldrich, 98%), ethyl 2-bromopropionate (EBP, Sigma-Aldrich, 98%), methyl α-bromoisobutyrate (MBiB, Sigma-Aldrich, ≥ 99.0%), ethyl α-bromoisobutyrate (EBiB, Sigma-Aldrich, 98%), bromoacetonitrile (BrACN, Sigma-Aldrich, 97%), methyl 4-chlorobenzoate (4-MCB, Sigma-Aldrich, 99%), 4’-bromoacetophenone (4-BAP, Sigma-Aldrich, 98.0%), 4-chlorobenzonitrile (4-CBN, Sigma-Aldrich, 99.0%), 1-bromonaphthalene (1-BrNaph, Sigma-Aldrich, 98%), iodobenzene (PhI, Sigma-Aldirch, 98%), 2-bromopyridine (2-BrPy, Sigma-Aldrich, 98%), 2-chloropyridine (2-ClPy, Sigma-Aldrich, 98%), bromobenzene (PhBr, Sigma-Aldrich, 99%), chlorobenzene (PhCl, Sigma-Aldrich, 98%), and ferrocene (Fc, 98% Acros Organics) were used as received. All the other reagents were of high commercial grade and used without further purifications.

Instruments

UV-Vis-NIR. Spectroscopic measurements were carried out on an Agilent Cary 60 UV-vis spectrophotometer (Xenon flash lamp, 80 Hz), connected to a computer with Cary WinUV software.

Fluorescence. For fluorescence measurements, the FLS 1000 UV/Vis/NIR photoluminescence spectrometer (Edinburgh Instruments Ltd.) was used. The excitation source was a 450 W Xenon arc lamp that emits continuous radiation from 230 nm to over 1000 nm. The detector used was the air-cooled single-photon counting photomultiplier (Hamamatsu R13456). This is a side-window photomultiplier with extended sensitivity in the near-infrared range, covering a spectral range from 185 nm to 980 nm.

For time-resolved lifetime measurements, the Time-Correlated Single Photon Counting (TCSPC) technique was used. The sample was excited by a high-frequency pulsed light source (laser diode at 633.6 nm) with a typical repetition rate of 10^5-10^7 cps. Decay curves were analyzed with the Fluoracle Software using the IRF convolution and one-exponential component model fitting.

Electrochemistry. Electrochemical experiments were carried out either with an Autolab potentiostat (PGSTAT30, Utrecht, The Netherlands) run by a computer with GPES software or an Autolab PGSTAT204 potentiostat/galvanostat (Eco-Chemie, Utrecht, Netherlands), run by Nova 2.1 software. All experiments were performed in a jacketed 5-neck glass electrochemical cell. The electrodes used in cyclic voltammetry were a glassy carbon (GC) disk as the working electrode, a platinum (Pt) wire as the counter electrode, and an Ag|AgI|I^- reference electrode prepared by dipping an AgI-coated Ag wire in 0.1 M *n*-Bu_4NI in DMF. The reference electrode was calibrated against the ferrocenium/ferrocene couple after each experiment. Before its first use, the electrochemical cell was cleaned with *aqua regia* (**a highly corrosive and oxidizing cleaning solution!**), and abundantly washed with deionized water followed by HPLC grade acetone, and dried in an oven at ~60 °C.

Spectroelectrochemistry. Spectroelectrochemistry experiments were carried out with a kit by BAS Inc, comprised of a thin layer quartz glass cell (0.5 mm light pathlength), a Pt gauze working electrode (80 mesh), a Pt counter electrode, and a non-aqueous Ag wire pseudo-reference electrode. The Pt gauze was activated by cycling in 0.5 M H_2SO_4 until a reproducible signal was observed.

NMR. NMR was carried out with a Bruker 400 MHz instrument in $CDCl_3$.

Lamp irradiance. Absolute irradiance of the lamp was measured with an Avantes AvaSpec spectrophotometer. The probe was placed in the exact position as the surface of the working electrode. The measured $I(\lambda)$ was used to determine the value of k_{exc} via eq. 6 in the main text.

Lamp. The red lamp was constructed from a 630 nm 30 W LED (Aftertech.eu, model eb-164891694865). The LED was powered with a current generator Peak Tech® P 6225 A (run at 1400 mA and a forward voltage of 9.4 V) and cooled with a computer fan.

Computational methods. All Density Functional Theory (DFT) and Time Dependent DFT (TD-DFT) calculations were carried out using Gaussian16 Rev C.01.[1] B3LYP[2-3] hybrid functional was used with 6-31G(d,p) basis set for all geometry optimizations (level of theory: B3LYP/6-31G(d,p)). The same combination of potential and basis set was used to calculate the lowest ten excitation energies and optimize the lowest excited state (level of theory: TD-B3LYP/6-31G(d,p)) Frequency calculations were performed for all fully optimized structures to assess that all minima have real frequencies. Solvation effects in DMF were included in the excited states calculations using SMD[4] (level of theory: SMD-TD-B3LYP/6-31G(d,p)). Orbitals involved in the lowest electronic transition were visualized as Natural Transition Orbitals (NTOs).[5] The reorganization energy for the electron transfer was calculated according to the Nelsen's four-point method.[6-8] Spin contamination was checked for doublet species and was found to be negligible.

2 Procedures

Cyclic voltammetry (general procedure). For voltammetric studies, a 3 mm glassy carbon (GC) disk electrode was used. To ensure reproducibility, thorough cleaning was performed after extended inactivity or passivation, while routine cleaning was sufficient for daily use.

Thorough cleaning:

1. Polished to a mirror finish with silicon carbide papers (800, 1000, 2400, 4000 grit) and diamond pastes (3, 1, and 0.25 μm) on Buelher® cloths.
2. Sonicated in absolute ethanol for 5 minutes after each polishing.
3. Rinsed with HPLC-grade acetone.

Routine cleaning:

1. Polished with 0.25 μm diamond paste.
2. Sonicated in ethanol.
3. Rinsed with HPLC-grade acetone.

The electrochemical cell was loaded with 10 mL of 0.1 M supporting electrolyte, which was then stirred and purged with argon for 30 minutes. Background CVs were recorded before reactants were added under an argon flux. This was followed by an additional 15-20 minutes of purging before recording the experimental CVs. Resistance compensation was applied during the measurements. After the experiments, the cell was cleaned sequentially with ethanol and acetone and stored at 60 °C.

Cyclic voltammetry under light irradiation. The light source was placed beneath the electrochemical cell, which had glass-jacketed sides and a flat glass bottom without a jacket (Figure S1). Blank CVs were first

recorded at operational scan rates. PDI was then added at a concentration of 10^{-3} M or $5 \cdot 10^{-4}$ M, and CVs were measured in the dark and under irradiation. Finally, a substrate was introduced, and CVs under irradiation were recorded, with substrate concentrations adjusted as needed.

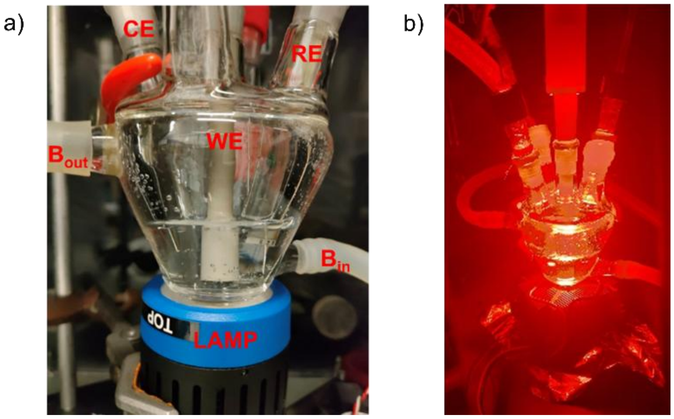

Figure S1. Cell setup (a) with no irradiation and (b) with active irradiation (with a 630 nm 30 W LED array lamp). B_{in} and B_{out} stand for thermostat water inlet and outlet, respectively.

The measured potentials were referred to the SCE by recording a voltammogram of the $Fc^+|Fc$ couple at the end of each CV experiment. This allowed calibration of the non-aqueous Ag/AgI electrode and conversion of potentials to the SCE scale by using:[9-10]

$$E^\circ_{Fc^+/Fc} = 0.476 \text{ V vs SCE in DMF} + 0.1 \text{ M } Et_4NBF_4 \qquad (2.1)$$

Spectroelectrochemistry. The electrodes and the degassing system were arranged as in Figure S2. Degassing was achieved using a 2 L argon-filled gas balloon. The Pt gauze working electrode (WE) was activated by cycling in 0.5 M H_2SO_4 (aqueous) from −0.25 V to 1.2 V vs SCE until reproducible CVs were obtained.

For spectroelectrochemistry, an Autolab PGSTAT204 potentiostat was employed. Baseline measurements (DMF + 0.1 M supporting electrolyte) were recorded under a constant argon flow. UV-Vis spectra were collected using a Cary 60 spectrophotometer.

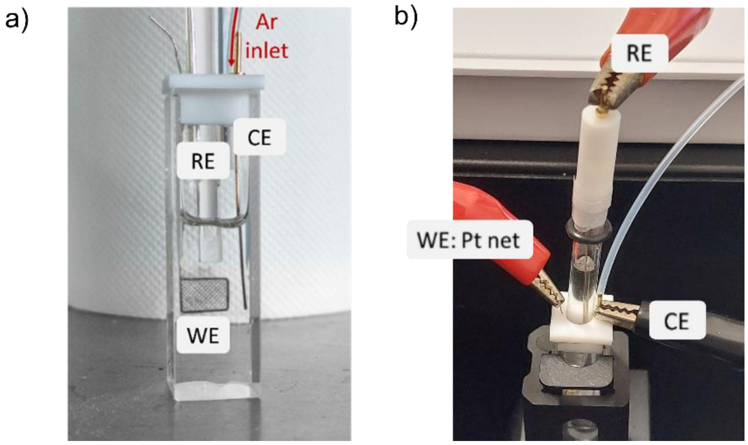

Figure S2. Spectroelectrochemical setup a) side view and b) from above, when inserted in the spectrophotometer.

3 Detailed electrochemical investigation of PDI

3.1 Standard heterogeneous electron transfer rate constant (k^0)

The standard heterogeneous electron transfer rate constant (k^0) for PDI/PDI$^{\bullet-}$ and PDI$^{\bullet-}$/PDI^{2-} was measured using the Nicholson method,[11] which relates the peak separation (ΔE_p) to the kinetic parameter Ψ.

$$\Psi = \frac{\left(\frac{D_O}{D_R}\right)^{\alpha/2} k^0}{\left[\left(\frac{nF}{RT}\right)\pi D_O \upsilon\right]^{1/2}} \tag{3.1}$$

where n is the number of exchanged electrons, F the Faraday's constant, R the gas constant, and T temperature. Assuming equal diffusion coefficients for the oxidized and reduced species ($D_O = D_R$), Ψ simplifies to:

$$\Psi = \frac{k^0}{\left[\left(\frac{nF}{RT}\right)\pi D_O \upsilon\right]^{1/2}} \tag{3.2}$$

A theoretical curve of ΔE_p vs. logΨ was obtained by fitting Nicholson's theoretical ΔE_p data with a fifth-degree polynomial:

$$y = 0.08391 - 0.05274x + 0.05387x^2 - 0.02649x^3 - 0.00086x^4 + 0.00411x^5 \tag{3.3}$$

where, $y = \Delta E_p$ and $x = \log\Psi$. Experimental ΔE_p values were collected at different scan rates. Then logΨ' was calculated for each scan rate:

$$\log \Psi' = \log \left\{ \frac{1}{\left[\left(\frac{nF}{RT} \right) \pi D_O \upsilon \right]^{1/2}} \right\} \quad (3.4)$$

Using a nonlinear regression based on equation (3.3) of the experimental data for ΔE_p against log Ψ', the value of log k^0 was obtained. In the regression, an independent variable is set as $x = \log \Psi' + P$, and the fitting process optimizes the constant $P = \log k^0$. Experimental data fitting on the working curve are shown in Figure S3. The obtained values for the heterogenous electron transfer rate constant were $k^0 = 0.019$ cm s^{-1} for PDI/PDI$^{\bullet-}$ and $k^0 = 0.020$ cm s^{-1} for PDI$^{\bullet-}$/PDI^{2-}.

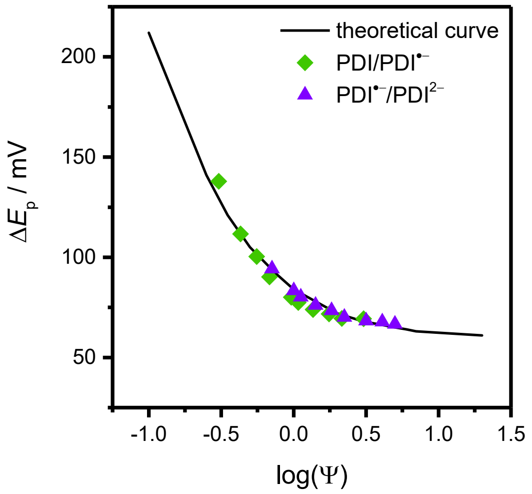

Figure S3. Fitting of experimental ΔE_p vs logΨ' data relative to the first reversible reduction of PDI (green) and the second reversible reduction of PDI (purple) on the theoretical curve (black line).

3.2 Diffusion coefficients

The diffusion coefficient (D) of PDI was determined from the cathodic peak current (i_{pc}) at different scan rates via the Randles–Ševčik equation (3.5):

$$i_{pc} = 0.4463 nFAC \sqrt{\frac{nF\upsilon D}{RT}} \quad (3.5)$$

where n is the number of exchanged electrons (in our case one), F is the Faraday constant (96485 C mol^{-1}), A is the working electrode surface, C is the concentration of the species, υ is the scan rate, R is the gas constant (8.314 J K^{-1} mol^{-1}) and T is the working temperature. Rearranging equation (3.5) into equation (3.6) allows calculation of the diffusion coefficient D from the slope of i_{pc} vs $\upsilon^{1/2}$ plot (Figure S4b).

$$D = \frac{RT\left(\partial i_{p,c}/\partial \upsilon^{1/2}\right)^2}{nF(0.4463nFC)^2} \tag{3.6}$$

The experimentally obtained value is $D_{PDI} = 3.3 \cdot 10^{-6}$ cm^2 s^{-1}.

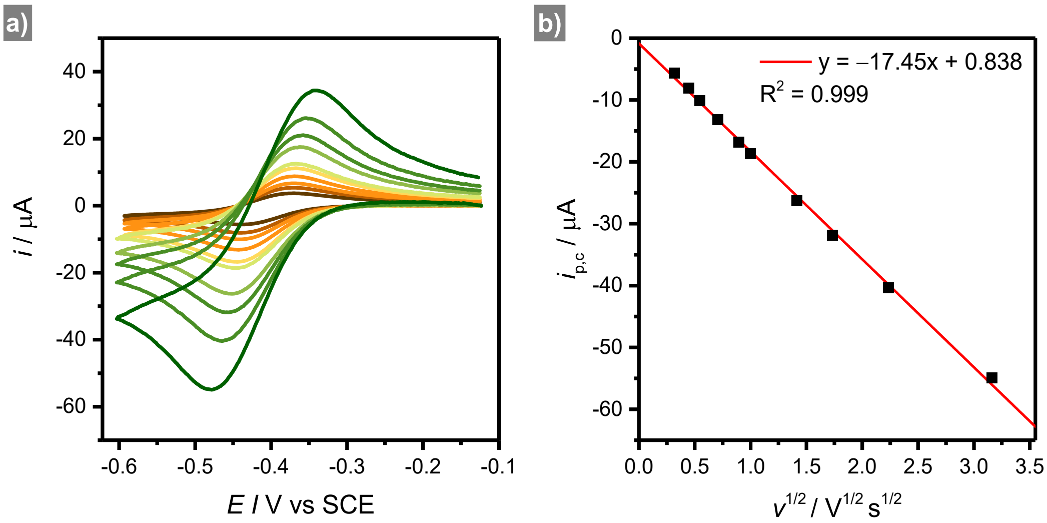

Figure S4. a) Background subtracted CVs of $5 \cdot 10^{-4}$ M PDI in DMF + 0.1 M n-Bu_4NBF_4 on a GC electrode (A = 7.07 mm^2) at T = 25 °C. Scan rates = 0.1 V s^{-1} – 10 V s^{-1}. b) Linear fit of background-subtracted i_{pc} vs $\upsilon^{1/2}$.

3.3 Evaluation of correction factors for the values of k_{exc}

Two correction factors, $(1 + R)$ and T, must be considered to calculate the effective coefficient k'_{exc} from the value of k_{exc} calculated by Eq. (6) of the main text:

$$k'_{exc} = k_{exc}(1{+}R)T \tag{3.7}$$

where R is the fraction of light reflected by the electrode surface, and T is the transmittance of the diffusion layer due to light absorption by $PDI^{\bullet-}$.

Light reflected by the electrode (R) enhances excitation efficiency. For a polished GC surface, R = 0.16 was measured by determining the fraction of light reflected when a 5 mW 630 nm laser was directed onto freshly

polished GC using an Avaspec spectrometer. This suggests that the electrode reflected 16% of incident red light, indicating that a $(1 + R) = 1.16$ correction factor should be applied to account for light reflection.

Transmittance of the diffusion layer was then considered. The reduced species of PDI absorbs light in the diffusion layer near the electrode surface, potentially lowering transmittance and reducing the effective light reaching the electrode. The transmittance (T) varies during the CV scan as the diffusion layer thickness changes. For instance, in the case of electrogenerated $PDI^{\bullet-}$ illuminated with 630 nm light, the maximum determined diffusion layer thickness (δ) was 73.3 ± 2.5 μm. Using the Lambert-Beer law, the maximum fraction of light absorbed by $PDI^{\bullet-}$ can be calculated, and the transmittance is given by:

$$T = 10^{-\varepsilon\delta C/2} \tag{3.8}$$

where ε is the extinction coefficient of $PDI^{\bullet-}$ (15,000 M^{-1} cm^{-1} at 630 nm). The factor 1/2 in the exponent accounted for the fact that average $PDI^{\bullet-}$ concentration in the diffusion layer is approximately half of PDI bulk concentration ($C_{bulk} = 10^{-3}$ M). See Figure 4c in the main text for a graphical representation of the maximum diffusion layer thickness. From Eq. 3.8, the transmittance (T) was calculated as 0.88 (88%), indicating that 12% of the light was absorbed. Given that the correction factors for transmittance ($T = 0.88$) and light reflection ($1 + R = 1.16$) were relatively close to unity and had opposing effects, tending to cancel each other out, they were neglected in our calculations. This simplification introduced a minor error but significantly streamlined the numerical analysis.

Further comments and guidelines:

To apply the CV method described in this work, it is necessary to maintain the value of T as close to 1 as possible, ensuring constant light intensity on the reduced catalyst in the diffusion layer. In accordance with the Lambert-Beer law, this can be achieved by lowering the catalyst concentration or by using irradiation wavelengths where the catalyst's absorption coefficient is not extremely high.

The value of R should also be considered. It could be increased, for example, by using metal electrodes instead of glassy carbon. For polished platinum, $R \approx 1$, effectively doubling the amount of light reaching the catalyst, which could allow the CV investigation to be carried out with lower intensity lights.

4 Solution temperature in the diffusion layer and reactivity in dark at high temperatures

To calculate the effective temperature at the electrode diffusion layer under irradiation, a calibration curve was constructed by measuring the diffusion coefficient of PDI as a function of temperature (in the dark). The diffusion coefficients were determined via CV using the Randles–Ševčik equation and fitted via the Arrhenius equation (Figure S5).

The diffusion coefficients of PDI under illumination was determined as $D = (4.8 \pm 0.4) \cdot 10^{-6}$ cm^2 s^{-1}. Utilizing the calibration curve in Figure S5a, this corresponded to a diffusion layer temperature of 49 ± 4 °C.

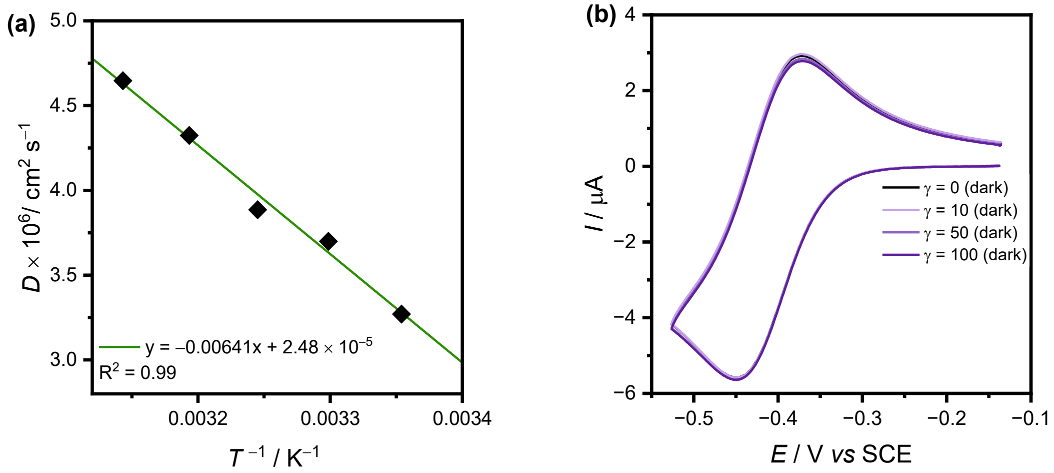

Figure S5. a) Calibration plot relating the diffusion coefficient (D) of PDI to temperature (T), used to determine the effective temperature in the diffusion layer. Diffusion coefficients were obtained at known temperatures (range: 25–45 °C) from CV scans using the Randles–Ševčik equation. Experimental conditions: 10^{-3} M PDI in DMF + 0.1 M n-Bu_4NBF_4 on a GC electrode (A = 7.07 mm^2) at T ranging from 25 °C to 45 °C; scan rate $\upsilon = 0.02 - 10$ V s^{-1}. b) CV of 10^{-3} M PDI in the presence of different amounts of MBiB (labelled on the curves as $\gamma = C_{RX}/C_{PDI}$) at 0.02 V s-1 at 50 °C in DMF + 0.1 M n-Bu_4NBF_4 on GC electrode.

No reactivity in the dark between $PDI^{\bullet-}$ and MBiB was observed when thermostating the cell at 50 °C, indicating that the reactivity between the two what not induced thermally, but photochemically. This was confirmed by the overlapping CVs of PDI in the absence and in the presence of a large excess of MBiB (Figure S5b).

5 Assessment of the experimentally obtained k_{SET} values

The k_{SET} values obtained via the voltammetric e-PRC method for reactions involving $^*PDI^{•-}$ and $^*PDI^{2-}$ were studied with a theoretical approach involving Marcus theory and subsequent modifications.

5.1 Kinetic analysis of concerted dissociative electron transfer: $^*PDI^{•-}$ reactivity with alkyl halides

The electrophotochemical reduction of a series alkyl halides (RX) by $^*PDI^{•-}$ was studied:

$$^*\mathrm{PDI}^{\bullet-} + \mathrm{RX} \rightarrow \mathrm{PDI} + \mathrm{R}^{\bullet} + \mathrm{X}^{-} \quad (5.1)$$

This reaction involves an outer sphere electron transfer (OSET) between $^*PDI^{•-}$ (electron donor) and RX (electron acceptor). Alkyl halides undergo a concerted dissociative electron transfer (CDET), where bond cleavage occurs alongside electron transfer.[12-16] The kinetics can be studied using the Marcus–Savéant model and subsequent modifications.[13-15]

The rate constant of reaction (5.1) can be calculated as:

$$k_{\mathrm{SET,th}} = Z \exp\left(-\frac{\Delta G^{\ddagger}}{RT}\right) \quad (5.2)$$

where Z is the collision frequency and $\Delta G^{\ddagger}$ is the activation free energy of the reaction.

For the CDET, the "sticky model" was considered, and the value of $\Delta G^{\ddagger}$ was calculated via the following equations:[17-19]

$$\Delta G^{\ddagger} = \Delta G_0^{\ddagger}\left(1 + \frac{\Delta G_{\mathrm{ET}}^{\mathrm{o}} - D_{\mathrm{p}}}{4\Delta G_0^{\ddagger}}\right)^2 \quad (5.3)$$

$$\Delta G_0^{\ddagger} = \frac{\left(\sqrt{\mathrm{BDE_{RX}}} - \sqrt{D_{\mathrm{p}}}\right)^2 + \lambda}{4} \quad (5.4)$$

with

$$\lambda = \lambda_{\mathrm{o}} + \lambda_{\mathrm{i}}^{\mathrm{PDI}} \quad (5.5)$$

where $\Delta G_{\mathrm{ET}}^{\mathrm{o}}$ is the reaction standard free energy, $\Delta G_0^{\ddagger}$ is the intrinsic barrier (*i.e.* the activation free energy when $\Delta G_{\mathrm{ET}}^{\mathrm{o}}$ = 0) of the reaction, λ_o is the solvent reorganization energy, BDE_{RX} is the R—X bond dissociation energy, D_p is the interaction energy between $R^•$ and X^- in the solvent cage, and λ_i^{PDI} is the internal reorganization energy contribution relative to the transformation $^*PDI^{•-}$→PDI (extracted from the PDI/$PDI^{•-}$ reorganization energy value calculated as described in section 5.3).

Relevant thermodynamic, kinetic and geometric parameters used in the calculations are listed in Table S1. An example calculation of the k_{SET} between $*PDI^{\bullet-}$ and MBiB is reported in the following pages. The temperature used for the calculations was that estimated as local temperature at the electrode, ca. 50 °C.

Table S1. Thermodynamic, kinetic and geometric parameters for the calculation of $k_{SET,th}$.

Catalyst	RX	r_{RX}[a] (Å)	r[b] (Å)	λ_o (kJ mol^{-1})	λ (kJ mol^{-1})	BDE_{RX}[d] (kJ mol^{-1})	ΔG^0_{ET} (kJ mol^{-1})	$\Delta G^{\ddagger}_0$ (kJ mol^{-1})	D_p (kJ mol^{-1})	E^0_{RX} vs SCE (V)
$*PDI^{\bullet-}$	MBiB	3.72	2.89	59.51	78.39[c]	258.4	−130.3	72.8	2.22[d]	−0.52[e]
$*PDI^{\bullet-}$	EBiB	3.87	2.93	58.67	77.55[c]	259.0	−136.0	72.7	2.22[d]	−0.46[e]
$*PDI^{\bullet-}$	BrACN	3.02	2.65	64.77	83.64[c]	246.3	−133.1	76.2	0.675[f]	−0.49[e]

[a] From Eq. (5.7). [b] Calculated as in Eq. (5.8). [c] $\lambda = \lambda_o + \lambda_i^{PDI}$, with λ_i^{PDI}= 18.87 kJ mol^{-1} (from the data obtained in section 5.3).[d] From reference [20]. D_p values were considered the same for MBiB and EBiB due to the structural similarity between the two. [e] From reference [21]. [f] From reference [14].

i) Determination of solvent reorganization energy

The solvent reorganization energy λ_o was determined by using a semi-empirical equation obtained on the basis of an extensive set of experimental data that relates the reactants radii to λ_o:[23-24]

$$\lambda_o = A\left(\frac{1}{2r_{PDI}} + \frac{1}{2r} - \frac{1}{r_{PDI} + r}\right) \tag{5.6}$$

where A = 414 kJ mol^{-1} Å[25] and r is the hard-sphere radius of RX. For the radius of PDI a value of r_{PDI} = 6.41 Å was obtained from crystallographic data.[26] Values of the hard sphere radii of the alkyl halides were calculated as:

$$r_{RX} = \frac{3}{4}\left(\frac{M_{RX}}{N_A\,\rho\pi}\right)^{\frac{1}{3}} \tag{5.7}$$

and

$$r = \frac{(2r_{RX} - r_X)\cdot r_X}{r_{RX}} \tag{5.8}$$

using r_{Cl} = 1.81 Å, r_{Br} = 1.96 Å, r_I = 2.20 Å.[27] In the case of MBiB the above equations become:

$$r_{RX} = \frac{3}{4}\left(\frac{0.181\ \text{kg mol}^{-1}}{N_A\cdot 1399\ \text{kg m}^{-3}\cdot\pi}\right)^{\frac{1}{3}} = 3.716\cdot 10^{-10}\ \text{m}$$

$$r = \frac{(2\cdot 3.716\ \text{Å} - 1.96\ \text{Å})\cdot 1.96\ \text{Å}}{3.716\ \text{Å}} = 2.886\ \text{Å}$$

and

$$\lambda_o = 414 \text{ kJ mol}^{-1}\text{Å}\left(\frac{1}{2{\cdot}6.41 \text{ Å}}+\frac{1}{2 \cdot 6.41 \text{ Å}}-\frac{1}{6.41 \text{ Å} + 2.886 \text{ Å}}\right) = 59.51 \text{ kJ mol}^{-1}$$

ii) Determination of homogenous collision frequency

The collision frequency factor Z was calculated as:

$$Z = N_A\sqrt{\frac{8\pi RT}{\mu}}(r_{PDI} + r_{RX})^2 \quad (5.9)$$

In this case M_{PDI} = 710.87 g mol^{-1}, $M_{RX} = M_{MBiB}$ = 181.03 g mol^{-1} and the reduced mass μ = 144.29 g mol^{-1}. Therefore, Z= 4.22·10^{11} M^{-1}s^{-1}

iii) Determination of ΔG^{o}_{ET}

Reaction free energy was determined using the following equation[28]

$$\Delta G^{o}_{ET} = F(E^{o}_{PDI/{*}PDI^{\bullet-}} - E^{o}_{RX/R^{\bullet}+X^{-}}) \quad (5.10)$$

where $E^{o}_{PDI/{*}PDI^{\bullet-}}$ and $E^{o}_{RX/R^{\bullet}+X^{-}}$ are taken from the literature (Table S1).

For MBiB

$$\Delta G^{o}_{ET} = 96485 \text{ C mol}^{-1}(-1.87 + 0.52) \text{ V} = -130.3 \text{ kJ mol}^{-1}$$

iv) Determination of $\Delta G^{\ddagger}_{0}$ and $\Delta G^{\ddagger}$

Applying equations (5.3) and (5.4) to the case of MBiB:

$$\Delta G^{\ddagger}_{0} = \frac{\left(\sqrt{BDE_{RX}} - \sqrt{D_p}\right)^2+\lambda}{4} = \frac{\left(\sqrt{258.40} - \sqrt{2.20}\right)^2+78.39}{4} \text{kJ mol}^{-1} = 72.83 \text{ kJ mol}^{-1}$$

$$\lambda = \lambda_o + \lambda_i^{PDI} = 59.51 \text{ kJ mol}^{-1} + \frac{37.75}{2} \text{ kJ mol}^{-1} = 78.39 \text{ kJ mol}^{-1}$$

$$\Delta G^{\ddagger} = \Delta G^{\ddagger}_{0}\left(1 + \frac{\Delta G^{o}_{ET}}{4\Delta G^{\ddagger}_{0}}\right)^2 = 21.65 \text{ kJ mol}^{-1}$$

v) Determination of $k_{SET,th}$

For the reaction between *PDI$^{\bullet-}$ and MBiB we obtain:

$$k_{SET,th} = 4.21 \cdot 10^{11} \text{ M}^{-1}\text{s}^{-1} \exp\left(-\frac{18180 \text{ J mol}^{-1}}{(8.314 \text{ J K}^{-1}\text{mol}^{-1})(323 \text{ K})}\right) = 4.82 \cdot 10^{8} \text{ M}^{-1} \text{ s}^{-1} \quad (5.11)$$

The predicted $k_{SET,th}$ values for all the tested substrates are reported in Table 3 (main text).

5.2 Marcus plot for stepwise dissociative electron transfer: *PDI^{2-} reactivity with aryl halides

The reactivity of *PDI^{2-} with aryl halides was modeled using electron transfer theories. In aryl halides, the halogen is attached to an aromatic system, enabling the electron to occupy a low energy π orbital. This leads

to a stepwise dissociative electron transfer (SDET) mechanism, where an intermediate radical anion forms before bond cleavage.

$$*PDI^{2-} + RX \rightarrow PDI^{\bullet-} + RX^{\bullet-} \tag{5.12}$$

Bond cleavage can then occur:

$$RX^{\bullet-} \rightarrow R^{\bullet} + X^{-} \tag{5.13}$$

For Eq. (5.12), the value of $\Delta G^{\ddagger}$ for was expressed using the Marcus-Agmon-Levine relationship:[29-30]

$$\Delta G^{\ddagger} = \Delta G^{o}_{ET} + \frac{\Delta G^{\ddagger}_{0}}{\ln 2} \cdot \ln\left[1 + \exp\left(-\ln 2 \frac{\Delta G^{o}_{ET}}{\Delta G^{\ddagger}_{0}}\right)\right] \tag{5.14}$$

and the intrinsic barrier is given by

$$\Delta G^{\ddagger}_{0} = \frac{\lambda}{4} = \frac{\lambda_{i} + \lambda_{o}}{4} \tag{5.15}$$

where λ_i is the internal reorganization energy. Thus, experimental values of k_{SET} can be fitted according to the following equation:[12,31]

$$k_{SET} = \frac{k_{diff}}{1 + \frac{k_{diff}}{K_{A} \cdot Z} \cdot \exp\left(\frac{\Delta G^{o}_{ET} + \frac{\Delta G^{\ddagger}_{0}}{\ln 2} \cdot \ln\left[1 + \exp\left(-\ln 2 \cdot \frac{\Delta G^{o}_{ET}}{\Delta G^{\ddagger}_{0}}\right)\right]}{RT}\right) + \exp\left(\frac{\Delta G^{o}_{ET}}{RT}\right)} \tag{5.16}$$

where k_{SET} is the electron transfer rate constant for the aryl halides obtained with the voltammetric e-PRC method, k_{diff} is the diffusion-limited rate constant, K_A is the constant for the formation of the encounter complex [$*PDI^{2-}$---RX], and Z is the collision frequency.

The value of k_{diff} was calculated as:[32]

$$k_{diff} = 4\pi N_{A}\sigma(D_{1} + D_{2}) = 1.46 \cdot 10^{10}\ M^{-1}s^{-1} \tag{5.17}$$

where σ is the separation distance (calculated as the sum of the spherical radii of species 1 and 2). Averaging the values of σ and D across the entire set of RXs yielded $k_{diff} = 1.46 \cdot 10^{10}\ M^{-1}\ s^{-1}$.

ΔG^{o}_{ET} is the driving force of the reaction given by:

$$\Delta G^{o}_{ET} = F\left(E^{o}_{PDI^{\bullet-}/*PDI^{2-}} - E^{o}_{RX/RX^{\bullet-}}\right) + w_{p} - w_{r} \tag{5.18}$$

where F is the faraday constant, $E^{o}_{PDI^{\bullet-}/*PDI^{2-}}$ is the standard reduction potential of $PDI^{\bullet-}$ to the singlet excited state of the dianion $*PDI^{2-}$ and $E^{o}_{RX/RX^{\bullet-}}$ the standard reduction potential of the aryl halide. The

terms w_r and w_p are work terms related to bringing reactants and products together, respectively. They are calculated as:

$$w_i = \frac{N_A q_1 q_2 e^2}{4\pi\varepsilon_0\varepsilon_r\sigma} \tag{5.19}$$

where e is the absolute electron charge, q_i is the charge of the ith species, σ is the minimum distance between reactants calculated as the sum of hard sphere radii ($\sigma = r_{PDI} + r_{RX}$) and ε_r is the dielectric constant of the medium (ε_r (323 K) = 33.25 for *N,N*-dimethylformamide). The radii of aromatic halides were calculated according to equation (5.7).

For the fit shown in Figure 7d the following values were used: $k_{diff} = 1.46 \cdot 10^{10}$ M^{-1} s^{-1}, $Z = 4.2 \cdot 10^{11}$ M^{-1} s^{-1}, $K_A = 4/3N_A\pi(r_{RX} + r_{PDI})^3 = 2.47$ M^{-1},[33] $w_p = 4.2$ kJ mol^{-1}. All these values were averages for the investigated RX series (see Table S2). A value of $E^0_{PDI^{\bullet-}/*PDI^{2-}} = -2.60$ V vs SCE was used. Holding k_{diff}, K_A, Z and $E^0_{*PDI^{2-}/PDI^{\bullet-}}$ values constant, the value of reorganization energy λ ($\lambda = 4\Delta G_0^{\ddagger}$) in Eq. (5.16) was optimized using the Levenberg–Marquardt algorithm. The procedure identified a value of $\lambda = (117 \pm 25)$ kJ mol^{-1}. Both λ_o and λ_i, which are extensively discussed in section 5.3, are significant for the reactions between *PDI^{2-} and aromatic halides.

Table S2. Geometric, thermodynamic and kinetic data relative to RX species used in the probing of *PDI^{2-} reactivity.

Catalyst	RX	E^0_{RX} [a] (V vs SCE)	σ [b] (Å)	Z [c] (M^{-1} s^{-1})	K_A [d] (M^{-1})	w_p [e] (kJ mol^{-1})
*PDI^{2-}	4-BAP	−1.84	10.0	$4.00 \cdot 10^{11}$	2.55	4.16
*PDI^{2-}	4-MCB	−2.02	10.1	$4.27 \cdot 10^{11}$	2.57	4.15
*PDI^{2-}	4-CBN	−2.03	9.9	$4.52 \cdot 10^{11}$	2.45	4.22
*PDI^{2-}	1-BrNaph	−2.17	10.2	$4.08 \cdot 10^{11}$	2.69	4.09
*PDI^{2-}	PhI	−2.24	9.78	$4.16 \cdot 10^{11}$	2.36	4.27
*PDI^{2-}	2-BrPy	−2.26	9.95	$3.89 \cdot 10^{11}$	2.48	4.20
*PDI^{2-}	2-ClPy	−2.37	9.77	$4.15 \cdot 10^{11}$	2.35	4.28
*PDI^{2-}	PhBr	−2.42	9.76	$4.76 \cdot 10^{11}$	2.34	4.28

[a] From ref [22]. [b] $r_{PDI} = 6.41$ Å;[26] r_{RX} calculated with equation (5.7). [c] Calculated with equation (5.9). [d] $K_A = 4/3N_A\pi(r_{RX} + r_{PDI})^3$. Average K_A, Z, w_p, and k_{diff} were used in the fitting. [e] Calculated with equation (5.19)

5.3 Estimation of the reorganization energy of the PDI/PDI$^{\bullet-}$ couple

The reorganization energy (λ) for an electron transfer reaction between an electron donating molecule (D) and an acceptor (A) can be described by the half-sum of self-exchange reorganization energy terms λ.[34-35]

$$\lambda = \frac{\lambda_D + \lambda_A}{2} \quad (5.20)$$

where λ_D is the reorganization energy of the self-exchange reaction in solution, for example:

$$D + D^{\bullet-} \rightleftarrows D^{\bullet-} + D$$

In the case of PDI/$PDI^{\bullet-}$ self-exchange in solution:

$$PDI + PDI^{\bullet-} \rightleftarrows PDI^{\bullet-} + PDI \quad (5.21)$$

we obtain $\lambda = \lambda_{PDI/PDI^{\bullet-}} = 4\Delta G^{\ddagger}_{sol}$. Electrochemical methods are commonly used to estimate the reorganization energy for self-exchange reactions.[23,36] The activation free energy for the self-exchange activation free energy in solution (PDI + $PDI^{\bullet-} \rightleftarrows PDI^{\bullet-}$ + PDI; $\Delta G^{\ddagger}_{sol}$) can be related to the activation energy of the electrode reaction (PDI + e $\rightleftarrows$ $PDI^{\bullet-}$; $G^{\ddagger}_{el}$) via the Hush ($\Delta G^{\ddagger}_{el} = \Delta G^{\ddagger}_{sol}$)[37] or Marcus ($\Delta G^{\ddagger}_{el} = \frac{1}{2}\Delta G^{\ddagger}_{sol}$)[38] theories. Kojima and Bard found that the Hush model provides a more accurate description for aromatic organic molecules.[23]

The $\Delta G^{\ddagger}_{el}$ for the reaction at the electrode was determined from the following equation:

$$\Delta G^{\ddagger}_{el} = -RT\ln\left(\frac{k^0_{corr}}{Z_{el}}\right) \quad (5.22)$$

where Z_{el} is the heterogeneous collision frequency factor and k^0_{corr} is the heterogenous standard rate constant for the electron transfer at the electrode (k^0) corrected for the outer Helmholtz plane (OHP) potential (ϕ_2). Z_{el} was calculated as

$$Z_{el} = \left(\frac{RT}{2\pi M}\right)^{1/2} \quad (5.23)$$

where M is the molar mass of PDI. k^0_{corr} was calculated as:

$$\ln(k^0_{corr}) = \ln(k^0) - \frac{(\alpha - z)F}{RT}\phi_2 \quad (5.24)$$

where α is the charge transfer coefficient that was set equal to 0.5 and z is the charge of the electroactive species (in this case z = 0). The Frumkin correction (ϕ_2) for the glassy carbon working electrode was calculated according to the work of Maran and co-workers.[39] All parameters used for the calculation are listed in Table S3.

Table S3. Parameters used for the calculation of $\lambda_{PDI/PDI^{\bullet-}}$

Couple	k^0 (cm s^{-1})	k^0_{corr}	Z_{el} [a] (cm s^{-1})	$\Delta G^{\ddagger}_{el}$	$\Delta G^{\ddagger}_{sol}$	λ (kJ mol^{-1})	λ_i [b] (kJ mol^{-1})

		(cm s^{-1})		(kJ mol^{-1})	(kJ mol^{-1})					
					Hush	Marcus	Hush	Marcus	Hush	Marcus
PDI/PDI$^{\bullet-}$	0.019	0.261	2330	22.00	22.0	44.1	88.2	176.5	37.7	126.0

[a] From equation (5.23) using M_{PDI} = 710.87 g mol^{-1}. [b] Using λ_o = 50.49 kJ mol^{-1}.

Using the Hush model ($\Delta G^{\ddagger}_{\mathrm{el}} = \Delta G^{\ddagger}_{\mathrm{sol}}$), for the self-exchange reaction, where $\lambda = 4\Delta G^{\ddagger}_{\mathrm{sol}}$, we obtained $\lambda_{\mathrm{PDI/PDI^{\bullet-}}}$ = 88.2 kJ mol^{-1}.

The reorganization energy λ is the sum of internal (λ_i) and solvent reorganization (λ_o), with λ_o calculated as 50.49 kJ mol^{-1} from eq. 5.6. Thus, for the self-exchange reaction of PDI/PDI$^{\bullet-}$ we obtained $\lambda_i = \lambda - \lambda_o$ = 37.7 kJ mol^{-1}.

Experimental estimation of the reorganization energy for the PDI$^{\bullet-}$/PDI^{2-} was unattainable because the Frumkin correction is based on the Gouy–Chapman–Stern (GCS) model, which involves average potentials in the vicinity of the electrode and ignores the discrete nature of charges in solution,[40-41] leading to an inaccurate determination of parameters. Nevertheless, the internal reorganization energy for the PDI$^{\bullet-}$/PDI^{2-} couple is similar to or higher than that of the PDI/PDI$^{\bullet-}$ couple given the change in aromaticity observed upon reduction from PDI$^{\bullet-}$ to PDI^{2-}.[42] The reorganization for PDI$^{\bullet-}$/*PDI^{2-} is likely similar to or higher than that of the ground-state molecules. DFT calculations also indicate substantial reorganization energy for the *PDI^{2-} → PDI$^{\bullet-}$ reduction (see next paragraph).

6 Computational results for the reorganization energy of $^*PDI^{2-}$ to $PDI^{\bullet-}$

The outer-sphere electron transfer from the excited state S_1 of PDI^{2-} ($^*PDI^{2-}$) to $PDI^{\bullet-}$ is characterized by a strong internal reorganization energy λ_i. A state-of-the-art Density Functional Theory (DFT) approach[6,42-43] was employed to rationalize the experimental finding (level of theory: B3LYP/6-31G(d,p)). First, the excited state of interest was identified as the one associated with the first transition of the computed spectrum by computing the vertical transitions in the gas phase as well as in DMF; they both show a blue shift compared to the experimental spectrum (Figure S6), which is smaller when the solvent is included.

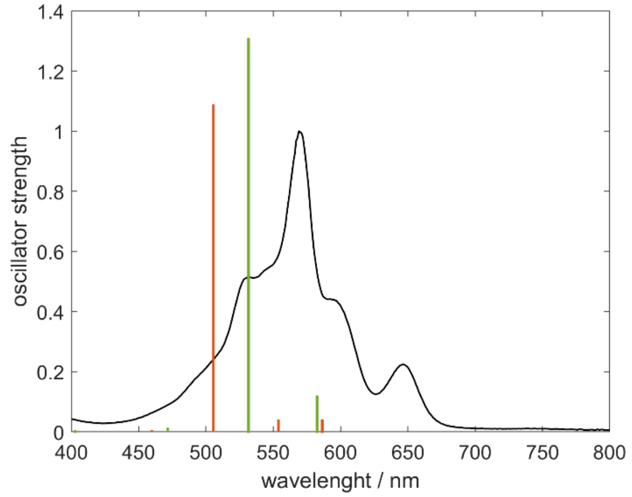

Figure S6. Computed excitations of PDI^{2-} with corresponding oscillator strength in the gas phase (red) and in DMF (green). The experimental absorption spectrum with normalized absorbance is shown in black. Level of theory: (SMD)-TD-B3LYP/6-31G(d,p)//B3LYP/6-31G(d,p).

The qualitative comparison between the experimental and computed absorptions allowed to associate with confidence the lowest excitation to the S_1 state of PDI^{2-} from which the electron transfer occurs. Moreover, since this transition energy does not change significantly by including the implicit solvation, the analysis was performed in the gas phase. The Natural Transition Orbitals (NTOs) involved in the lowest transition are shown in Figure S6. The electron NTO is a π orbital of the central aromatic system, while the hole NTO is still a π system with additional lobes on the two peripheric aromatic rings, suggesting an accumulation of electron density in these external moieties in the excited state.

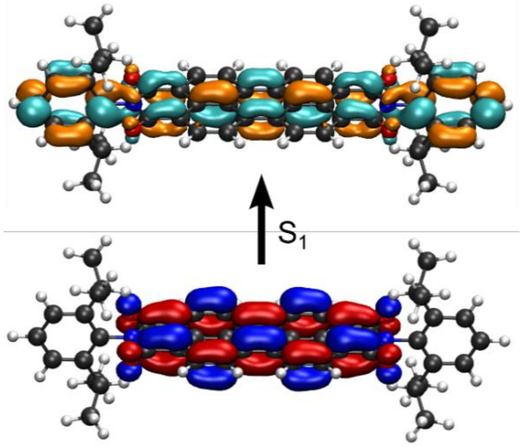

Figure S7. NTOs for the lowest electronic transition; hole (cyan/orange) and electron (blue/red). Isosurface value of 0.03. Level of theory: B3LYP/6-31G(d,p).

Once the excited state was identified, a value of 6.0 kcal mol^{-1} (25 kJ mol^{-1}) was calculated as the internal reorganization energy λ_i; indeed, this value is not negligible to accurately describe the total reorganization energy of the process. Particularly, λ_i is determined by a structural difference between the optimized geometries of *PDI^{2-} and PDI$^{\bullet-}$ involving the external aromatic rings bonded to the imide nitrogen atoms. As NTOs indicate, electron density is accumulated on these rings causing a variation of dihedral angles and C—C bond lengths (Figure S8) in one of them, thus slightly breaking the molecular symmetry. In detail, PDI$^{\bullet-}$ is symmetric; thus, d_1, d_1' and θ_1 are identical to d_2, d_2' and θ_2, respectively. Conversely, in *PDI^{2-}, the ring 1 remains unperturbed in the distances d_1 and d_1', but θ_1 increases by 3°. Meanwhile, d_2 increases by 0.04 Å, d_2' decreases by 0.02 Å and θ_2 only decreases by 1°. Consequently, the isopropyl groups are moderately rotated in the excited state. Overall, the high value of λ_i can be ascribed to the C-C bond stretching and the symmetry loss; particularly, bond elongations are computed in correspondence of orbital nodes in the hole NTO. Indeed, the computed λ_i for the electron transfer from the ground state of PDI$^{\bullet-}$/PDI^{2-} (3.5 kcal mol^{-1}, i.e., 14 kJ mol^{-1}) is smaller than the one relative to the excited state and in this case only a slight symmetric rotation (3°) of the dihedral angles θ_1 and θ_2 with no variation of the bond lengths is computed.

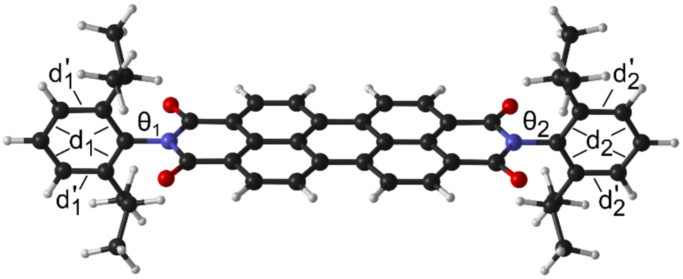

Figure S8. Fully optimized structure of PDI$^{\bullet-}$. Main geometrical parameters (dihedral angles θ and distances d) which differs from *PDI^{2-} are visualized. Level of theory: B3LYP/6-31G(d,p).

7 Full mechanism for CV simulations

7.1 Reactivity of $^*PDI^{\bullet-}$

The reactions used as input for the CV simulation software DigiElch are represented in Figure 3a in the main text and listed in Table S4.

Table S4. Reactions and relative rate constants for the determination of k_{SET} for the reaction $^*PDI^{\bullet-}$ with MBiB, EBiB, BrACN, 4-IBA and 4-MCB.

Reaction	Rate Constant	Value
$^*PDI^{\bullet-} + RX \rightarrow PDI + R^{\bullet} + X^{-}$	k_{SET} (M^{-1} s^{-1})	*Fitted (see Table 3)*
$PDI + e^{-} \rightleftarrows PDI^{\bullet-}$	k^0 (cm s^{-1})	0.019
$PDI^{\bullet-} + h\nu \rightarrow {}^*PDI^{\bullet-}$	k_{exc} (s^{-1})	73.6
$^*PDI^{\bullet-} \rightarrow PDI^{\bullet-}$	k_d (s^{-1})	$6.25 \cdot 10^9$
$PDI^{\bullet-} + R^{\bullet} \rightarrow PDI\text{-}R^{-}$	k_c (M^{-1} s^{-1})	10^9
$R^{\bullet} + R^{\bullet} \rightarrow RR$	k'_c (M^{-1} s^{-1})	10^9
$PDI^{\bullet-} + R^{\bullet} \rightleftarrows PDI + R^{-}$	k_{red} (M^{-1} s^{-1})	[a]

[a] The fitting procedure yielded similar results irrespective of the value of k_{red}. The recorded current where too low to discriminate between the impact of radical coupling or reduction in the fate of $PDI^{\bullet-}$.

The heterogeneous rate constant for the electron transfer (k^0) was determined using the Nicholson method (Figure S3). The rate coefficient of $^*PDI^{\bullet-}$ excited state formation (k_{exc}) was determined via Eq. (6) in the main text. The decay rate of $^*PDI^{\bullet-}$, k_d, was determined as the reciprocal of its lifetime. The rate constant of the coupling between $PDI^{\bullet-}$ and $R^{\bullet}$ (k_c) was set to 10^9 M^{-1} s^{-1} (this value was reported for the coupling between several alkyl radicals and aromatic radical anions[44-45]). Biradical coupling between two $R^{\bullet}$ was also set to 10^9 M^{-1} s^{-1} (this reaction had minimal effect on the CV simulation). The values of the diffusion coefficients of RXs were estimated from the literature;[46] changing the diffusion coefficient in the range ±50% from these values had no effect on the simulated CVs.

7.2 Additional considerations on the electrophotocatalysis with PDI

In addition to the reactions in Figures 3a and 6a, additional pathways were evaluated. All these reactions were found to have a negligible effect on the simulation. Reported below is the complete list of reactions considered in the CV simulation, along with relevant considerations.

(i) Back electron transfers (BET)

The excited state catalyst $*PDI^{\bullet-}$ can undergo heterogenous BET at the electrode ($k_{BET,het}$) and homogenous BET with its ground state PDI ($k_{BET,hom}$) according to the following reactions.

$$*PDI^{\bullet-} \rightarrow PDI + e^- \qquad k_{BET,het}\ (cm\ s^{-1}) \qquad (7.1)$$

$$*PDI^{\bullet-} + PDI \rightarrow PDI + PDI^{\bullet-} \qquad k_{BET,hom}\ (M^{-1}\ s^{-1}) \qquad (7.2)$$

Both reactions were found to have negligible impact on the simulated CV even when the rate constants were set at very large values ($k_{BET,het} = 10^4$ cm s^{-1} and $k_{BET,hom} = 10^{10}$ M^{-1} s^{-1}).

Heterogeneous BET is highly favored since $E^0_{PDI/*PDI^{\bullet-}}$ is much more negative than $E^0_{PDI/PDI^{\bullet-}}$. However, the concentration of $*PDI^{\bullet-}$ at the electrode surface is effectively insignificant,[48] which makes heterogeneous BET negligible.

(ii) Reactions of alkyl radicals with $*PDI^{\bullet-}$

The rate constants (k_{red*}) for radical ($R^\bullet$) reduction by $*PDI^{\bullet-}$ are likely very high and were set to the diffusion-controlled limit. However, digital simulation confirmed its negligible contribution due to the very low concentrations of both $*PDI^{\bullet-}$ and $R^\bullet$.

$$*PDI^{\bullet-} + R^\bullet \rightarrow PDI + R^- \qquad k_{red*}\ (M^{-1}\ s^{-1}) \qquad 10^9 \qquad (7.3)$$

It should be noted that no reactions potentially occurring within the solvent cage were considered.

(iii) Coupling reactions between alkyl radicals

$$R^\bullet + R^\bullet \rightarrow RR \qquad k'_c\ (M^{-1}\ s^{-1}) \qquad 10^9 \qquad (7.4)$$

As previously noted, the coupling rate constant for organic radicals was set to ~10^9 M^{-1} s^{-1}. Due to low radical concentration, its impact on the simulation was minimal, but the reaction was included for completeness.

7.3 Reactivity of the excited state of the dianion catalyst, $*PDI^{2-}$

In this case for the evaluation of k_{SET}, the simulated mechanism is reported in Figure 6a. The full simulated mechanism with utilized rate constants is here reported in Table S5 and Table S6.

Table S5. Reactions and relative rate constants for the determination of k_{SET}.

Reaction	Rate Constant	Value
$*PDI^{2-} + RX \rightarrow PDI + RX^{\bullet-}$	k_{SET} (M^{-1} s^{-1})	*Fitted,* see Table 3
$PDI + e^{-} \rightarrow PDI^{\bullet-}$	k^0 (cm s^{-1})	0.019
$PDI^{\bullet-} + e^{-} \rightarrow PDI^{2-}$	k^0 (cm s^{-1})	0.020
$PDI^{2-} + h\nu \rightarrow *PDI^{2-}$	k_{exc} (s^{-1})	86.5
$*PDI^{2-} \rightarrow PDI^{2-}$	k_d (s^{-1})	$1.56 \cdot 10^8$
$PDI^{\bullet-} + R^{\bullet} \rightarrow PDI\text{-}R^{-}$	k_c (M^{-1} s^{-1})	10^9
$R^{\bullet} + R^{\bullet} \rightarrow RR$	k_c' (M^{-1} s^{-1})	10^9
$RX^{\bullet-} \rightarrow R^{\bullet} + X^{-}$	k_{fr} (s^{-1})	See Table S6
$PDI^{2-} + R^{\bullet} \rightarrow PDI^{\bullet-} + R^{-}$	k_{red} (M^{-1} s^{-1})	*fitted*, see Table S6

Table S6. Rate coefficients for $RX^{\bullet-}$ fragmentation (k_{fr}) and $R^{\bullet}$ reduction (k_{red}) by $*PDI^{2-}$ calculated for the investigated aryl halides species.

RX	k_{red}[a] (M^{-1} s^{-1})	k_{fr}[b] (s^{-1})
4-MCB	$6.31 \cdot 10^8$	$1.26 \cdot 10^7$
4-BAP	$6.31 \cdot 10^8$	$3.16 \cdot 10^7$
4-CBN	$7.94 \cdot 10^8$	$5.01 \cdot 10^8$
1-BrNaph	$2.66 \cdot 10^8$	$1.00 \cdot 10^9$
PhI	$2.51 \cdot 10^8$	$> k_{diff}$
2-BrPy	$1.58 \cdot 10^8$	$3.16 \cdot 10^9$
2-ClPy	$2.00 \cdot 10^8$	$3.98 \cdot 10^9$
PhBr	$2.00 \cdot 10^8$	$> k_{diff}$

[a] Fitted using the software DigiElch. [b] From references [22,49].

Similarly to the study of $*PDI^{\bullet-}$ reactivity, the following possible side reactions were considered and were found to have no effect on the simulated CVs.

Table S7. Additional reactions that had no effect on the simulations.

$$*PDI^{2-} \rightarrow PDI^{\bullet-} + e^- \qquad k_{BET,het}\ (cm\ s^{-1}) \qquad (7.5)$$

$$*PDI^{2-} + PDI \rightarrow PDI^{\bullet-} + PDI^{\bullet-} \qquad k_{BET,hom}\ (M^{-1}\ s^{-1}) \qquad (7.6)$$

$$*PDI^{2-} + R^{\bullet} \rightarrow PDI^{\bullet-} + R^- \qquad k_{red*}\ (M^{-1}\ s^{-1}) \qquad (7.7)$$

Contrary to $PDI^{\bullet-}$, the reduction of the aryl radical ($R^{\bullet}$) by the dianion PDI^{2-} (k_{red}) had great impact on the simulated CVs and it was fitted by the simulation software. This aligns with the greater reducing power of PDI^{2-} compared to $PDI^{\bullet-}$. The obtained values of k_{red}, in the order of 10^8–10^9 M^{-1} s^{-1}, are listed in Table S6.

Solvent abstraction from aryl radical $R^{\bullet}$ was considered:

$$R^{\bullet} + SH \rightarrow S^{\bullet} + RH \qquad k_{abs}\ (M^{-1}\ s^{-1}) \qquad (7.8)$$

For phenyl-type radicals this reaction typically proceeds with high rate constant (10^6–10^7 M^{-1} s^{-1}).[50] In essence this is a radical transfer reaction from $R^{\bullet}$ to $S^{\bullet}$, which can couple with each other or with $PDI^{\bullet-}$.

$PDI^{\bullet-} + S^{\bullet} \rightarrow PDI\text{-}S^-$	$k_c\ (M^{-1}\ s^{-1})$	10^9
$S^{\bullet} + S^{\bullet} \rightarrow S\text{-}S$	$k_c\ (M^{-1}\ s^{-1})$	10^9
$S^{\bullet} + R^{\bullet} \rightarrow S\text{-}R$	$k_c\ (M^{-1}\ s^{-1})$	10^9

Adding the reactivity of this additional radical had no effect on the fitted value of k_{SET}.

7.4 Evaluating the impact of the rate of C−X bond cleavage

For the reduction of aryl halides, given the stepwise nature of the C—X bond cleavage, we considered the competition between the intramolecular cleavage of the carbon halogen bond (k_{fr}) and the unproductive back electron transfer from $RX^{\bullet-}$ (k_{-ET}). The mechanism for the homogeneous dissociative electron transfer is reported in Scheme S1.

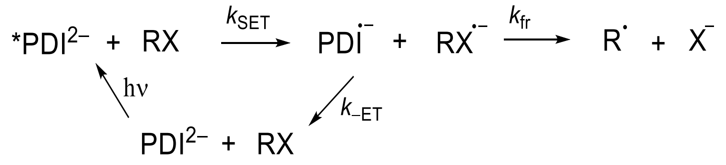

Scheme S1. Reductive stepwise bond cleavage for aryl halides catalyzed by $*PDI^{2-}$.

The bond cleavage efficiency (φ) was evaluated considering the competition between the $RX^{\bullet-}$ fragmentation rate (υ_{fr}) and the rate of the electron transfer between $RX^{\bullet-}$ and $PDI^{\bullet-}$ (υ_{-ET}):

$$\varphi = \frac{\upsilon_{fr}}{\upsilon_{fr} + \upsilon_{-ET}} = \frac{k_{fr}}{k_{fr} + [PDI^{\bullet-}]k_{-ET}} \tag{7.8}$$

The value of φ was evaluated using the reaction with 4-MCB as a reference, as it represented the limiting case with the lowest φ due to its relatively slow cleavage rate ($1.3 \cdot 10^7$ s^{-1})[22], making k_{-ET} most relevant. Due to the reaction's high driving force, k_{-ET} was fixed at the diffusion limit (10^{10} M^{-1} s^{-1}). Under these conditions, 4-MCB gave $\varphi = 0.76$, implying that back electron transfer accounts for the fate of a modest 24% of $RX^{\bullet-}$ intermediate. Therefore, back electron transfer was considered negligible and subsequently neglected for all investigated RX compounds. Figure S9 plots φ vs. k_{fr}.

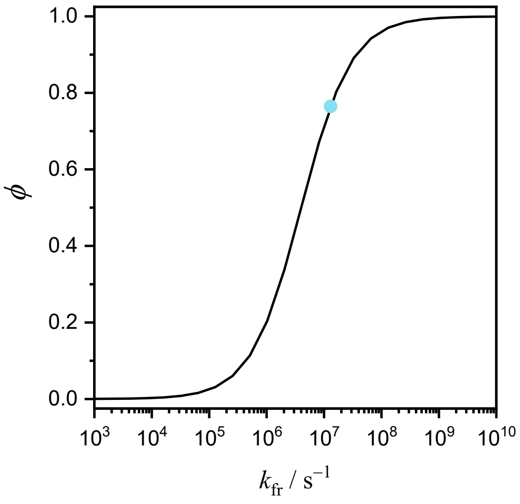

Figure S9. Variation of φ with k_{fr} and calculated value of φ for the radical anion of 4-MCB (blue dot). k_{-ET} was set to the diffusion-limited rate constant of 10^{10} M^{-1} s^{-1} and $[PDI^{\bullet-}]$ was fixed at 10^{-3} M.

7.5 Sensitivity analysis of k_{SET} and k_{red} values obtained by digital simulation

We performed a sensitivity analysis to evaluate how variations in k_{SET} and k_{red} affect the simulated CV current intensities. k_{SET} had the strongest influence: a twofold increase or decrease in k_{SET} led to an average change of about 5% in current—a clearly noticeable effect. Based on this, we estimate the uncertainty in k_{SET} to be within 0.2–0.3 log units.

In contrast, the simulations were much less sensitive to k_{red}: a twofold variation resulted in an average current change of only ~3%. Therefore, the k_{red} values should be considered as rough estimates, with an uncertainty of approximately 0.4–0.5 log units.

8 Experimental and simulated CVs for RX substrates

Voltammetric data for all substrates are here presented. The simulated CVs were fitted in agreement with the mechanisms in Figure 3a (Table S4) for alkyl halides and Figure 6a (Table S5) for aryl halides.

8.1 Ehtyl α-bromoisobutyrate (EBiB)

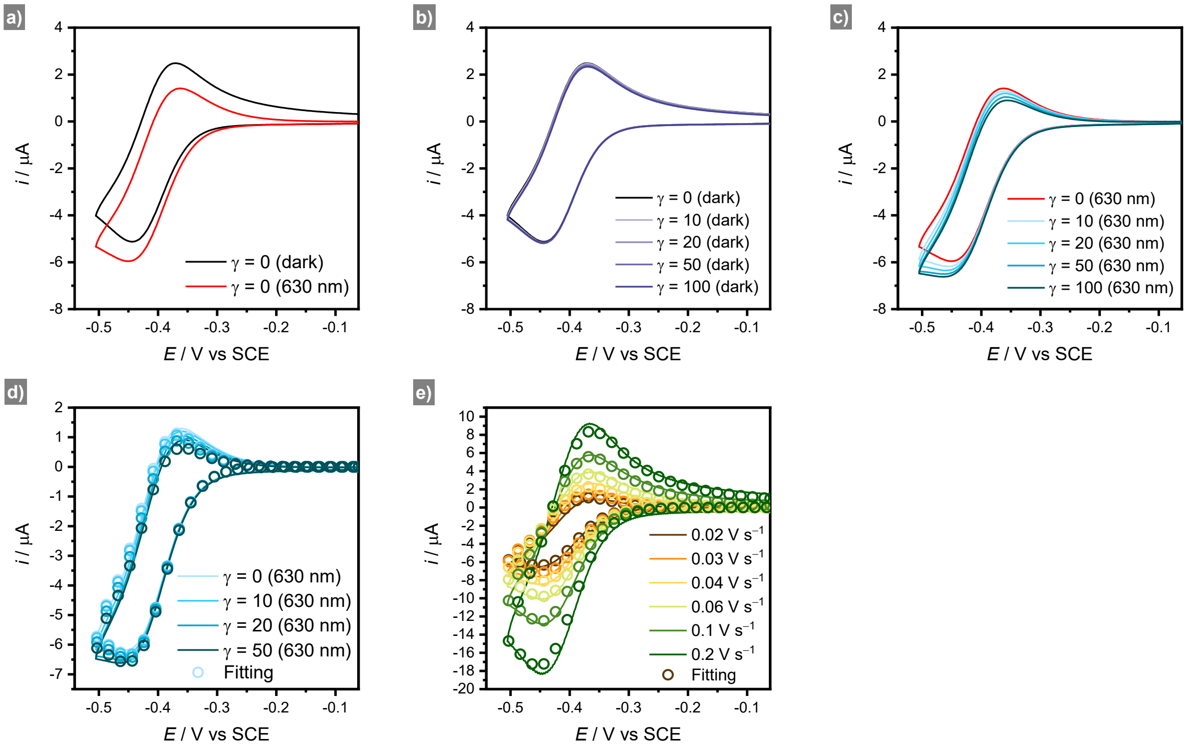

Figure S10. a) CVs of 10^{-3} M PDI in the absence of and under 630 nm light irradiation. $\upsilon = 0.02$ V s^{-1}. b) CVs of 10^{-3} M PDI in the absence and in the presence of EBiB at different concentrations, labeled on the curves ($\gamma = C_{\mathrm{EBiB}}/C_{\mathrm{PDI}}$). $\upsilon = 0.02$ V s^{-1}. c) Experimental CVs recorded at increasing concentration of EBiB. $\upsilon = 0.02$ V s^{-1}. d) Comparison between experimental (lines) and simulated CVs (circles) at different EBiB concentrations at $\upsilon = 0.02$ V s^{-1}. e) Comparison between experimental (lines) and simulated CVs (circles) at 0.1 M EBiB concentration and different scan rates. All the experimental CVs were recorded in DMF + 0.1 M n-Bu_4NBF_4 on a GC electrode (A = 7.07 mm^2) at T = 25 °C.

8.2 Bromoacetonitrile (BrACN)

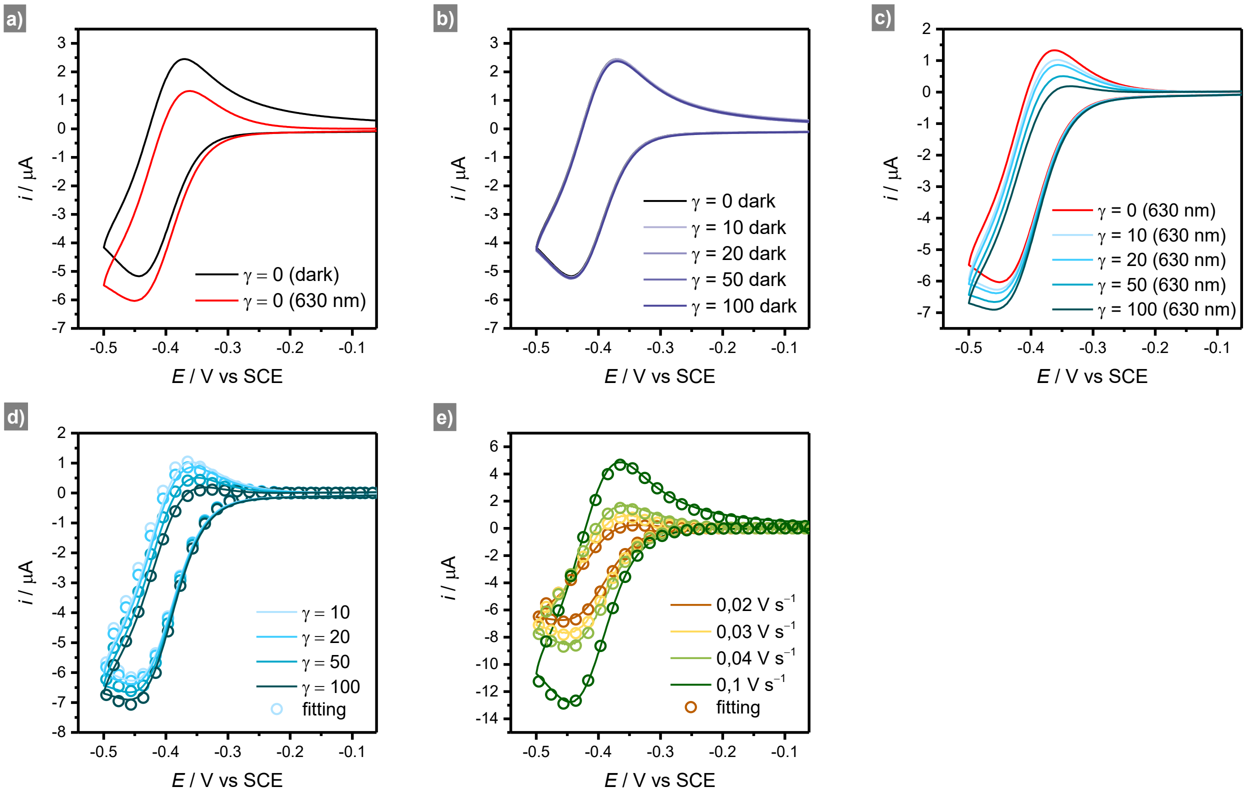

Figure S11. a) CVs of 10^{-3} M PDI in the absence of and under 630 nm light irradiation. $\upsilon = 0.02$ V s^{-1}. b) CVs of 10^{-3} M PDI in the absence and in the presence of BrACN at different concentrations, labeled on the curves ($\gamma = C_{BrACN}/C_{PDI}$). $\upsilon = 0.02$ V s^{-1}. c) Experimental CVs recorded at increasing concentration of BrACN. $\upsilon = 0.02$ V s^{-1}. d) Comparison between experimental (lines) and simulated CVs (circles) at different BrACN concentrations at $\upsilon = 0.02$ V s^{-1}. e) Comparison between experimental (lines) and simulated CVs (circles) at 0.1 M BrACN concentration and different scan rates. All the experimental CVs were recorded in DMF + 0.1 M n-Bu_4NBF_4 on a GC electrode ($A = 7.07$ mm^2) at $T = 25$ °C.

8.3 4’-Iodobenzaldehyde (4-IBA)

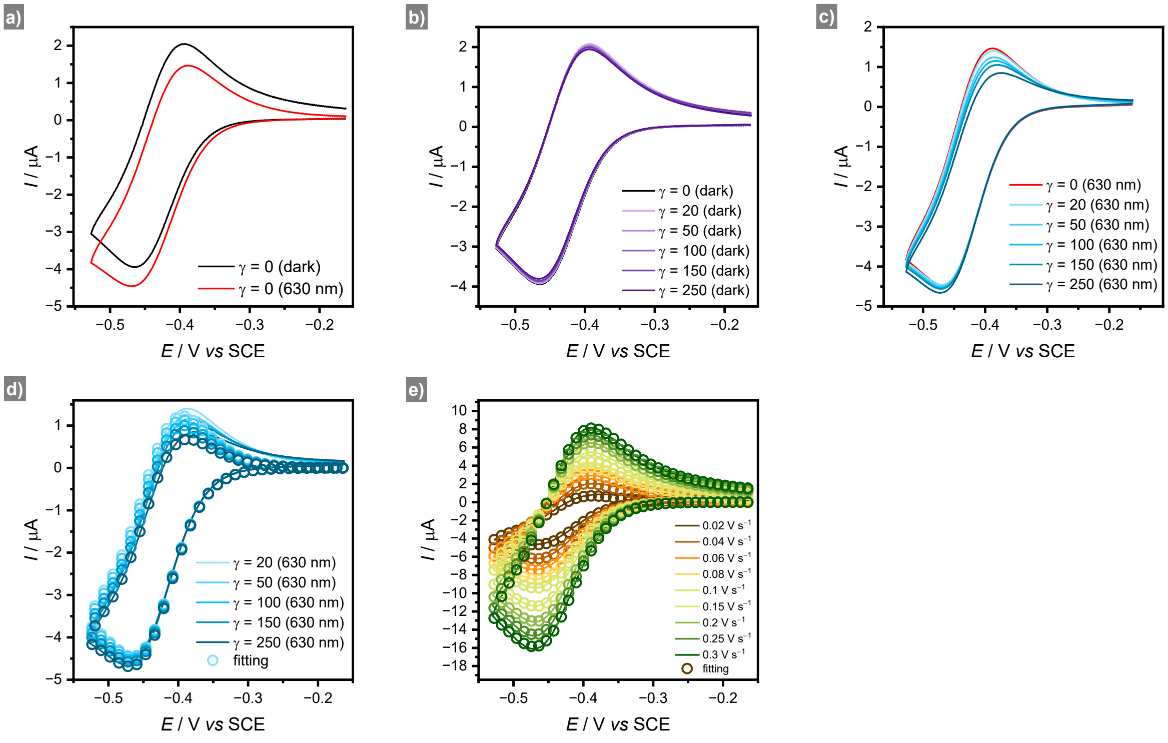

Figure S12. a) CVs of $8 \cdot 10^{-4}$ M PDI in the absence of and under 630 nm light irradiation. $\upsilon = 0.02$ V s^{-1}. b) CVs of 10^{-3} M PDI in the absence and in the presence of 4-IBA at different concentrations, labeled on the curves ($\gamma = C_{4\text{-IBA}}/C_{\text{PDI}}$). $\upsilon = 0.02$ V s^{-1}. c) Experimental CVs recorded at increasing concentration of 4-IBA. $\upsilon = 0.02$ V s^{-1}. d) Comparison between experimental (lines) and simulated CVs (circles) at different 4-IBA concentrations at $\upsilon = 0.02$ V s^{-1}. e) Comparison between experimental (lines) and simulated CVs (circles) at 0.2 M 4-IBA concentration and different scan rates. Currents for $\gamma > 0$ were normalized to the effective concentration of PDI after each addition of 4-IBA to account for dilution. All the experimental CVs were recorded in DMF + 0.1 M n-Bu_4NBF_4 on a GC electrode ($A = 7.07$ mm^2) at $T = 25$ °C.

In this case, since bond cleavage is likely much slower than other electrochemical and photophysical processes, the catalytic current is smaller than expected due to unproductive back electron transfer (Figure 8, main text). Accordingly, the fitting procedure only yields a lower bound for k_{SET}.

4-Bromobenzaldehyde and 4-Nitroanisole

No significant catalytic current was observed when applying the voltammetric e-PRC method to study the catalytic activity of $^{*}PDI^{\bullet-}$ towards 4-bromobenzaldehyde and 4-nitroanisole.

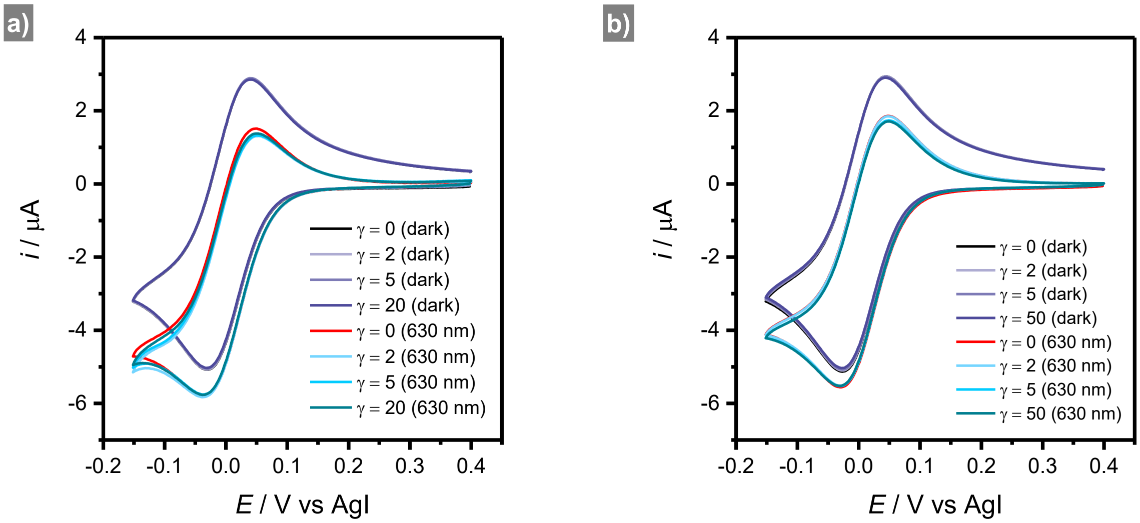

Figure S13. CVs of 10^{-3} M PDI in the absence of and under 630 nm light irradiation, and in the presence and in the absence of a) 4-bromobenzaldehyde and b) 4-nitroanisole at different concentrations labeled on the curves ($\gamma = C_{RX}/C_{PDI}$). All the experimental CVs were recorded in DMF + 0.1 M n-Bu_4NBF_4 on a GC electrode (A = 7.07 mm^2) at T = 25 °C, at a scan rate υ = 0.02 V s^{-1}.

8.4 4′-Bromoacetophenone (4-BAP)

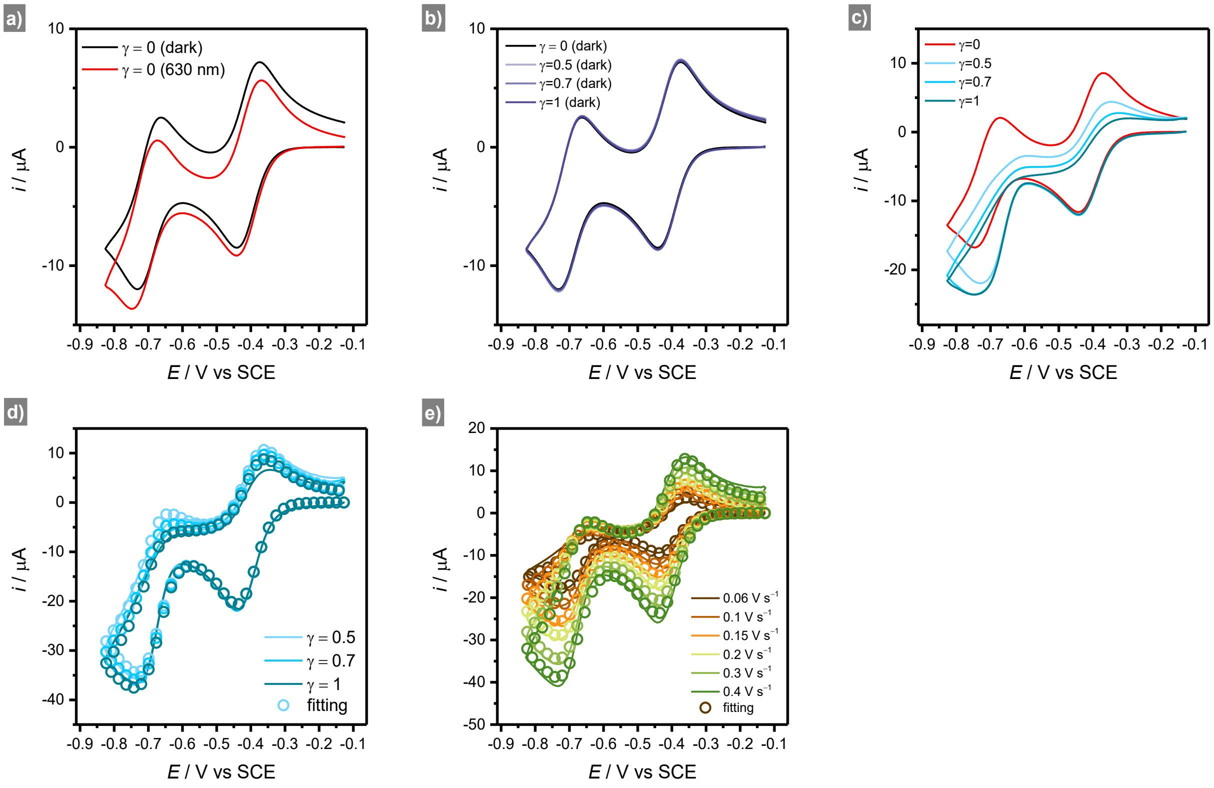

Figure S14. a) CVs of 10^{-3} M PDI in the absence of and under 630 nm light irradiation. $\upsilon = 0.06$ V s^{-1}. b) CVs of 10^{-3} M PDI in the absence and in the presence of 4-BAP at different concentrations, labeled on the curves ($\gamma = C_{4\text{-BAP}}/C_{\text{PDI}}$). $\upsilon = 0.06$ V s^{-1}. c) Experimental CVs recorded at increasing concentration of 4-BAP. $\upsilon = 0.3$ V s^{-1}. d) Comparison between experimental (lines) and simulated CVs (circles) at different 4-BAP concentrations at $\upsilon = 0.3$ V s^{-1}. e) Comparison between experimental (lines) and simulated CVs (circles) at $5 \cdot 10^{-4}$ M 4-BAP concentration and different scan rates. All the experimental CVs were recorded in DMF + 0.1 M n-Bu_4NBF_4 on a GC electrode (A = 7.07 mm^2) at T = 25 °C.

8.5 4-Chlorobenzonitrile (4-CBN)

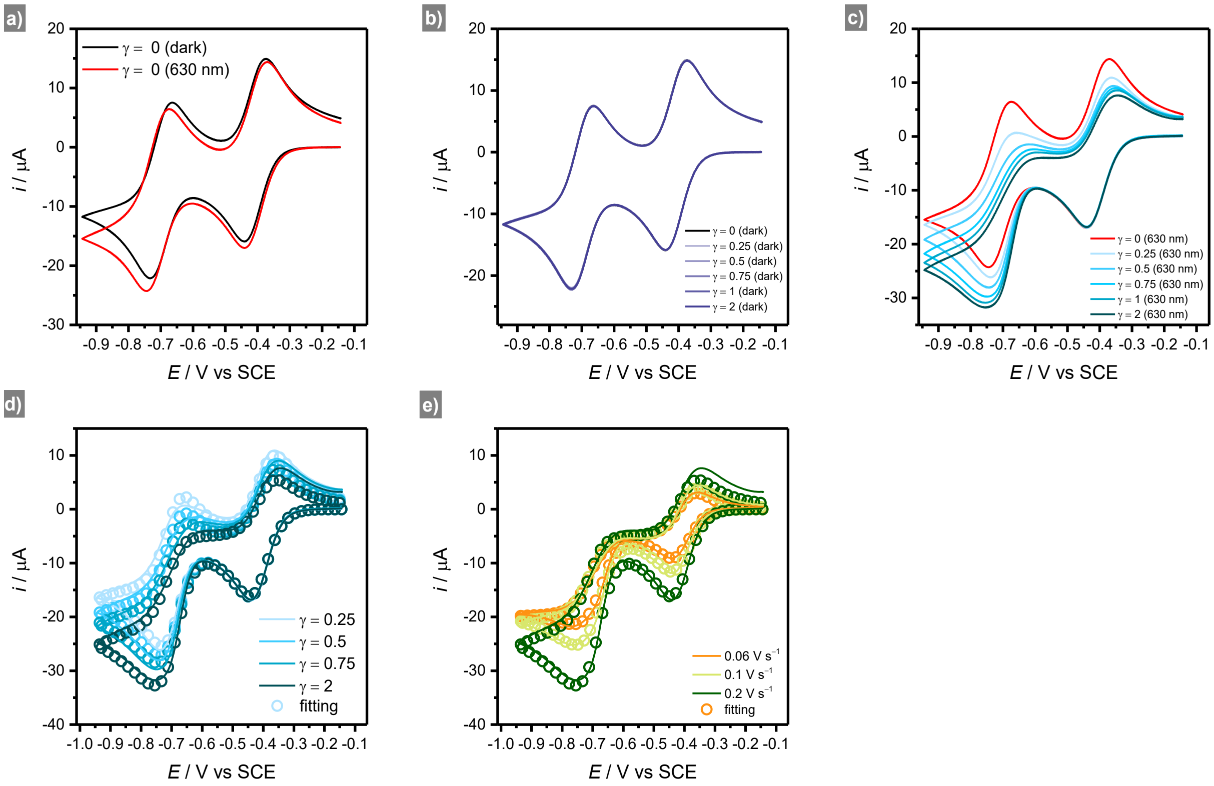

Figure S15. a) CVs of 10^{-3} M PDI in the absence of and under 630 nm light irradiation. $\upsilon = 0.06$ V s^{-1}. b) CVs of 10^{-3} M PDI in the absence and in the presence of 4-CBN at different concentrations, labeled on the curves ($\gamma = C_{4\text{-CBN}}/C_{\text{PDI}}$). $\upsilon = 0.06$ V s^{-1}. c) Experimental CVs recorded at increasing concentration of 4-CBN. $v = 0.2$ V s^{-1}. d) Comparison between experimental (lines) and simulated CVs (circles) at different 4-CBN concentrations at $\upsilon = 0.3$ V s^{-1}. e) Comparison between experimental (lines) and simulated CVs (circles) at $2 \cdot 10^{-3}$ M 4-CBN concentration and different scan rates. All the experimental CVs were recorded in DMF + 0.1 M n-Bu_4NBF_4 on a GC electrode ($A = 7.07$ mm^2) at $T = 25$ °C.

8.6 1-Bromonaphthalene (1-BrNaph)

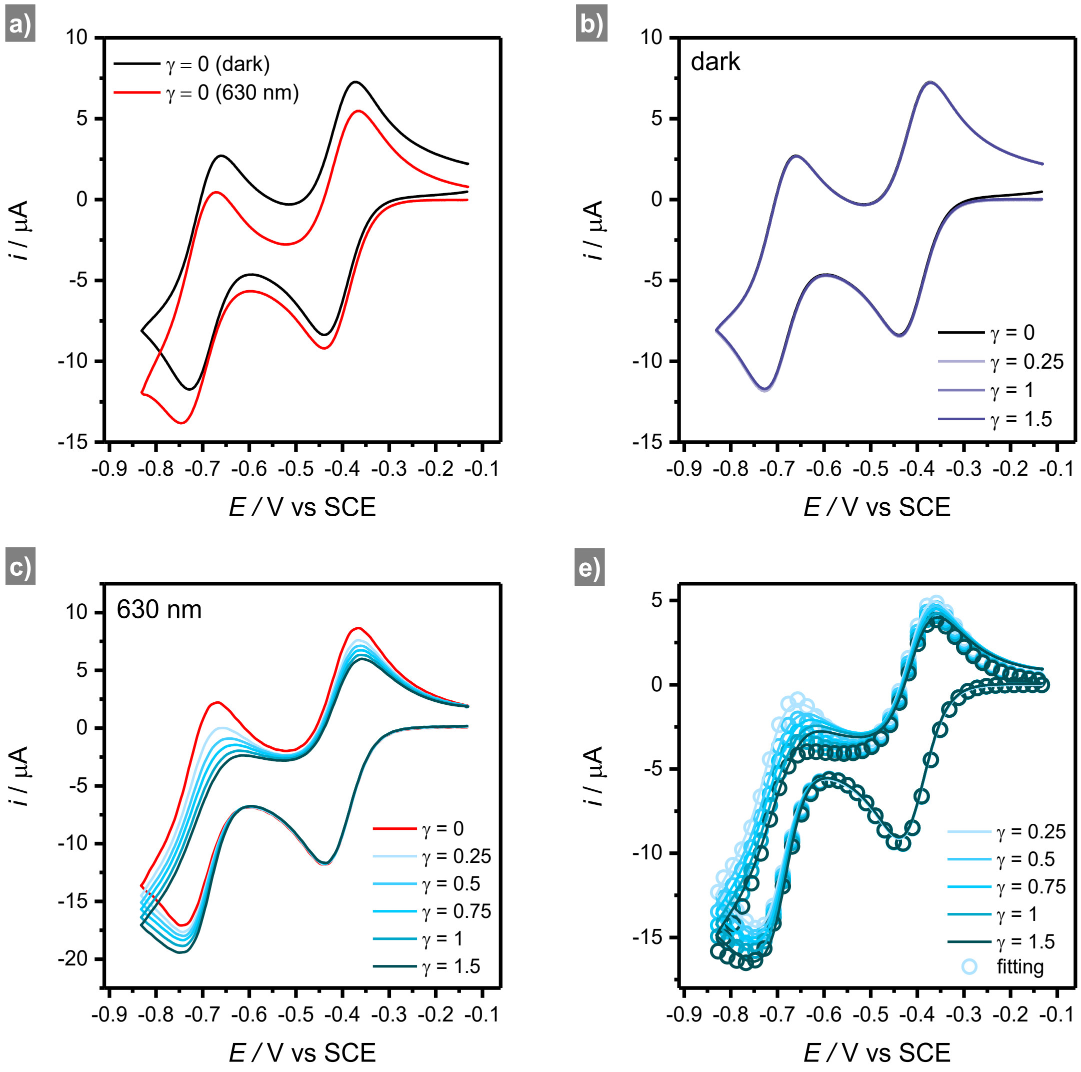

Figure S16. a) CVs of 10^{-3} M PDI in the absence of and under 630 nm light irradiation. $\upsilon = 0.06$ V s^{-1}. b) CVs of 10^{-3} M PDI in the absence and in the presence of 1-BrNaph at different concentrations, labeled on the curves ($\gamma = C_{1\text{-BrNaph}}/C_{\text{PDI}}$). $\upsilon = 0.06$ V s^{-1}. c) Experimental CVs recorded at increasing concentration of 1-BrNaph. $\upsilon = 0.1$ V s^{-1}. d) Comparison between experimental (lines) and simulated CVs (circles) at different 1-BrNaph concentrations at $\upsilon = 0.06$ V s^{-1}. All the experimental CVs were recorded in DMF + 0.1 M n-Bu_4NBF_4 on a GC electrode ($A = 7.07$ mm^2) at $T = 25$ °C.

8.7 Iodobenzene (PhI)

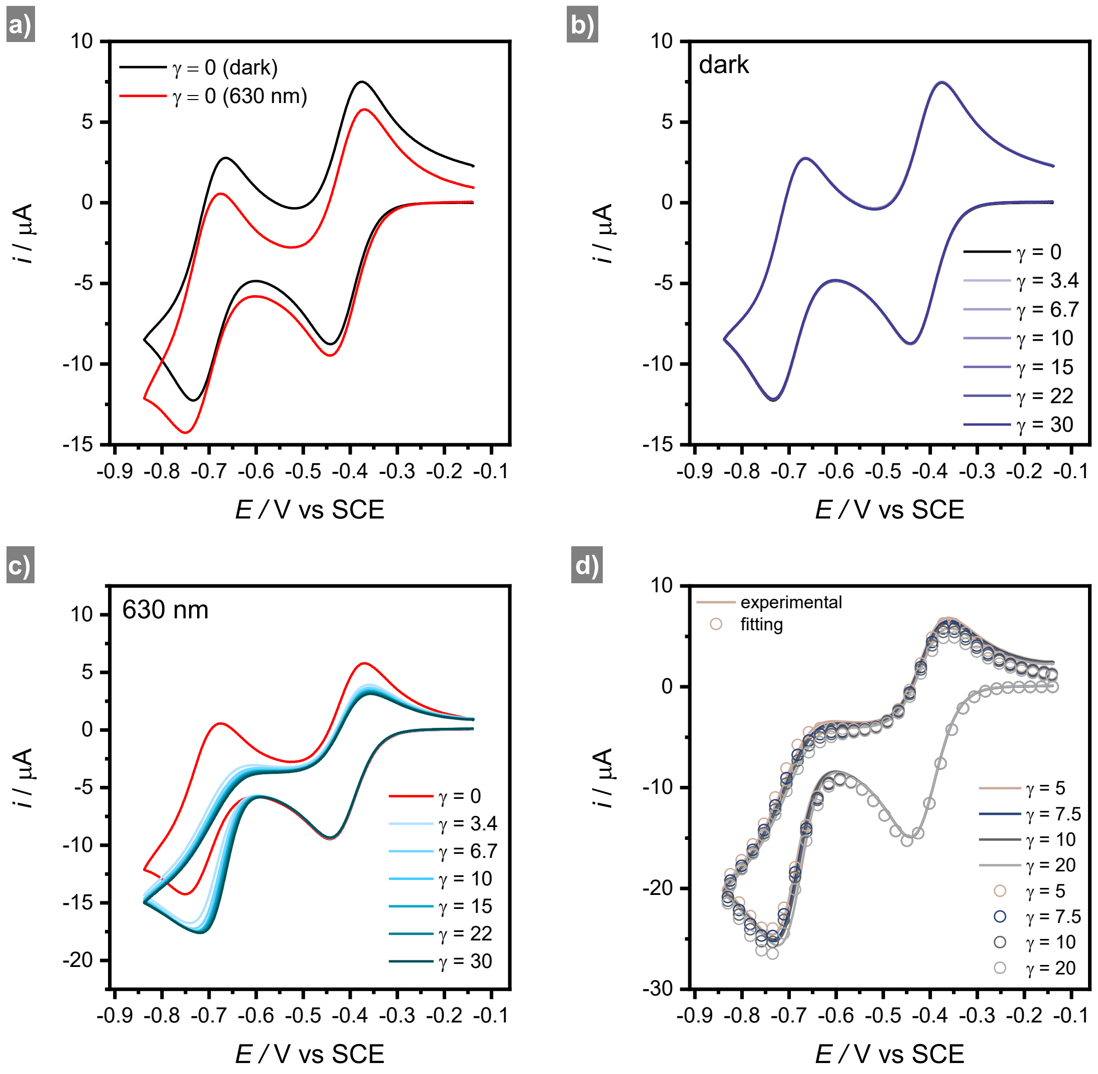

Figure S17. a) CVs of 10^{-3} M PDI in the absence of and under 630 nm light irradiation. $\upsilon = 0.06$ V s^{-1}. b) CVs of 10^{-3} M PDI in the absence and in the presence of PhI at different concentrations, labeled on the curves ($\gamma = C_{PhI}/C_{PDI}$). $\upsilon = 0.06$ V s^{-1}. c) Experimental CVs recorded at increasing concentration of PhI. $\upsilon = 0.06$ V s^{-1}. d) Comparison between experimental (lines) and simulated CVs (circles) at different PhI concentrations at $\upsilon = 0.15$ V s^{-1}. All the experimental CVs were recorded in DMF + 0.1 M *n*-Bu_4NBF_4 on a GC electrode ($A = 7.07$ mm^2) at $T = 25$ °C.

8.8 2-Bromopyridine (2-BrPy)

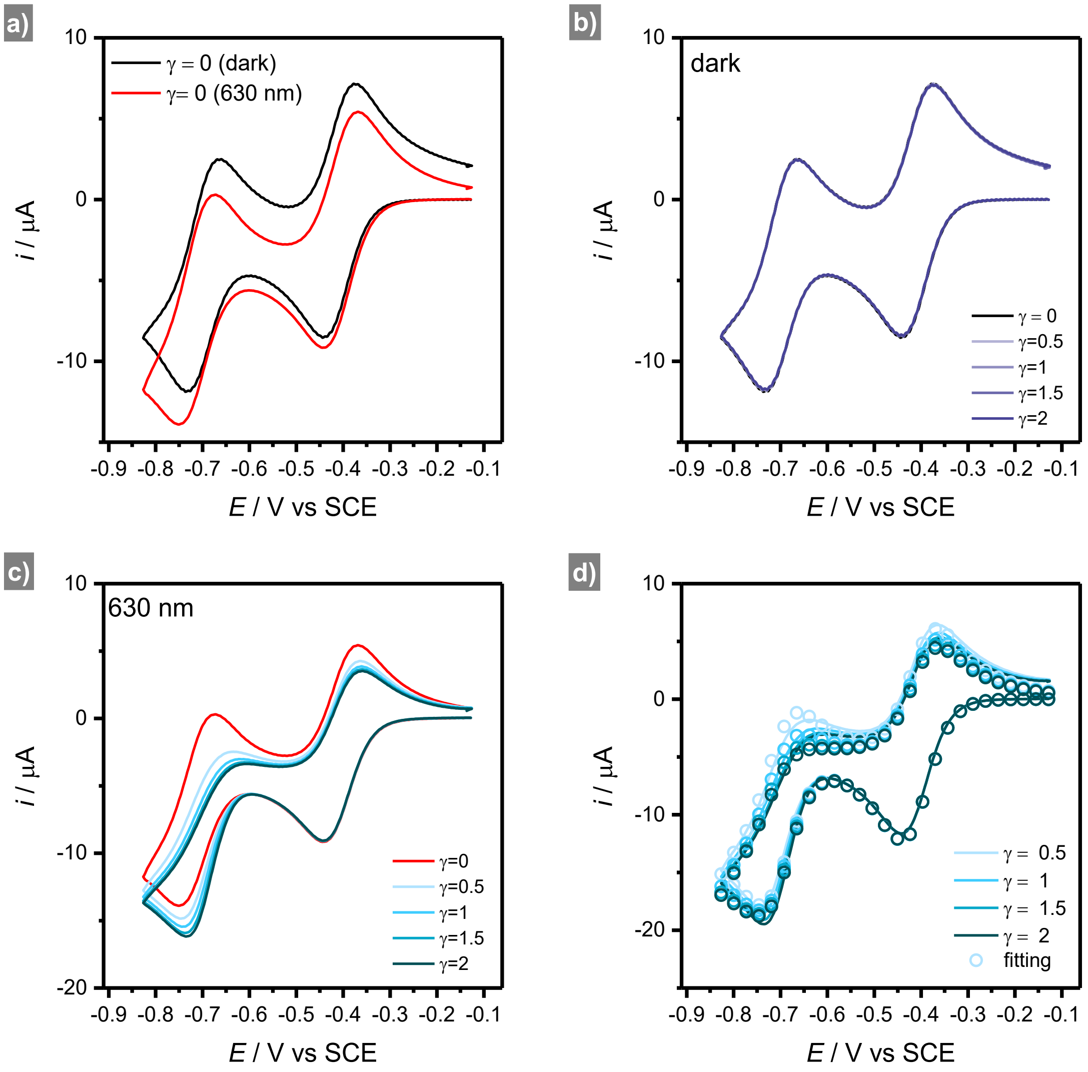

Figure S18. a) CVs of 10^{-3} M PDI in the absence of and under 630 nm light irradiation. $\upsilon = 0.06$ V s^{-1}. b) CVs of 10^{-3} M PDI in the absence and in the presence of 2-BrPy at different concentrations, labeled on the curves ($\gamma = C_{2\text{-BrPy}}/C_{\text{PDI}}$). $\upsilon = 0.06$ V s^{-1}. c) Experimental CVs recorded at increasing concentration of 2-BrPy. $\upsilon = 0.2$ V s^{-1}. d) Comparison between experimental (lines) and simulated CVs (circles) at different 2-BrPy concentrations at $\upsilon = 0.1$ V s^{-1}. All the experimental CVs were recorded in DMF + 0.1 M n-Bu_4NBF_4 on a GC electrode ($A = 7.07$ mm^2) at $T = 25$ °C.

8.9 2-Chloropyridine (2-ClPy)

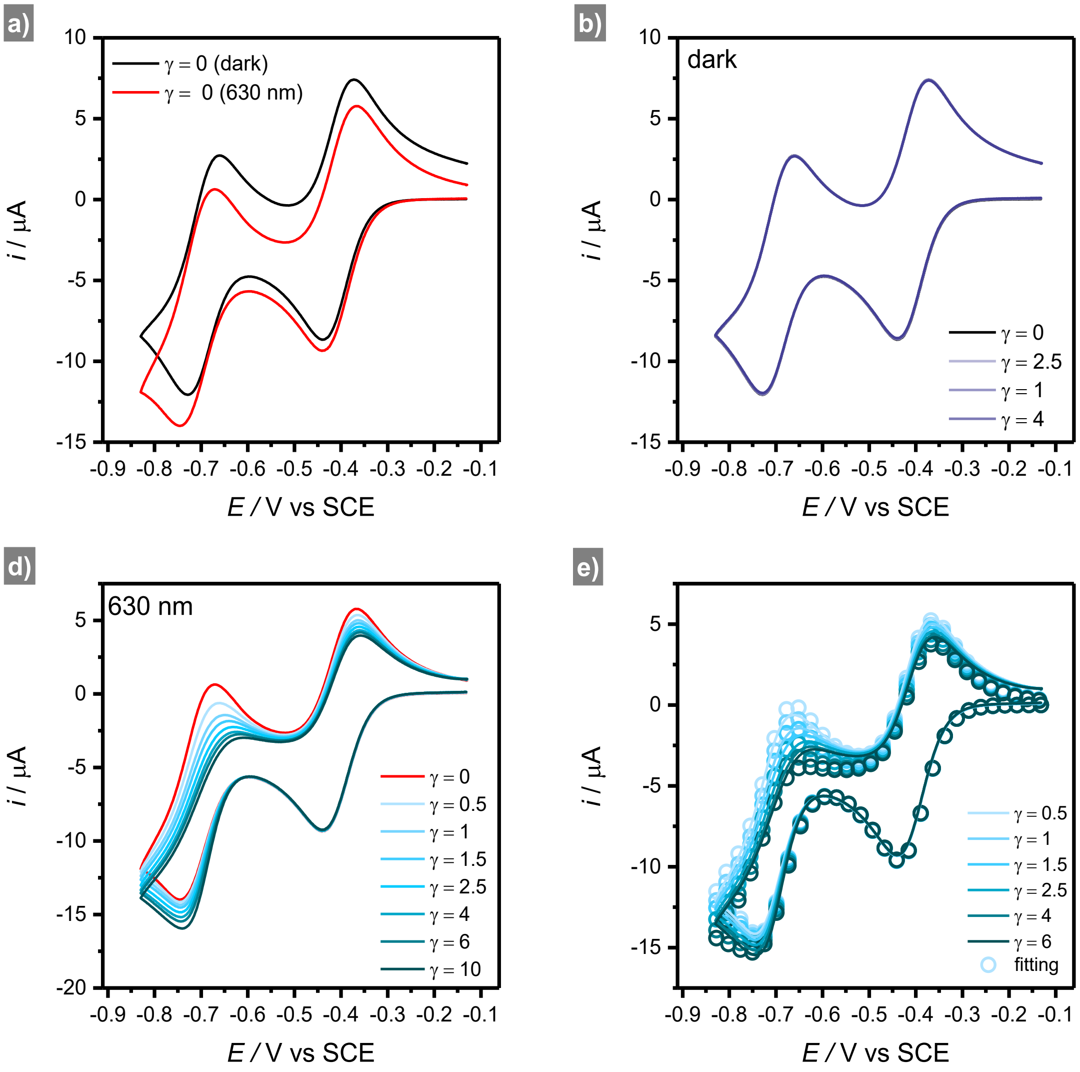

Figure S19. a) CVs of 10^{-3} M PDI in the absence of and under 630 nm light irradiation. $\upsilon = 0.06$ V s^{-1}. b) CVs of 10^{-3} M PDI in the absence and in the presence of 2-ClPy at different concentrations, labeled on the curves ($\gamma = C_{2\text{-ClPy}}/C_{\text{PDI}}$). $\upsilon = 0.06$ V s^{-1}. c) Experimental CVs recorded at increasing concentration of 2-ClPy. $\upsilon = 0.061$ V s^{-1}. d) Comparison between experimental (lines) and simulated CVs (circles) at different 2-ClPy concentrations at $\upsilon = 0.06$ V s^{-1}). All the experimental CVs were recorded in DMF + 0.1 M *n*-Bu$_4$NBF$_4$ on a GC electrode ($A = 7.07$ mm^2) at $T = 25$ °C.

8.10 Bromobenzene (PhBr)

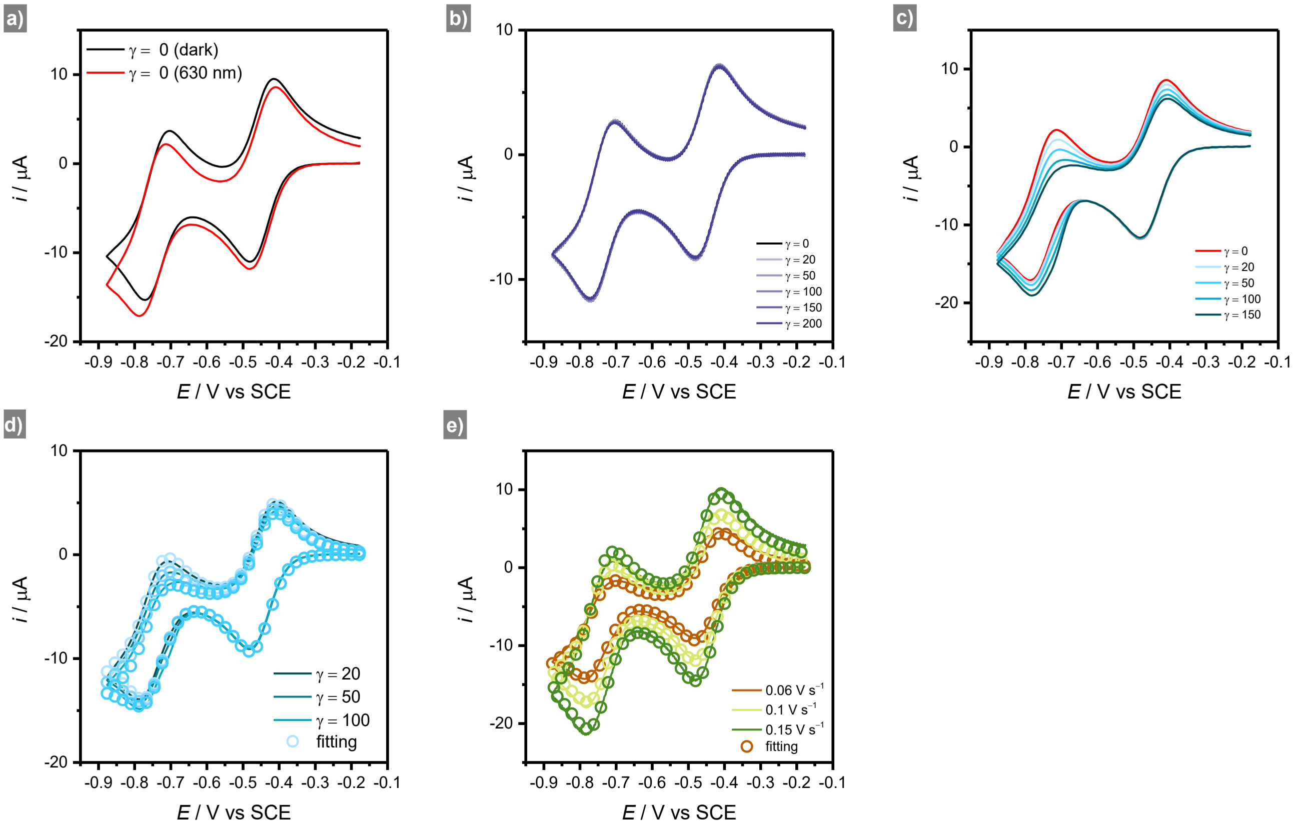

Figure S20. a) CVs of 10^{-3} M PDI in the absence of and under 630 nm light irradiation. $\upsilon = 0.1$ V s^{-1}. b) CVs of 10^{-3} M PDI in the absence and in the presence of PhBr at different concentrations, labeled on the curves ($\gamma = C_{PhBr}/C_{PDI}$). $\upsilon = 0.1$ V s^{-1}. c) Experimental CVs recorded at increasing concentration of PhBr. $\upsilon = 0.1$ V s^{-1}. d) Comparison between experimental (lines) and simulated CVs (circles) at different PhBr concentrations at $\upsilon = 0.06$ V s^{-1}. e) Comparison between experimental (lines) and simulated CVs (circles) at $5 \cdot 10^{-2}$ M PhBr concentrations and different scan rates. All the experimental CVs were recorded in DMF + 0.1 M n-Bu_4NBF_4 on a GC electrode ($A = 7.07$ mm^2) at $T = 25$ °C.

8.11 Chlorobenzene (PhCl)

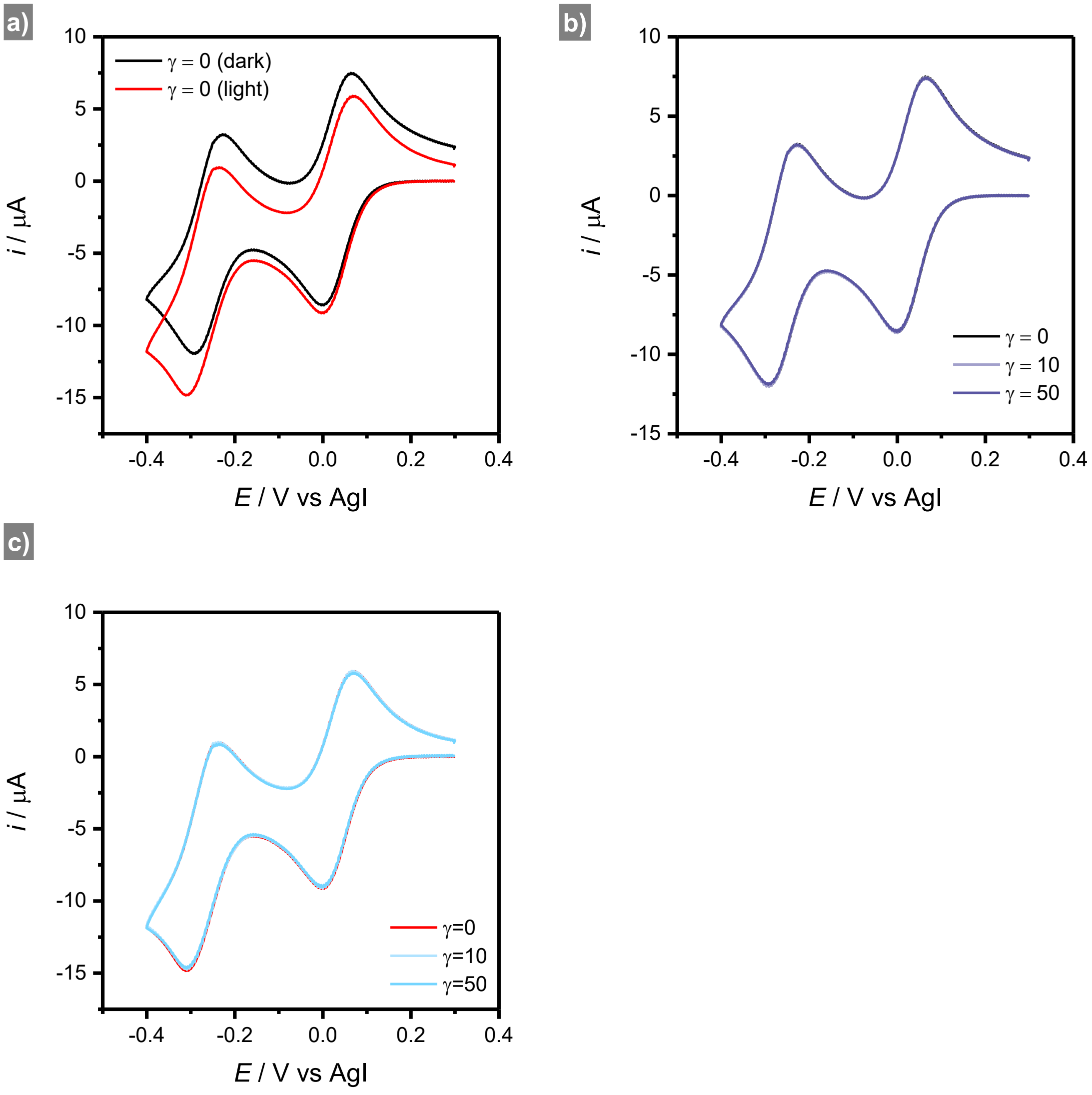

Figure S21. a) CVs of 10^{-3} M PDI in the absence of and under 630 nm light irradiation. $\upsilon = 0.06$ V s^{-1}. b) CVs of 10^{-3} M PDI in the absence and in the presence of PhCl at different concentrations, labeled on the curves ($\gamma = C_{PhCl}/C_{PDI}$). $v = 0.06$ V s^{-1}. c) Experimental CVs recorded at increasing concentration of PhCl. $\upsilon = 0.1$ V s^{-1}. All the experimental CVs were recorded in DMF + 0.1 M n-Bu$_4$NBF$_4$ on a GC electrode (A = 7.07 mm^2) at $T = 25$ °C

9 Experimental TCSPC data with relative fitting

From the exponential fitting of the luminescence decay of PDI^{2-} in the presence of various RXs (Figure S22), we obtained Stern–Volmer constants, K_{SV}, and consequently quenching rate constants as reported in Table S8.

Table S8. Values of K_{SV} and k_q obtained from the evaluation of experimentally registered TCSPC data using the Stern–Volmer equation.

Substrate	K_{SV} M^{-1}	k_q M^{-1} s^{-1}
1-BrNaph	30.7	$5.0 \cdot 10^9$
PhI	10.7	$1.6 \cdot 10^9$
PhBr	0.072	$1.3 \cdot 10^7$

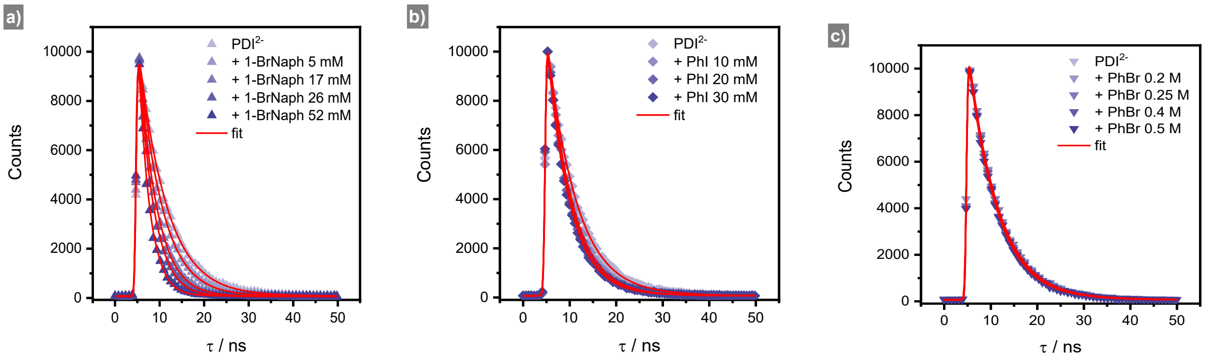

Figure S22. Luminescence decays of PDI^{2-} alone and in the presence of 1-BrNaph (a), PhI (b), PhBr (c), following excitation at 633.6 nm, and detection at 657 nm. Different concentrations of RX were added as indicated in the legends. The experiments were performed at room temperature. PDI^{2-} was electrochemically generated by reducing a solution of PDI $3\cdot10^{-4}$ M + 0.1 M n-Bu_4NBF_4 at a potential of −0.83 V vs SCE on a reticulated vitreous carbon electrode.

10 Fitting evaluation excluding the $PDI^{•−}$ + $R^{•}$ coupling reaction

To validate the proposed mechanism and specifically assess the significance of the coupling reaction between $PDI^{•−}$ and $R^{•}$, a comparative kinetic analysis was performed. We fitted the experimental data using both the complete kinetic model and a modified version in which the $PDI^{•−}/R^{•}$ coupling step was omitted. A direct comparison of the fit goodness for a series of substrates (Figure S23-Figure S25) corroborates that this reaction is crucial for accurately reproducing the experimental kinetic traces.

4-Methylchlorobenzoate (4-MCB)

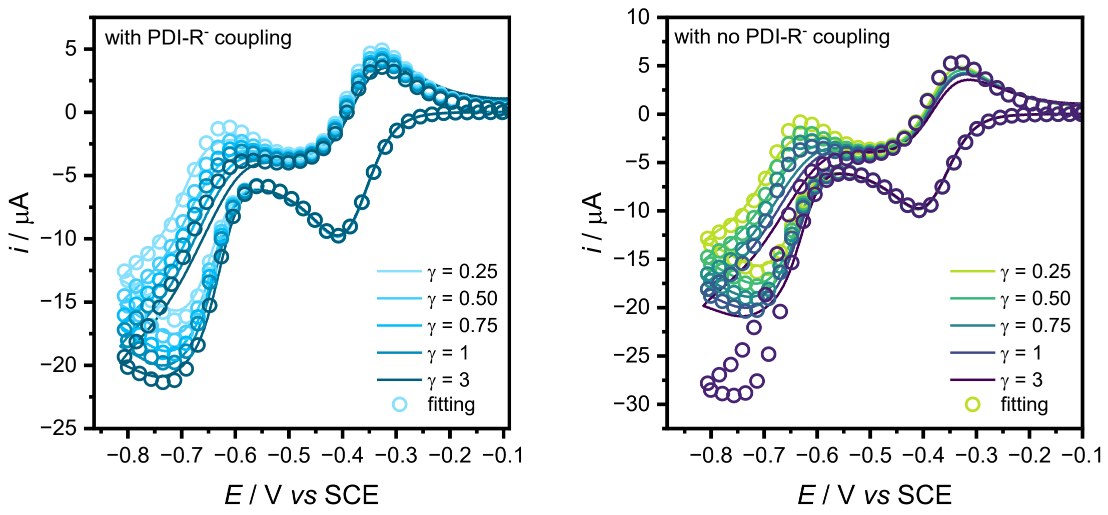

Figure S23. Comparison of experimental (solid lines) and simulated (circles) CVs of 10^{-3} PDI + 4-MCB (varying amounts, labelled on the curves as $\gamma = C_{4\text{-MCB}}/C_{\text{PDI}}$) under 630 nm light irradiation. Simulations were performed using a kinetic model both **with** (left) and **without** (right) the $PDI^{•−}/R^{•}$ coupling step (as labeled on the figures). CVs were recorded on a GC electrode (A = 7.07 mm^2). All solutions were prepared in DMF + 0.1 M n-Bu_4NBF_4 and thermostated at 25 °C.

1-Bromonaphthalene (1-BrNaph)

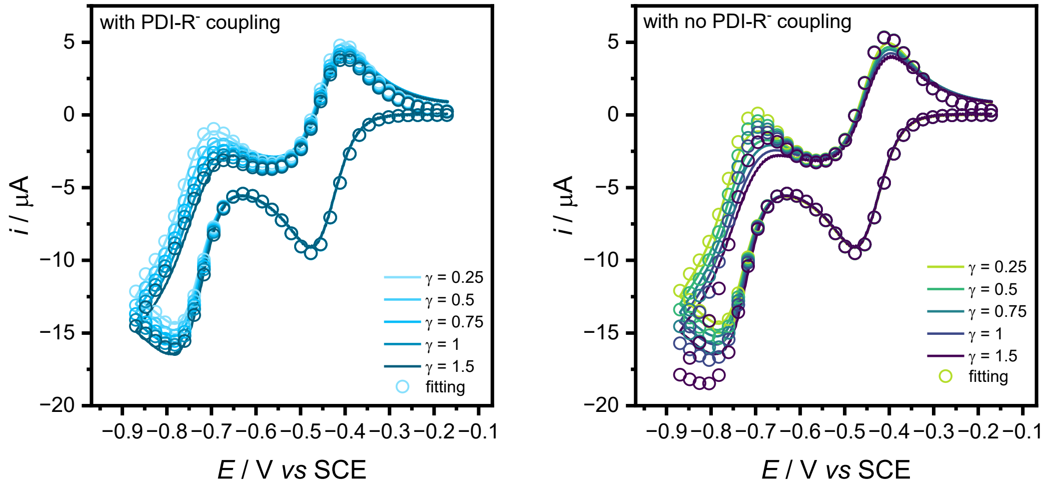

Figure S24. Comparison of experimental (solid lines) and simulated (circles) CVs of 10^{-3} PDI + 1-BrNaph (varying amounts, labelled on the curves as $\gamma = C_{\text{1-BrNaph}}/C_{\text{PDI}}$) under 630 nm light irradiation. Simulations were performed using a kinetic model both **with** (left) and **without** (right) the $PDI^{\bullet -}/R^{\bullet}$ coupling step (as labeled on the figures). CVs were recorded on a GC electrode (A = 7.07 mm^2). All solutions were prepared in DMF + 0.1 M n-Bu_4NBF_4 and thermostated at 25 °C.

2-Chloropyridine (2-ClPy)

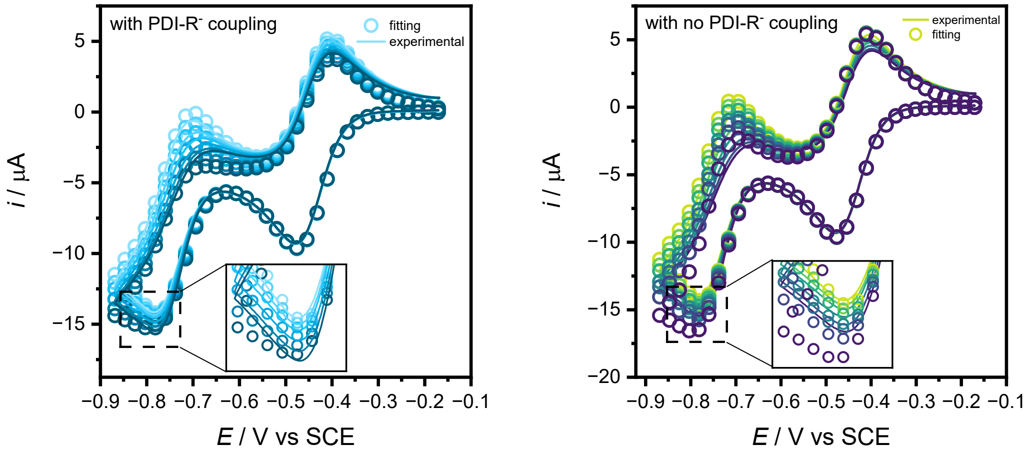

Figure S25. Comparison of experimental (solid lines) and simulated (circles) CVs of 10^{-3} PDI + 2-ClPy (varying amounts, labelled on the curves as $\gamma = C_{\text{2-ClPy}}/C_{\text{PDI}}$) under 630 nm light irradiation. Simulations were performed using a kinetic model both **with** (left) and **without** (right) the $PDI^{\bullet -}/R^{\bullet}$ coupling step (as labeled on the figures). CVs were recorded on a GC electrode (A = 7.07 mm^2). All solutions were prepared in DMF + 0.1 M n-Bu_4NBF_4 and thermostated at 25 °C.

11 Spectroelectrochemistry in the presence of RX

UV-Vis spectra of electrogenerated $PDI^{•-}$ and PDI^{2-} in the absence of in the presence of a 100× excess of alkyl halide methyl 4-chlorobenzoate (4-MCB), indicating that there is likely no precomplexation between the reduced PDI species and this substrate.

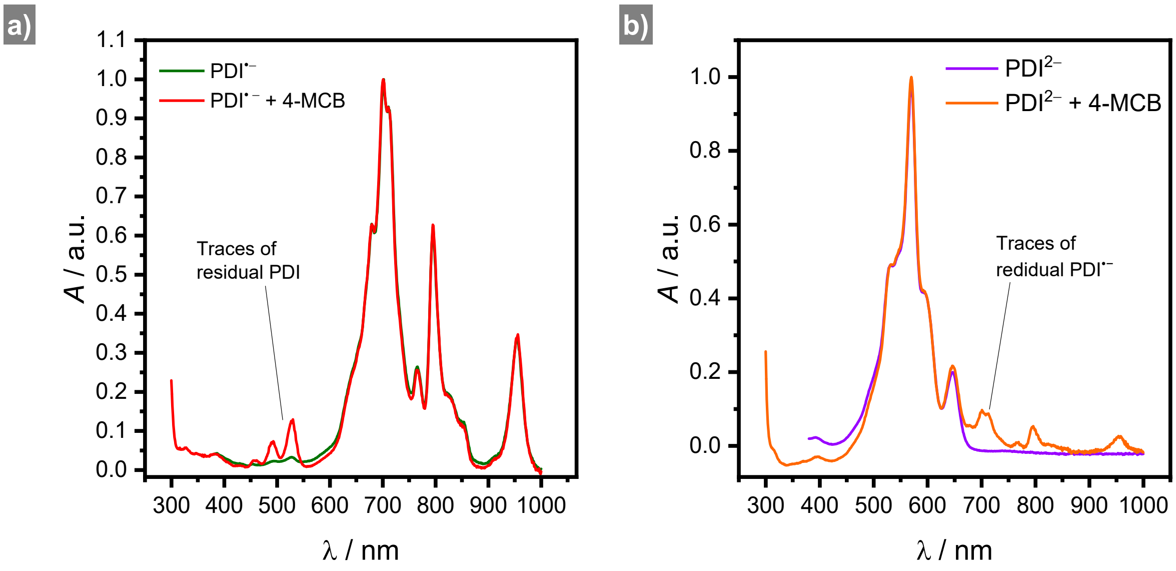

Figure S26. Vis-NIR absorption spectra of (a) $PDI^{•-}$ (green line) and $PDI^{•-}$ + 4-MCB (red line) in DMF + 0.1 M n-Bu_4NBF_4. (b) PDI^{2-} (purple line) and PDI^{2-} + 4-MCB (orange line) in DMF + 0.1 M n-Bu_4NBF_4. $PDI^{•-}$ and PDI^{2-} were generated in a degassed spectroelectrochemical cell (path length l = 0.5 mm) by reduction of $2.5 \cdot 10^{-4}$ M PDI on a Pt mesh electrode at −0.55 V vs SCE and −1.2 V vs SCE, respectively, in the absence and presence of $2.5 \cdot 10^{-2}$ M 4-MCB.

12 CVs under different wavelengths

CVs were recorded under electrode irradiation. The emission spectra of the lamps are shown below, overlapped with the absorption spectra of PDI and its reduced species. The reactivity of $^*PDI^{2-}$ was high at 630 nm and almost negligible at 730 nm, where PDI^{2-} does not absorb. The reactivity of $^*PDI^{\bullet-}$ also followed the expected trend: more catalytic current was observed with the 630 nm and 730 nm lamps, where light absorption is high, while almost no reactivity was detected at 595 nm, where absorption is low.

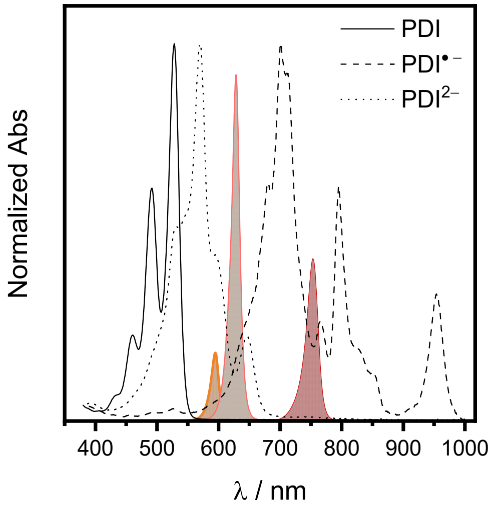

Figure S27. Emission spectra of the utilized lamps (coloured areas) and absorption spectra of PDI and its reduced species (black lines) as labelled in the legend.

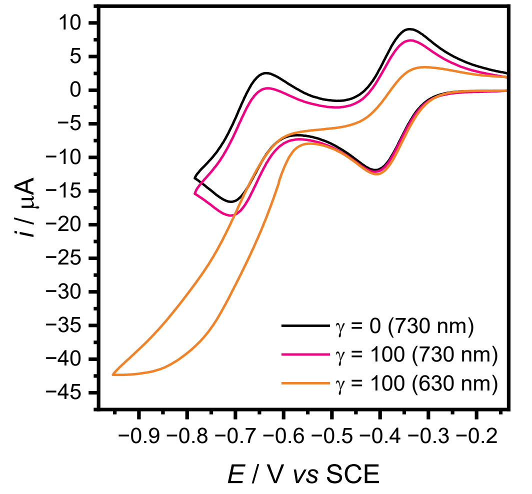

Figure S28. CVs of 10^{-3} M PDI recorded in the absence and in the presence of MBiB, labeled on the curves ($\gamma = C_{MBiB}/C_{PDI}$), both in dark and under 630 nm or 730 nm irradiation. CVs recorded in DMF + 0.1 M n-Bu_4NBF_4 at $\upsilon = 0.1$ V s^{-1} and thermostated at 25 °C.

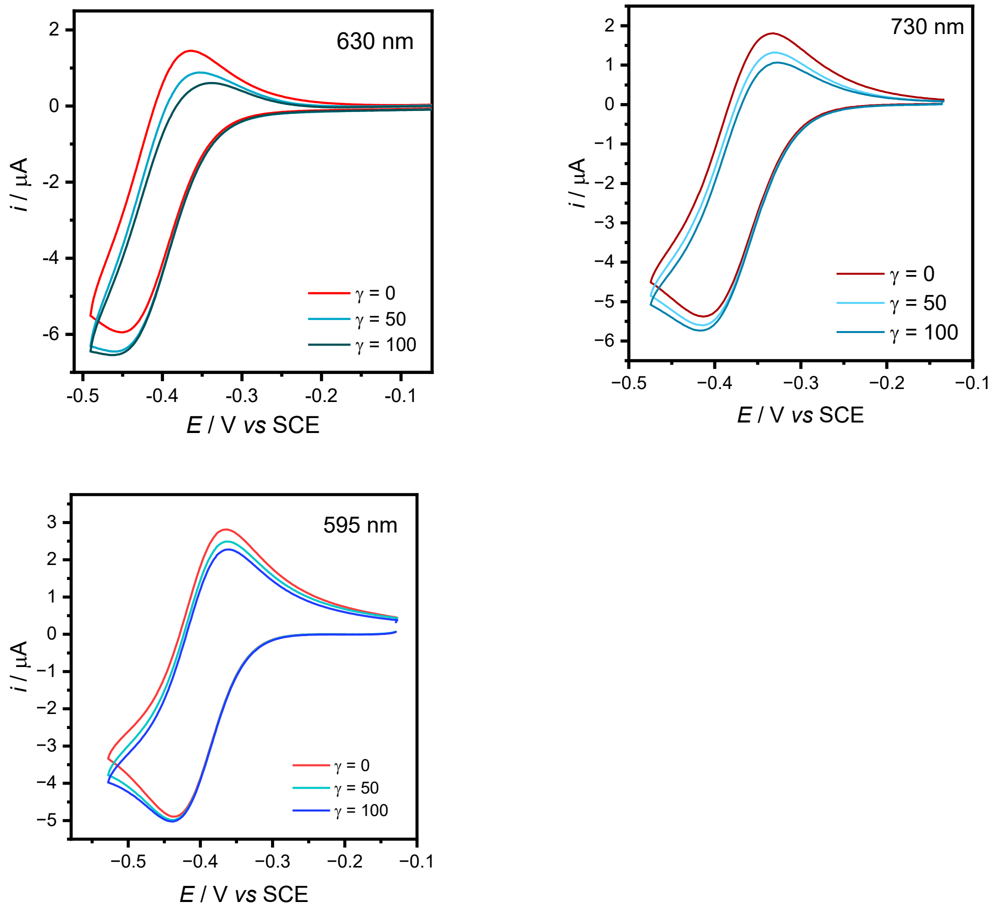

Figure S29. CVs of 10^{-3} M PDI recorded (a) under different lamp irradiation (labelled on the respective graphs) in the presence of varying amounts of MBiB, labelled on the curves ($\gamma = C_{MBiB}/C_{PDI}$) under irradiation. All CVs are recorded in DMF + 0.1 M n-Bu_4NBF_4 at $\upsilon = 0.02$ V s^{-1} and thermostated at 25 °C. Similar catalytic currents were observed under 630 and 730 nm irradiation, where the absorbance of $PDI^{\bullet-}$ is high. In contrast, only a much smaller catalytic current was detected at 595 nm, despite its higher photon energy, as the absorbance of $PDI^{\bullet-}$ is low at this wavelength.

13 Constant potential electrolysis

The behaviour of PDI-based electrophotocatalysts in the presence of RX species was tested in bulk photoelectrolysis for a series of substrates. Overall, these results confirmed that

i) *PDI$^{•−}$ was a capable catalyst for the reductive cleavage of MBiB
ii) The catalysts were fairly stable while operating in the presence of an alkyl halide (MBiB) as susbtrate, but were not when operating in the presence of aryl halides.

Table S9. Bulk photoelectrolysis experiments.

Entry	Catalyst	RX	Ratio	Charge passed (compared to RX)
1	*PDI$^{•−}$	MBiB	1:10	1.42 F/mol
2	*PDI$^{•−}$	4-Iodobenzaldehyde	1:20	0.20 F/mol
3	*PDI$^{2−}$	Methyl 4-chlrobenzoate	1:20	0.15 F/mol

13.1 CPE of MBiB

First PDI alone was reduced via constant potential electrolysis (CPE) at −0.48 V vs SCE to form PDI$^{•−}$ on a bulk Pt gauze electrode. The current decayed to < 4% of its initial value after 3 h (Figure S30a, below) and the total consumed charge was 1.2 F/mol catalyst, overall in agreement with the monoelectronic reduction of PDI to its radical anion. This was also confirmed by a color change from bright fluorescent orange (typical color of a PDI solution in DMF) to dark green (the observed color for the radical anion in DMF).

In a second experiment, electrolysis of PDI at −0.48 V vs SCE was performed in the presence of a 10-fold excess of methyl α-bromoisobutyrrate (MBiB). The electrolysis was started in the dark and the measured current decay matched that observed in the absence of substrate (Figure S30a), indicating the absence of any catalytic reaction between PDI$^{•−}$ and MBiB. After 1 h electrolysis in the dark, the 630 nm light was switched on and the chronoamperometry profile drastically changed (blue line in Figure S30a). The current did not continue to decrease, but instead, increased in absolute terms until it returned to its initial value after 4 h, indicating the presence of an electrophotocatalytic cycle.

After about 6 h, 4.5 F/mol of charge (vs catalyst) was passed and the solution color gradually transitioned from green to green-orange, typical of PDI. After 24 h, the charge consumed was 14.2 F/mol vs catalyst, or 1.42 F/mol vs the alkyl halide.

CV was recorded before and during CPE across the full scan range, including direct MBiB reduction. Before photoelectrolysis, reversible catalyst signals and MBiB's irreversible reduction were observed. During electrolysis, the MBiB signal diminished and nearly disappeared after overnight irradiation, while an irreversible oxidation at +0.7 V vs SCE appeared, attributed to Br^- oxidation from R–Br cleavage. Catalyst signals also decreased, likely from side reactions with radicals and $PDI^{\bullet-}$. UV–Vis data corroborated the CVs, showing catalyst stability for ~6 h, followed by partial decomposition after overnight irradiation (Figure S31b,c). Overall, the catalyst remains active long enough to fully consume MBiB.

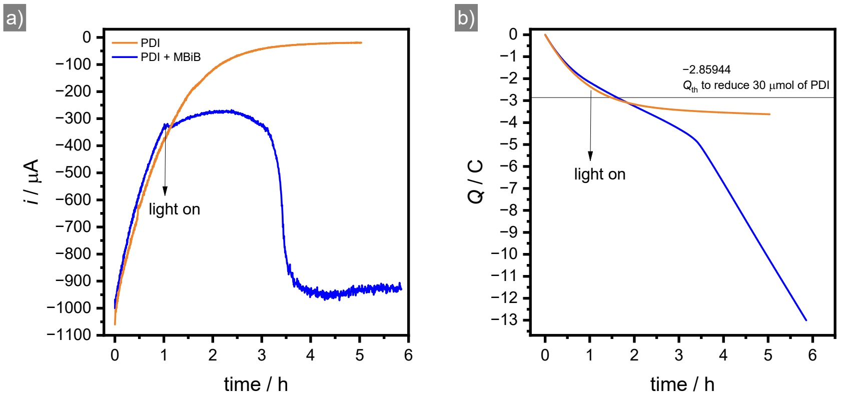

Figure S30. Chronoamperometry (a) and relative passed charge (b) for the electrolysis of PDI alone (orange line) and in the presence of MBiB (blue line). Electrolysis experiments were performed on a solution of $2 \cdot 10^{-3}$ M PDI (orange line) and $2 \cdot 10^{-3}$ M PDI + $2 \cdot 10^{-2}$ M MBiB in DMF + 0.1 M n-Bu_4NBF_4, using a Pt mesh working electrode; $T = 25$ °C; applied potential $E_{app} = -0.48$ V vs SCE. 630 nm light irradiation.

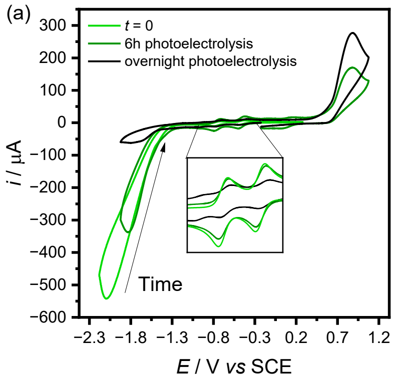

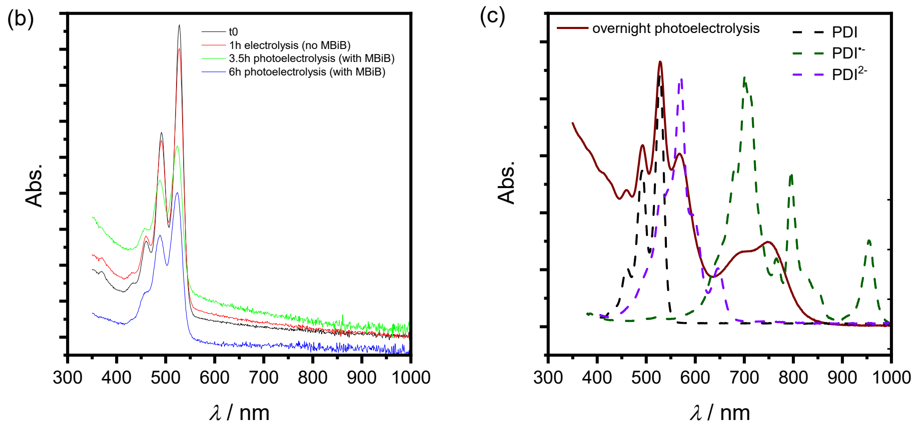

Figure S31. (a) CVs of $2 \cdot 10^{-3}$ M PDI recorded in the presence of 0.02 M MBiB after different photo-electrolysis times. CVs recorded in DMF + 0.1 M n-Bu_4NBF_4 at $\upsilon = 0.1$ V s^{-1} and thermostated at 25 °C. (b) Quantitative UV-Vis-NIR spectra of a 1:100 diluted reaction mixture (so to have a $2 \cdot 10^{-5}$ M theorical concentration of PDI) of: solution of PDI alone before any sort of reaction (black); solution of PDI alone after 1 h electrolysis (red), solution of PDI + MBiB after 3.5h (green) and 6h (blue) photoelectrolysis under 630 nm. (c) UV-Vis-NIR normalized spectra of PDI, $PDI^{\bullet-}$ and PDI^{2-} (dashed lines) and of the reaction crude after 24 h of photoelectrolysis of PDI + MBiB under 630 nm irradiation.

13.2 CPE of 4-IBA

Additionally, we performed a CPE experiment under 630 nm irradiation in the presence of 4-iodobenzaldehyde ($E^0 \approx -1.77$ V *vs* SCE) applying −0.50 V vs SCE to form $PDI^{\bullet-}$ on a bulk Pt mesh electrode. After 6 h, the current went to nearly zero, and only 0.2 F/mol vs aryl halide were passed. Cyclic voltammetry recorded before and after CPE over the $PDI/PDI^{\bullet-}$ redox window showed that the characteristic reversible PDI couple was clearly observed prior to photoelectrolysis, but already after 3 h the signals had almost completely disappeared (Figure S32a). UV-Vis confirmed decomposition fo the catalyst. Conversion of 4-IBA via CV and NMR was small, ca. 5%.

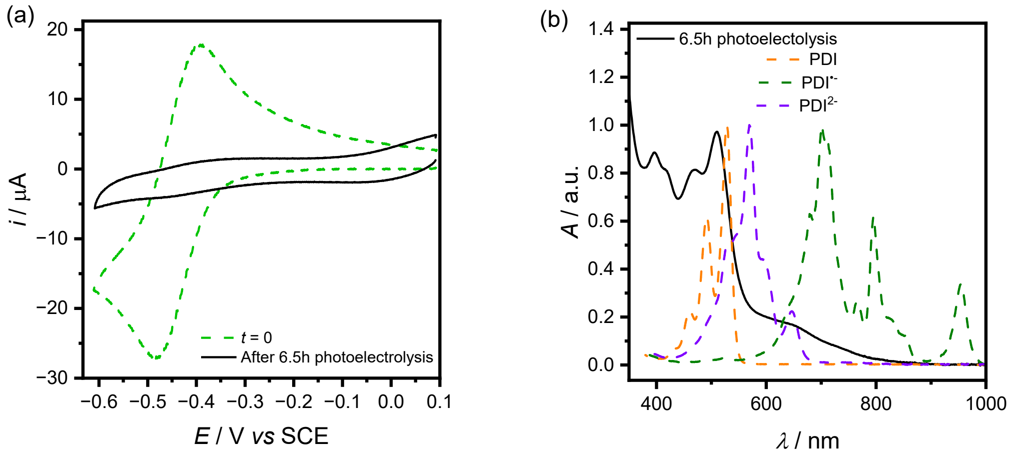

Figure S32. The electrolysis experiment was performed on a solution of $1.5 \cdot 10^{-3}$ M PDI + $3 \cdot 10^{-2}$ M 4-IBA in DMF + 0.1 M n-Bu_4NBF_4, using a Pt mesh working electrode, under 630 nm light irradiation; T = 25 °C; applied potential E_{app} = −0.501 V vs SCE. (a) CVs of $1.5 \cdot 10^{-3}$ M PDI recorded in the presence of $3 \cdot 10^{-2}$ M 4-IBA before (dashed line) and after (solid line) 6.5 h of photo-electrolysis under 630 nm irradiation. CVs recorded on a GC electrode (A = 7.07 mm^2) in DMF + 0.1 M n-Bu_4NBF_4 at υ = 0.2 V s^{-1} and thermostated at 25 °C. (b) UV-Vis-NIR normalized spectra of the reaction mixture after 6.5h photo-electrolysis (solid black line) and spectra of PDI and its reduced species (dashed lines) for reference.

13.3 CPE of 1-BrNaph

To probe PDI^{2-} under bulk photoelectrolysis, CPE was performed with 1-bromonaphthalene (E^0 = −2.17 V vs SCE) at −0.791 V vs SCE on a Pt mesh. After ~3 h of 630 nm irradiation, the cathodic current fell to ≈ 3% of its initial value, corresponding to an onverall 0.15 F/mol charge. CV before and after CPE, Figure

S33a), showed that the two reversible PDI waves present initially had nearly disappeared after 3 h, replaced by new redox features. Figure S33b confirms catalyst decomposition after electrolysis.

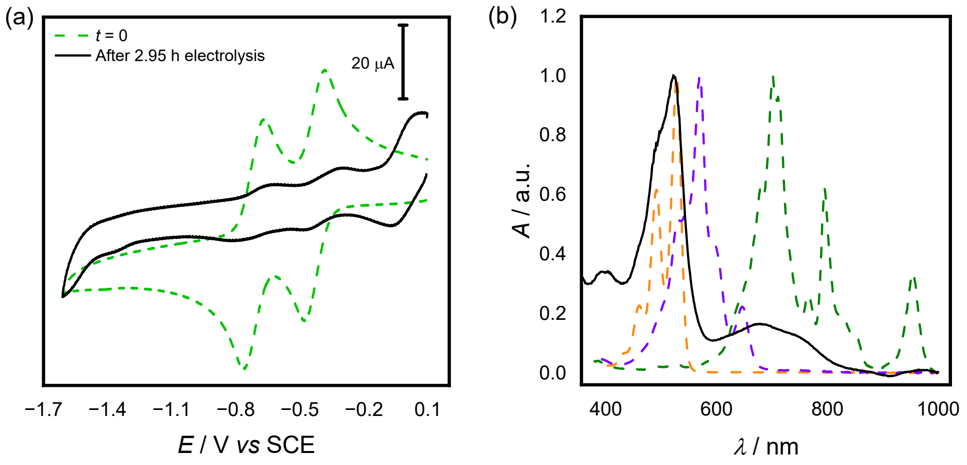

Figure S33. The electrolysis experiment was performed on a solution of $2 \cdot 10^{-3}$ M PDI + $4 \cdot 10^{-2}$ M 1-BrNaph in DMF + 0.1 M n-Bu_4NBF_4, using a Pt mesh working electrode, under 630 nm light irradiation; $T = 25$ °C; applied potential $E_{app} = -0.791$ V vs SCE. (a) CVs of $2 \cdot 10^{-3}$ M PDI recorded in the presence of $4 \cdot 10^{-2}$ M 1-BrNaph before (dashed line) and after (solid line) 2.95h of photo-electrolysis under 630 nm irradiation. CVs recorded on a GC electrode (A = 7.07 mm^2) in DMF + 0.1 M n-Bu_4NBF_4 at $\upsilon = 0.2$ V s^{-1} and thermostated at 25 °C. (b) UV-Vis-NIR normalized spectra of the reaction mixture after 2.95h photo-electrolysis (solid black line) and spectra of PDI and its reduced species (dashed lines) for reference.

14 Estimation of energy loss for photocatalysis with $^*PDI^{\bullet-}$

The energy loss for the use of $PDI^{\bullet-}$ irradiated by 630 nm light was estimated. The energy of 630 nm light is 2.0 eV. However, the effective "band gap" energy of $PDI^{\bullet-}$ $E_{0\text{-}0}$ can be calculated from $E^0_{PDI/{}^*PDI^{\bullet-}} = E^0_{PDI/PDI^{\bullet-}} - E_{0\text{-}0}$, with $E^0_{PDI/{}^*PDI^{\bullet-}} = -1.87$ V vs SCE and $E^0_{PDI/PDI^{\bullet-}} = -0.41$ V vs SCE, resulting in $E_{0\text{-}0} = 1.5$ eV. Therefore, roughly 0.5 eV is lost in the internal conversion processes of $^*PDI^{\bullet-}$. The energy loss will be even higher if the radical anion is irradiated for example with yellow or blue light.

References

(1) Frisch, M.; Trucks, G.; Schlegel, H.; Scuseria, G.; Robb, M.; Cheeseman, J.; Scalmani, G.; Barone, V.; Petersson, G.; Nakatsuji, H. Gaussian 16 Revision C. 01, 2016. *Gaussian Inc. Wallingford CT* **2016**, *1*, 572.
(2) Lee, C.; Yang, W.; Parr, R. G. Development of the Colle-Salvetti correlation-energy formula into a functional of the electron density. *Physical review B* **1988**, *37*, 785.
(3) Becke, A. D. A new mixing of Hartree-Fock and local density-functional theories. *Journal of chemical Physics* **1993**, *98*, 1372.
(4) Marenich, A. V.; Cramer, C. J.; Truhlar, D. G. Universal solvation model based on solute electron density and on a continuum model of the solvent defined by the bulk dielectric constant and atomic surface tensions. *The Journal of Physical Chemistry B* **2009**, *113*, 6378.
(5) Martin, R. L. Natural transition orbitals. *Journal of Chemical Physics* **2003**, *118*, 4775.
(6) Pham, L. N.; Olding, A.; Ho, C. C.; Bissember, A. C.; Coote, M. L. Investigating Competing Inner-and Outer-Sphere Electron-Transfer Pathways in Copper Photoredox-Catalyzed Atom-Transfer Radical Additions: Closing the Cycle. *Angewandte Chemie International Edition* **2025**, *64*, e202415792.
(7) Nelsen, S. F.; Blackstock, S. C.; Kim, Y. Estimation of inner shell Marcus terms for amino nitrogen compounds by molecular orbital calculations. *Journal of the American Chemical Society* **1987**, *109*, 677.
(8) López-Estrada, O.; Laguna, H. G.; Barrueta-Flores, C.; Amador-Bedolla, C. Reassessment of the four-point approach to the electron-transfer Marcus–Hush theory. *ACS omega* **2018**, *3*, 2130.
(9) Durante, C.; Isse, A. A.; Todesco, F.; Gennaro, A. Electrocatalytic activation of aromatic carbon-bromine bonds toward carboxylation at silver and copper cathodes. *Journal of The Electrochemical Society* **2013**, *160*, G3073.
(10) Falciola, L.; Gennaro, A.; Isse, A. A.; Mussini, P. R.; Rossi, M. The solvent effect in the electrocatalytic reduction of organic bromides on silver. *Journal of Electroanalytical Chemistry* **2006**, *593*, 47.
(11) Nicholson, R. S. Theory and Application of Cyclic Voltammetry for Measurement of Electrode Reaction Kinetics. *Analytical Chemistry* **1965**, *37*, 1351.
(12) Marcus, R. A.; Sutin, N. Electron transfers in chemistry and biology. *Biochimica et Biophysica Acta (BBA)-Reviews on Bioenergetics* **1985**, *811*, 265.
(13) Saveant, J. M. A simple model for the kinetics of dissociative electron transfer in polar solvents. Application to the homogeneous and heterogeneous reduction of alkyl halides. *Journal of the American Chemical Society* **1987**, *109*, 6788.
(14) Cardinale, A.; Isse, A. A.; Gennaro, A.; Robert, M.; Savéant, J.-M. Dissociative electron transfer to haloacetonitriles. An example of the dependency of in-cage ion-radical interactions upon the leaving group. *Journal of the American Chemical Society* **2002**, *124*, 13533.
(15) Pause, L.; Robert, M.; Saveant, J.-M. Reductive cleavage of carbon tetrachloride in a polar solvent. An example of a dissociative electron transfer with significant attractive interaction between the caged product fragments. *Journal of the American Chemical Society* **2000**, *122*, 9829.
(16) Pause, L.; Robert, M.; Saveant, J.-M. Stepwise and concerted pathways in photoinduced and thermal electron-transfer/bond-breaking reactions. Experimental illustration of similarities and contrasts. *Journal of the American Chemical Society* **2001**, *123*, 4886.
(17) Saveant, J. M. Dissociative electron transfer. New tests of the theory in the electrochemical and homogeneous reduction of alkyl halides. *Journal of the American Chemical Society* **1992**, *114*, 10595.
(18) Isse, A. A.; Gennaro, A. Homogeneous reduction of haloacetonitriles by electrogenerated aromatic radical anions: determination of the reduction potential of• CH2CN. *The Journal of Physical Chemistry A* **2004**, *108*, 4180.
(19) Costentin, C.; Robert, M.; Savéant, J.-M. Electron transfer and bond breaking: Recent advances. *Chemical physics* **2006**, *324*, 40.
(20) Isse, A. A.; Gennaro, A.; Lin, C. Y.; Hodgson, J. L.; Coote, M. L.; Guliashvili, T. Mechanism of Carbon−Halogen Bond Reductive Cleavage in Activated Alkyl Halide Initiators Relevant to Living Radical Polymerization: Theoretical and Experimental Study. *Journal of the American Chemical Society* **2011**, *133*, 6254.
(21) Isse, A. A.; Lin, C. Y.; Coote, M. L.; Gennaro, A. Estimation of Standard Reduction Potentials of Halogen Atoms and Alkyl Halides. *The Journal of Physical Chemistry B* **2011**, *115*, 678.
(22) Enemærke, R. J.; Christensen, T. B.; Jensen, H.; Daasbjerg, K. Application of a new kinetic method in the investigation of cleavage reactions of haloaromatic radical anions. *Journal of the Chemical Society, Perkin Transactions 2* **2001**, 1620.
(23) Kojima, H.; Bard, A. J. Determination of rate constants for the electroreduction of aromatic compounds and their correlation with homogeneous electron transfer rates. *Journal of the American Chemical Society* **1975**, *97*, 6317.
(24) Donkers, R. L.; Maran, F.; Wayner, D. D. M.; Workentin, M. S. Kinetics of the Reduction of Dialkyl Peroxides. New Insights into the Dynamics of Dissociative Electron Transfer1. *Journal of the American Chemical Society* **1999**, *121*, 7239.
(25) Lin, C. Y.; Coote, M. L.; Gennaro, A.; Matyjaszewski, K. Ab Initio Evaluation of the Thermodynamic and Electrochemical Properties of Alkyl Halides and Radicals and Their Mechanistic Implications for Atom Transfer Radical Polymerization. *Journal of the American Chemical Society* **2008**, *130*, 12762.
(26) Gong, H.-X.; Cao, Z.; Li, M.-H.; Liao, S.-H.; Lin, M.-J. Photoexcited perylene diimide radical anions for the reduction of aryl halides: a bay-substituent effect. *Organic Chemistry Frontiers* **2018**, *5*, 2296.

(27) Pan, X.; Fang, C.; Fantin, M.; Malhotra, N.; So, W. Y.; Peteanu, L. A.; Isse, A. A.; Gennaro, A.; Liu, P.; Matyjaszewski, K. Mechanism of Photoinduced Metal-Free Atom Transfer Radical Polymerization: Experimental and Computational Studies. *Journal of the American Chemical Society* **2016**, *138*, 2411.
(28) Rehm, D.; Weller, A. Kinetics of Fluorescence Quenching by Electron and H-Atom Transfer. *Israel Journal of Chemistry* **1970**, *8*, 259.
(29) Eberson, L.; Eberson, L. Theories of electron transfer in organic chemistry. *Electron Transfer Reactions in Organic Chemistry* **1987**, 20.
(30) Agmon, N.; Levine, R. Energy, entropy and the reaction coordinate: thermodynamic-like relations in chemical kinetics. *Chemical Physics Letters* **1977**, *52*, 197.
(31) Zeman, C. J. I. V.; Kim, S.; Zhang, F.; Schanze, K. S. Direct Observation of the Reduction of Aryl Halides by a Photoexcited Perylene Diimide Radical Anion. *Journal of the American Chemical Society* **2020**, *142*, 2204.
(32) Brouwer, A. C.; Kirsch, J. F. Investigation of diffusion-limited rates of chymotrypsin reactions by viscosity variation. *Biochemistry* **1982**, *21*, 1302.
(33) Bortolamei, N.; Isse, A. A.; Gennaro, A. Estimation of standard reduction potentials of alkyl radicals involved in atom transfer radical polymerization. *Electrochimica Acta* **2010**, *55*, 8312.
(34) Marcus, R. A. On the Theory of Oxidation—Reduction Reactions Involving Electron Transfer. V. Comparison and Properties of Electrochemical and Chemical Rate Constants. *The Journal of Physical Chemistry* **1963**, *67*, 853.
(35) Larsen, H.; Pedersen, S. U.; Pedersen, J. A.; Lund, H. Self-exchange electron transfer rate constants and reorganization energies for some aromatic compounds in N,N-dimethylformamide determined by elect. *Journal of Electroanalytical Chemistry* **1992**, *331*, 971.
(36) Andrieux, C. P.; Blocman, C.; Dumas-Bouchiat, J. M.; Saveant, J. M. Heterogeneous and homogeneous electron transfers to aromatic halides. An electrochemical redox catalysis study in the halobenzene and halopyridine series. *Journal of the American Chemical Society* **1979**, *101*, 3431.
(37) Hush, N. S. Homogeneous and heterogeneous optical and thermal electron transfer. *Electrochimica Acta* **1968**, *13*, 1005.
(38) Brumberger, H.; Marcus, R. A. Kinetic Study of the Reaction of Diborane with Phosphine. *The Journal of Chemical Physics* **1956**, *24*, 741.
(39) Belèn Meneses, A.; Antonello, S.; Arévalo, M. C.; Maran, F. Double-Layer Correction for Electron-Transfer Kinetics at Glassy Carbon and Mercury Electrodes in N,N-Dimethylformamide. *Electroanalysis* **2006**, *18*, 363.
(40) Bard, A. J.; Faulkner, L. R.; White, H. S. *Electrochemical methods: fundamentals and applications*; John Wiley & Sons, 2022.
(41) Fawcett, W. R. Fifty years of studies of double layer effects in electrode kinetics—a personal view. *Journal of Solid State Electrochemistry* **2011**, *15*, 1347.
(42) Iron, M. A.; Cohen, R.; Rybtchinski, B. On the Unexpected Stability of the Dianion of Perylene Diimide in Water—A Computational Study. *The Journal of Physical Chemistry A* **2011**, *115*, 2047.
(43) Shibano, Y.; Umeyama, T.; Matano, Y.; Tkachenko, N. V.; Lemmetyinen, H.; Araki, Y.; Ito, O.; Imahori, H. Large reorganization energy of pyrrolidine-substituted perylenediimide in electron transfer. *The Journal of Physical Chemistry C* **2007**, *111*, 6133.
(44) Pederserf, S. U.; Lundb, T. Homogeneous Rate Constants for Coupling between Electrochemically Generated Aromatic Anion Radicals and Alkyl Radicals. *Acta Chemica Scandinavia* **1991**, *45*, 397.
(45) Pedersen, S. U.; Lund, T.; Daasbjerg, K.; Pop, M.; Fussing, I.; Lund, H. Kinetic studies of the homogeneous coupling reaction between electrochemically generated aromatic radical anions and alkyl radicals. *Acta Chemica Scandinavica* **1998**, *52*, 657.
(46) Valencia, D. P.; González, F. J. Estimation of diffusion coefficients by using a linear correlation between the diffusion coefficient and molecular weight. *Journal of Electroanalytical Chemistry* **2012**, *681*, 121.
(47) Koefoed, L.; Vase, K. H.; Stenlid, J. H.; Brinck, T.; Yoshimura, Y.; Lund, H.; Pedersen, S. U.; Daasbjerg, K. On the Kinetic and Thermodynamic Properties of Aryl Radicals Using Electrochemical and Theoretical Approaches. *ChemElectroChem* **2017**, *4*, 3212.
(48) Costentin, C.; Fortage, J.; Collomb, M.-N. Electrophotocatalysis: Cyclic Voltammetry as an Analytical Tool. *The Journal of Physical Chemistry Letters* **2020**, *11*, 6097.
(49) Costentin, C.; Robert, M.; Savéant, J.-M. Fragmentation of Aryl Halide π Anion Radicals. Bending of the Cleaving Bond and Activation vs Driving Force Relationships. *Journal of the American Chemical Society* **2004**, *126*, 16051.
(50) Branchi, B.; Galli, C.; Gentili, P. Reactivity of Aryl and Vinyl Radicals: Abstraction of Hydrogen Atom or Reaction with a Nucleophile. *European Journal of Organic Chemistry* **2002**, *2002*, 2844.
